# Ubiquitin-specific protease 38 modulates atrial fibrillation susceptibility in chronic kidney disease via STRAP stabilization and activation of TGF-β/SMAD signaling

**DOI:** 10.1186/s10020-025-01296-1

**Published:** 2025-06-13

**Authors:** Hong Meng, Zongze Qu, Zheng Xiao, Bin Kong, Hongjie Yang, Wei Shuai, He Huang

**Affiliations:** 1 Department of Cardiology, Renmin Hospital of Wuhan University, 238 Jiefang Road, Wuhan, 430060 Hubei P.R. China; 2 Cardiovascular Research Institute of Wuhan University, Wuhan, 430060 Hubei P.R. China; 3 Hubei Key Laboratory of Cardiology, Wuhan, 430060 Hubei P.R. China

**Keywords:** Chronic kidney disease, Atrial fibrillation, USP38, STRAP, TGF-β/SMAD signaling

## Abstract

**Objective:**

This study aimed to elucidate the role of the deubiquitinase USP38 in chronic kidney disease (CKD)-associated atrial fibrillation (AF) by investigating its impact on atrial structural and electrical remodeling and its interaction with STRAP and TGF-β/SMAD signaling.

**Methods:**

A murine CKD model was established using a two-stage 5/6 nephrectomy. Cardiomyocyte-specific USP38 knockout (USP38-CKO) and overexpression (USP38-TG) mice were generated. Atrial remodeling, electrophysiological parameters, and fibrosis markers were assessed by echocardiography, histology, and immunoblotting. In parallel, HL-1 cells were treated with indoxyl sulfate (100 μM) and subjected to adenoviral-mediated USP38 modulation. Molecular interactions between USP38 and STRAP were evaluated using immunofluorescence, co-immunoprecipitation, and ubiquitination assays. STRAP knockdown studies further validated the downstream effects of USP38.

**Results:**

CKD induced significant upregulation of USP38 in atrial tissue and HL-1 cells. USP38-CKO attenuated atrial fibrosis and reduced collagen I/III and α-SMA expression, whereas USP38-TG exacerbated these effects. Notably, USP38 modulation did not significantly alter atrial effective refractory period, suggesting its primary involvement in structural rather than direct electrical remodeling. Mechanistic studies revealed that USP38 stabilizes STRAP via deubiquitination, thereby enhancing TGF-β/SMAD signaling. STRAP knockdown reversed the pro-fibrotic and arrhythmogenic effects induced by USP38 overexpression.

**Conclusion:**

USP38 is a critical mediator of CKD-associated AF, promoting atrial fibrosis and electrical remodeling via STRAP stabilization and TGF-β/SMAD pathway activation. Targeting USP38 may represent a novel therapeutic strategy for CKD-associated AF.

**Supplementary Information:**

The online version contains supplementary material available at 10.1186/s10020-025-01296-1.

## Introduction

Atrial fibrillation (AF) is the most common sustained cardiac arrhythmia encountered in clinical practice and is a major contributor to adverse cardiovascular outcomes, including heart failure, stroke, and renal dysfunction (Shi et al. 2022). Importantly, AF frequently coexists with chronic kidney disease (CKD) (Genovesi et al. 2024), with both conditions mutually increasing the risk of stroke and cardiovascular mortality (Zimmerman et al. 2012; Genovesi et al. 2008).

Although uremic toxins associated with CKD are known to induce atrial structural and electrical remodeling (Song et al. 2023; Sidaway 2014), the molecular mechanisms linking renal dysfunction to AF remain unclear. Recent evidence indicates that ubiquitin-specific proteases (USPs) are critical regulators of protein stability in cardiovascular diseases (Xie et al. 2024; Wang et al. 2024). Our prior work demonstrated that USP38 enhances AF susceptibility by promoting electrical and structural remodeling in models of pressure overload and diabetes-induced cardiac dysfunction (Xiao et al. 2024a, b). However, CKD patients also exhibit electrolyte imbalances, uremic toxin accumulation, systemic inflammation, and myocardial fibrosis (Turakhia et al. 2018; Qiu et al. 2018)—factors that independently contribute to atrial remodeling (Song et al. 2023). Notably, the role of USP38 in CKD-associated AF has not yet been elucidated.

Furthermore, although the transforming growth factor-β (TGF-β)/SMAD signaling pathway is recognized as a key driver of atrial fibrosis (Saljic et al. 2022; Lai et al. 2022), the upstream regulators of this pathway under CKD conditions remain poorly defined. Intriguingly, serine-threonine kinase receptor-associated protein (STRAP) has been shown to potentiate TGF-β signaling and promote disease progression (Zhang et al. 2023). We hypothesize that USP38 exacerbates CKD-induced atrial remodeling and AF by stabilizing STRAP via deubiquitination, thereby amplifying the TGF-β/SMAD cascade. In this study, we utilize in vivo CKD models with cardiomyocyte-specific modulation of USP38 to delineate its role in AF susceptibility.

## Methods

Detailed experimental protocols are provided in the Supplementary Methods.

### Animals and CKD model

All animal procedures were performed in accordance with NIH guidelines and approved by the Institutional Animal Care and Use Committee of Renmin Hospital, Wuhan University (Approval No.: WDRM20250404C). To establish a CKD model, a two-stage 5/6 nephrectomy (5/6 Nx) was performed on 8–10-week-old male C57BL/6 mice as previously described (Song et al. 2023). Briefly, mice were anesthetized with sodium pentobarbital (50 mg/kg, i.p.) and approximately two-thirds of the left kidney was resected via a flank incision. One week later, the right kidney was completely excised. Postoperative care included subcutaneous administration of buprenorphine (0.1 mg/kg) for analgesia and enrofloxacin (5 mg/kg) for infection prophylaxis for 3 days. Mice were then maintained under controlled conditions (22 ± 2 °C, 12-hour light/dark cycle) for 4 weeks to allow for CKD progression prior to functional and molecular assessments.

### Statistical analysis

Data were analyzed using GraphPad Prism version 10.0 (GraphPad Software, USA) and SPSS version 27.0 (IBM, USA). Continuous variables are presented as the mean ± standard error of the mean (SEM). Normality was assessed using the Shapiro-Wilk test. For normally distributed data, comparisons between two groups were performed using an unpaired, two-tailed Student’s t-test with Welch’s correction for unequal variances, while multiple-group comparisons were conducted using one-way ANOVA followed by Tukey’s post hoc test. Nonparametric data were analyzed using the Wilcoxon-Mann-Whitney U test, and categorical variables were compared using Fisher’s exact test. A two-tailed *P* value of < 0.05 was considered statistically significant.

## Results

### CKD induces atrial USP38 upregulation

**Fig. 1:**
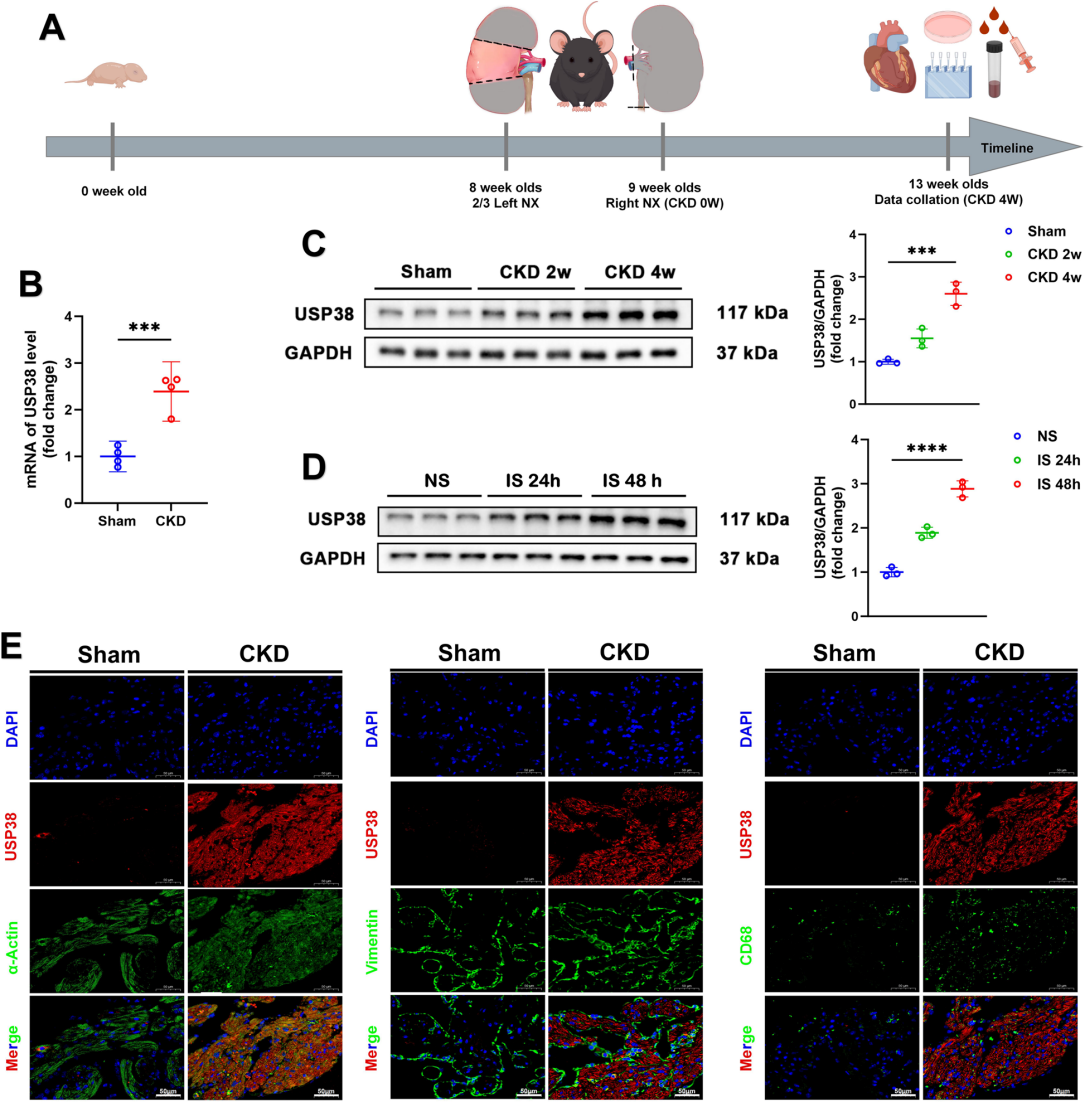
CKD upregulates USP38 expression in the left atrium of mice. **A** Experimental timeline for CKD induction via two-stage 5/6 nephrectomy (5/6 Nx). **B** RT-qPCR analysis of USP38 mRNA expression in atrial tissue (*n* = 4). **C** Representative western blot (WB) images and densitometric quantification of USP38 protein levels in wild-type mice 2 and 4 weeks post-5/6 Nx (*n* = 3). **D** Representative WB images and USP38 protein expression of HL-1 cells treated with saline or indoxyl sulfate (IS) for 24 h and 48 h (*n* = 3). **E** Confocal microscopy images of USP38 (red) co-stained with α-actin (cardiomyocytes, green), vimentin (fibroblasts, green), or CD68 (macrophages, green) in left atrial tissue at 4 weeks post-sham or 5/6 Nx surgery (scale bars: 50 μm). (*** *p* <0.001, **** *p* <0.0001.)

To investigate the upstream triggers of atrial USP38 upregulation in the setting of chronic kidney disease (CKD), we utilized a progressive 5/6 nephrectomy (5/6 Nx) mouse model (Fig. 1A). Consistent with renal dysfunction (Supplementary Figure S1A-F), USP38 mRNA and protein levels in atrial tissue increased progressively over time (Fig. 1B–C), suggesting a time-dependent response to CKD. Given the known accumulation of uremic toxins in CKD, we next tested whether reducing these circulating toxins would impact USP38 expression. Treatment with AST-120, an oral adsorbent that lowers serum indoxyl sulfate (IS) and other protein-bound uremic toxins (Tsai et al. 2023), significantly attenuated the CKD-induced upregulation of USP38 in vivo (Supplementary Figure S1G). These results further confirm that CKD is a key factor in inducing USP38 expression in cardiomyocytes.

To further investigate this mechanism in vitro, we treated HL-1 cardiomyocytes with indoxyl sulfate (IS), a prototypical protein-bound uremic toxin implicated in cardiovascular remodeling (Shen et al. 2021). IS exposure resulted in time-dependent upregulation of USP38 protein (Fig. 1D), supporting a direct regulatory effect of IS on cardiomyocyte USP38 expression. Immunofluorescence analysis confirmed that USP38 induction occurred predominantly in cardiomyocytes, with minimal expression in non-cardiomyocytes (Fig. 1E). Together, these in vivo and in vitro findings demonstrate that CKD-induced uremic toxins—particularly IS—are sufficient to drive USP38 upregulation in cardiomyocytes, and that systemic IS reduction can mitigate this effect. These data identify IS as a plausible soluble upstream effector contributing to CKD-associated pro-arrhythmogenic remodeling via USP38.

### USP38 modulates AF susceptibility in CKD

**Fig. 2:**
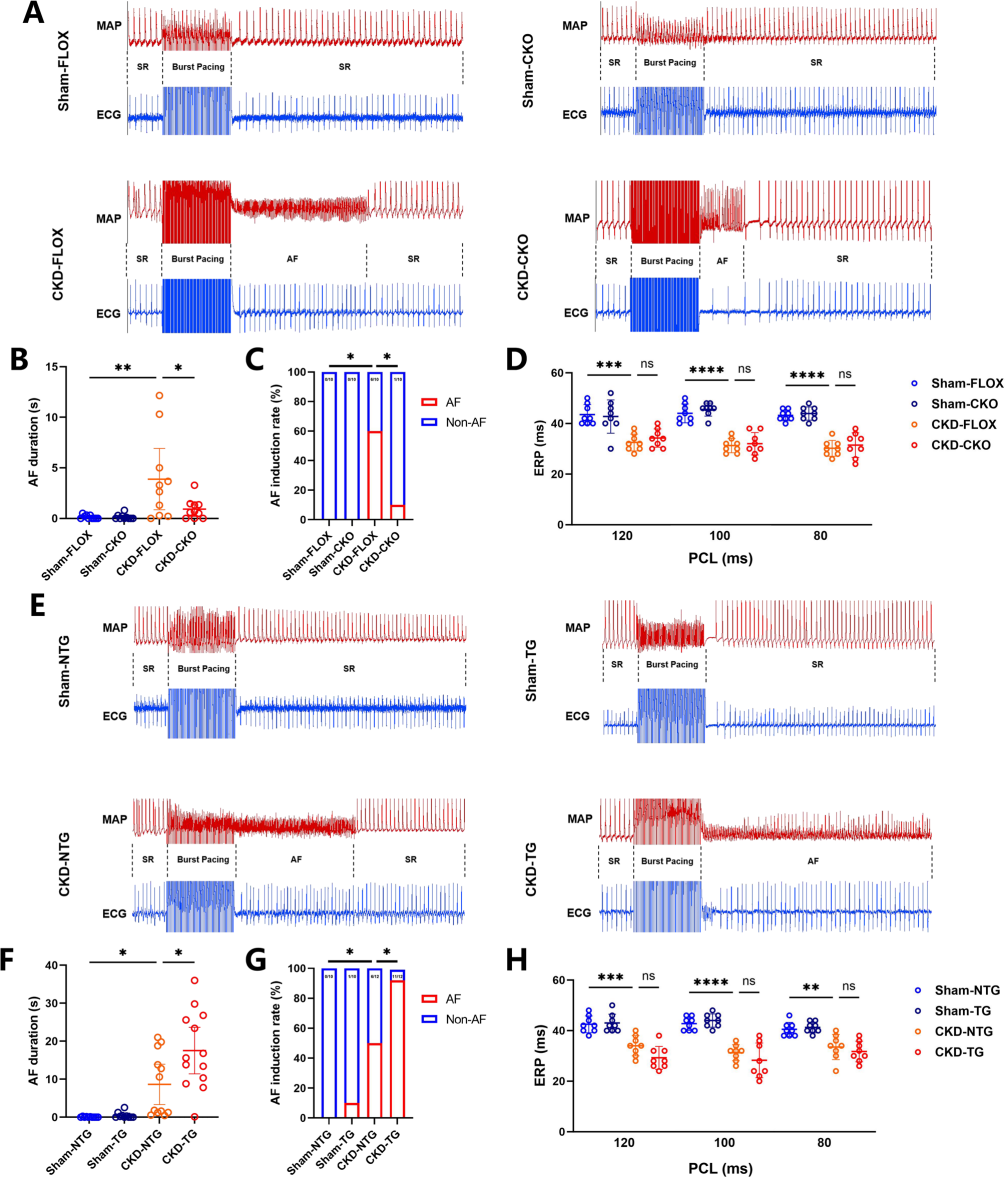
USP38 modulates AF susceptibility in CKD mice. **A** Representative ECG tracings and action potential recordings during AF induction in FLOX and CKO mice. **B**, **C** AF induction duration and incidence in FLOX and CKO cohorts (*n* = 10). **D** Atrial effective refractory period (AERP) measurements in FLOX and CKO mice (*n* = 8). **E** Representative ECG tracings and action potential recordings during AF induction in NTG and TG mice. **F**, **G** AF induction duration and incidence in NTG and TG mice (*n* = 10–12). **H** AERP measurements in NTG and TG mice (*n* = 8). (ns no statistical difference, * *p* <0.05, ** *p* <0.01, *** *p* <0.001)

To elucidate the role of USP38 in modulating AF susceptibility in CKD, we generated cardiomyocyte-specific USP38 knockout (USP38-CKO) and overexpression (USP38-TG) mice (Supplementary Methods). The absence of USP38 protein in the heart was confirmed in USP38-CKO mice, whereas a marked overexpression was observed in USP38-TG hearts (Supplementary Figure S2). CKD mice exhibited increased AF inducibility relative to sham controls, and USP38 significantly altered atrial fibrillation susceptibility in CKD mice (Fig. 2). In the CKD model, a significantly shortened atrial effective refractory period (AERP) was observed (Fig. 2), indicating impaired atrial electrophysiological function. However, both USP38-CKO and USP38-TG mice maintained CKD-induced AERP shortening without any significant improvement or exacerbation compared to the CKD-alone group (Fig. 2D, H). These findings suggest that USP38 influences CKD-related AF susceptibility via mechanisms independent of direct AERP modulation. Moreover, baseline ECG parameters did not differ significantly among the groups (Supplementary Figure S3), further excluding baseline electrical differences as a confounding factor.

To deeply analyze the direct association between USP38 and AF phenotypes, we further detected the USP38 protein levels in the hearts of mice with different heart rhythm phenotypes under the background of CKD. The results showed that the expression level of USP38 in the AF rhythm groups of the FLOX/CKO and NTG/TG strains was significantly higher than that in the sinus rhythm maintenance group (Supplementary Figure S4). This indicates that the upregulation of USP38 protein level is directly correlated with the occurrence of AF. Therefore, all the above results consistently indicate that USP38 significantly increases the susceptibility to AF in CKD mice.

### USP38 drives atrial structural remodeling

**Fig. 3:**
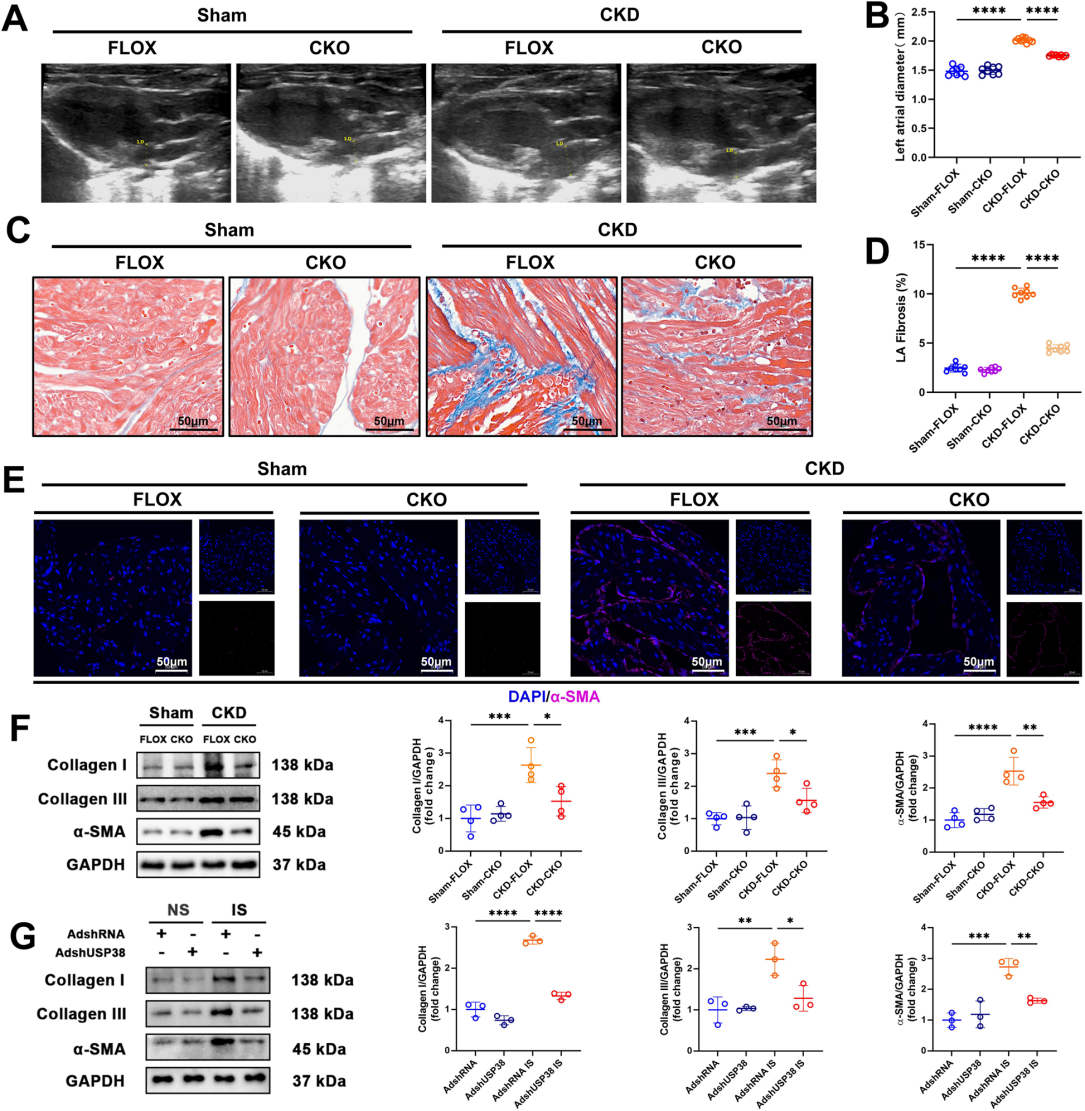
Cardiomyocyte-specific USP38 knockout mitigates CKD-induced atrial structural remodeling. **A**, **B** Representative echocardiographic images and quantification of left atrial (LA) diameter in sham/CKD FLOX and CKO mice (*n* = 8). **C**, **D** Masson’s trichrome-stained atrial sections (collagen, blue) and statistical analysis of the proportion of left atrial fibrosis area (*n* = 8; scale bars: 50 μm). **E** α-SMA immunofluorescence (myofibroblasts, purple) in atrial tissue (scale bars: 50 μm). **F** Representative western blot (WB) image and analysis of collagen I, collagen III, and α-SMA protein levels in left atrial tissue (*n* = 4). **G** Representative WB image and analysis of collagen I, collagen III, and α-SMA protein levels in HL-1 cell (*n* = 3). (* *p* < 0.05, ** *p* < 0.01, *** *p* <0.001, **** *p* <0.0001)

**Fig. 4:**
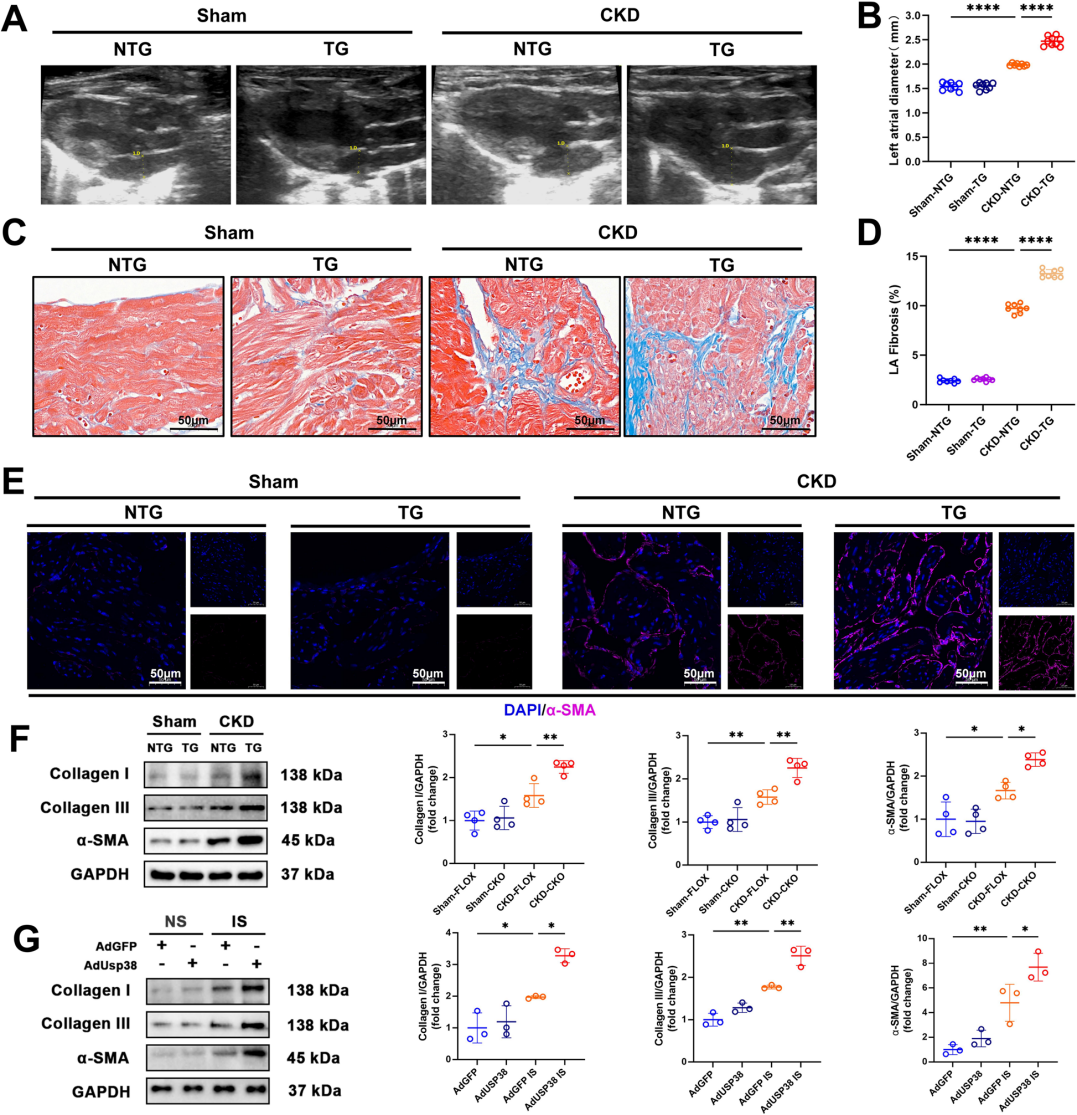
Cardiomyocyte-specific USP38 overexpression exacerbates CKD-induced atrial remodeling. **A**, **B** Representative echocardiographic analysis of LA diameter in sham/CKD NTG and TG mice (*n* = 8). **C**, **D** Masson’s trichrome-stained atrial sections (collagen, blue) and statistical analysis of the proportion of left atrial fibrosis area (*n* = 8; scale bars: 50 μm). **E** α-SMA immunofluorescence (scale bars: 50 μm). **F** Representative western blot (WB) image and analysis of collagen I, collagen III, and α-SMA protein levels in left atrial tissue (*n* = 4). **G** Representative WB image and analysis of collagen I, collagen III, and α-SMA protein levels in HL-1 cell (*n*=3). (* *p* < 0.05, ** *p* < 0.01, *** *p* <0.001, **** *p* <0.0001)

Structural remodeling of the left atrium is strongly associated with the development and progression of AF, particularly in the context of CKD, which induces both mechanical and fibrotic changes in atrial tissue (Aronson et al. 2025). We assessed atrial remodeling at both macroscopic and microscopic levels using multiple methodologies. Echocardiography revealed significant left atrial (LA) dilation in CKD mice (Fig. 3A, B and Supplementary Figure S5A), which was notably attenuated in USP38-CKO mice. In contrast, ventricular systolic (left ventricular ejection fraction, left ventricular fractional shortening) and diastolic (E/A ratio) functions remained unchanged (Supplementary Figure S5B-F). Histological analysis demonstrated pronounced atrial fibrosis in CKD mice (Fig. 3C, D), which was mitigated by USP38 ablation. Consistently, immunoblotting showed that USP38-CKO suppressed the CKD-induced upregulation of α-SMA and collagen I/III (Fig. 3E, F). Subsequently, HL-1 cells transfected with adenovirus-mediated USP38 knockdown and treated with IS exhibited a marked reduction in collagen I, collagen III, and α-SMA protein levels compared with control cells (Fig. 3G). Conversely, USP38-TG mice displayed exacerbated LA dilation and fibrosis (Fig. 4A-F and Supplementary Figure S5G) without affecting ventricular function (Supplementary Figure S5H-L), underscoring the atrial-specific pathological role of USP38. Similarly, HL-1 cells overexpressing USP38 via adenoviral transfection followed by IS treatment demonstrated significant upregulation of collagen I, collagen III, and α-SMA compared with controls (Fig. 4G). These results support our initial hypothesis that USP38 exacerbates CKD-associated atrial remodeling, and align with prior findings linking fibrosis and atrial enlargement to AF vulnerability.

### USP38 exacerbates gap junction remodeling and calcium handling abnormalities in atrial tissue

To further elucidate the mechanisms underlying atrial electrical remodeling in CKD, we investigated the role of USP38 in gap junction integrity and calcium-handling abnormalities, both of which are key contributors to AF (Xiao et al. 2024a, b; Pan et al. 2024).

**Fig. 5:**
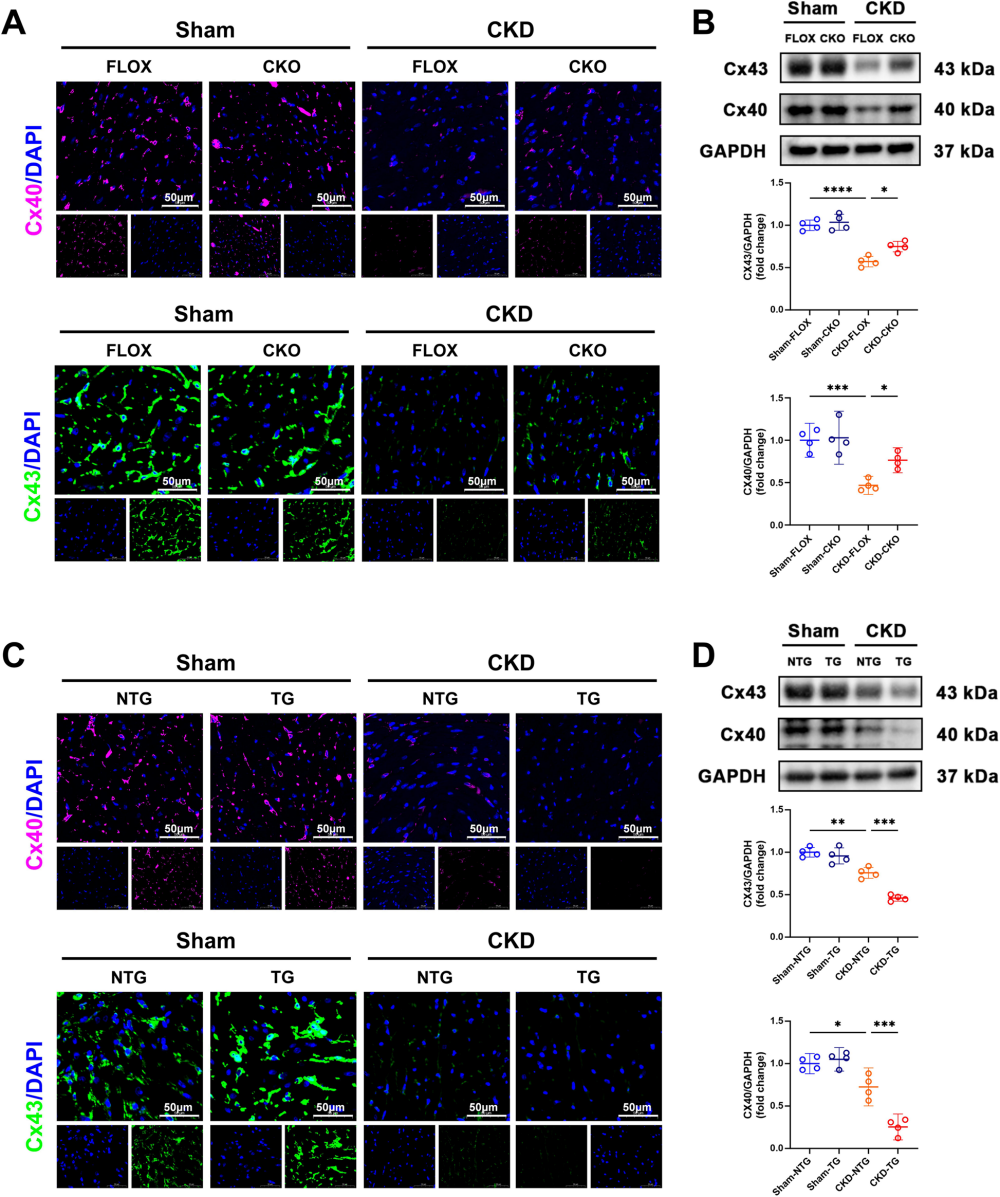
USP38 disrupts gap junction protein expression. **A** Immunofluorescence of connexin 40 (Cx40) and Cx43 in sham/CKD FLOX and CKO mice (scale bars: 50 μm). **B** Western blot (WB) quantification of Cx40 and Cx43 levels (*n* = 4). **C** Cx40/Cx43 immunofluorescence in sham/CKD NTG and TG mice (scale bars: 50 μm). **D** WB quantification of Cx40 and Cx43 levels in sham/CKD NTG and TG mice (*n* = 4). (* *p* < 0.05, ** *p* < 0.01, *** *p* <0.001, **** *p* <0.0001)

Connexins are key components of intercalated discs and are essential for proper electrical conduction (Li et al. 2021). Alterations in connexin expression can disrupt cardiomyocyte electrical coupling, leading to conduction blocks, reentrant circuits, and arrhythmias (Andelova et al. 2020). Furthermore, myocardial fibers may influence cardiac electrophysiology via both gap junction coupling and fibrosis (Jakob et al. 2021). Consistent with these findings, and the concept that structural remodeling and gap junction disruption jointly contribute to arrhythmogenesis in CKD. Our data revealed that CKD significantly downregulated the expression of connexin 40 (Cx40) and connexin 43 (Cx43), which are critical for intercellular electrical communication. Notably, USP38-CKO partially restored Cx40/Cx43 protein levels (Fig. 5A, B), whereas USP38-TG further suppressed their expression (Fig. 5C, D), thereby implicating USP38 in gap junction remodeling.

Given the central role of calcium homeostasis in electrical stability, we next examined calcium-handling abnormalities. AF is commonly associated with dysregulated intracellular calcium transients and increased diastolic sarcoplasmic reticulum (SR) Ca^2+^ leak, which are largely mediated by altered expression and function of key calcium-handling proteins (Xiao et al. 2024a, b). We assessed the levels of ryanodine receptor 2 (RyR2), SR Ca-ATPase 2a (SERCA2a), and phospholamban (PLB). Under pressure overload conditions, we observed increased phosphorylation of RyR2 at Ser2808 and PLB at Thr17, which is indicative of enhanced SR Ca^2+^ leak and impaired calcium reuptake, respectively (Supplementary Figure S6A-D). These abnormalities were markedly attenuated in USP38-CKO mice, suggesting that USP38 deficiency mitigates calcium mishandling. In addition, pressure overload led to a reduction in SERCA2a expression, while USP38 deficiency restored SERCA2a levels compared to CKD-FLOX.

In contrast, USP38-TG mice exhibited exacerbated calcium-handling defects. Phosphorylation of RyR2^Ser2808 and PLB^Thr17 was further increased, and SERCA2a expression was more severely reduced relative to wild-type mice under the same stress conditions (Supplementary Figure S6E-H). These data collectively suggest that USP38 promotes both gap junction remodeling and calcium homeostasis disruption, thereby aggravating atrial electrical instability under pathological stress.

### USP38 activates TGF-β1/SMAD2/3 signaling

**Fig. 6:**
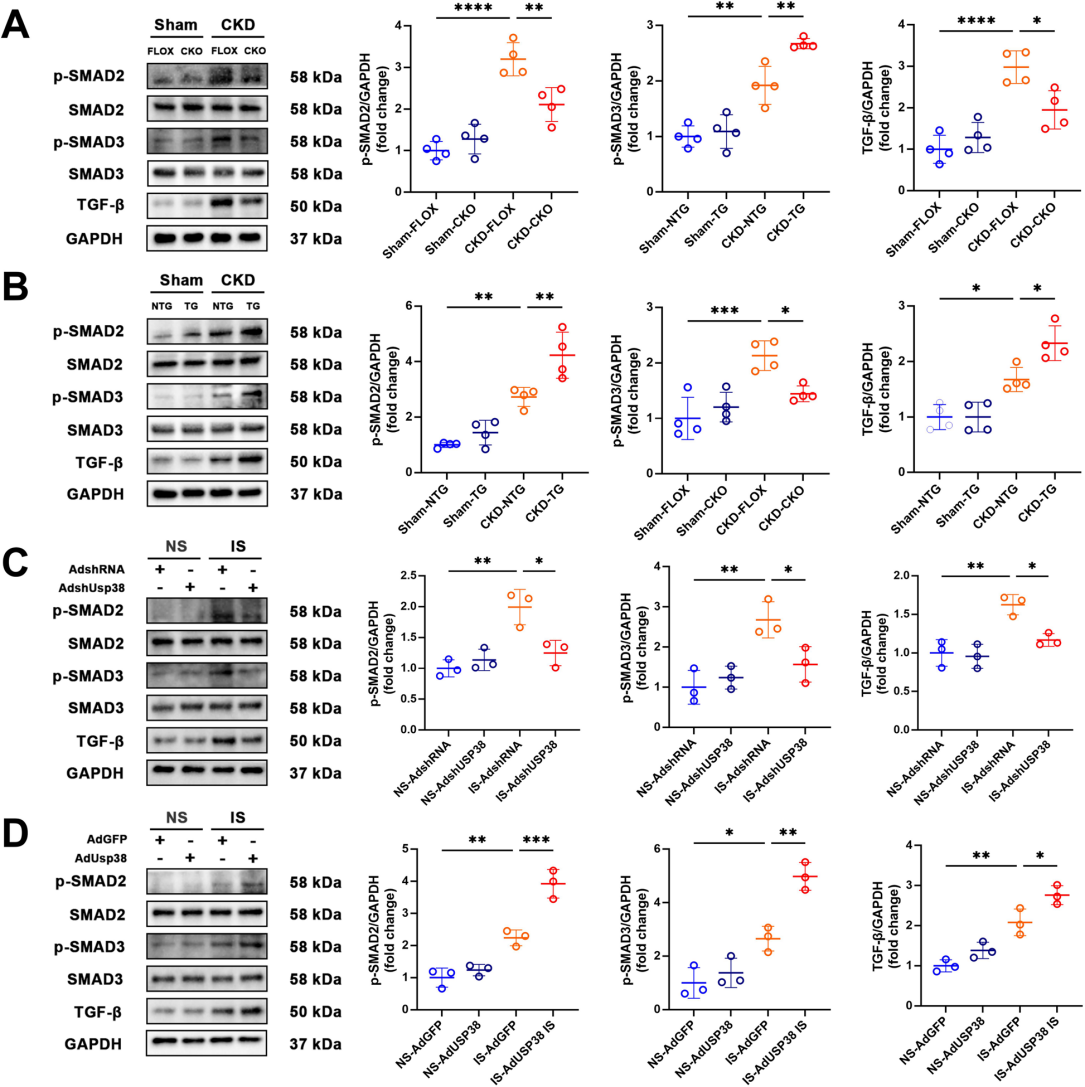
CKD promotes USP38 activation of the TGF-β/SMAD pathway. **A**, **B** Representative western blot (WB) images and statistical analysis of TGF-β, p-SMAD2, and p-SMAD3 protein levels in left atrial tissue of mice at 4 weeks after sham surgery or 5/6 Nx (*n* = 4). **C**, **D** Representative WB images and statistical analysis of TGF-β, p-SMAD2, and p-SMAD3 protein expression levels in HL-1 cells stimulated with normal saline (NS) or indoxyl sulfate (IS) (*n* = 3). (* *p* < 0.05, ** *p* < 0.01, *** *p* <0.001, **** *p* <0.0001)

Myocardial fibrosis is a key contributor to the increased susceptibility to AF following renal failure (Hsu et al. 2023). Previous studies have established that TGF-β plays a pivotal role in promoting atrial fibrosis via activation of classical SMAD signaling pathways (Lin et al. 2017). Building on these findings, and in light of our earlier hypothesis that USP38 may regulate fibrotic signaling cascades, we employed western blot analysis to assess the expression of key TGF-β1/SMAD mediators. Our results demonstrated that CKD upregulated TGF-β1 and phosphorylated SMAD2/3 (p-SMAD2/3) in atrial tissue (Fig. 6A, B). In USP38-CKO mice, these profibrotic mediators were significantly suppressed (Fig. 6A), whereas in USP38-TG mice, their activation was further enhanced (Fig. 6B), thereby linking USP38 to TGF-β–driven fibrogenesis. Furthermore, HL-1 cells treated with IS following adenoviral-mediated USP38 knockdown or overexpression exhibited parallel changes in TGF-β/SMAD pathway proteins (Fig. 6C, D). These findings provide mechanistic evidence supporting USP38 as a positive regulator of atrial fibrosis via TGF-β1/SMAD signaling.

### USP38 stabilizes STRAP via deubiquitination

**Fig. 7:**
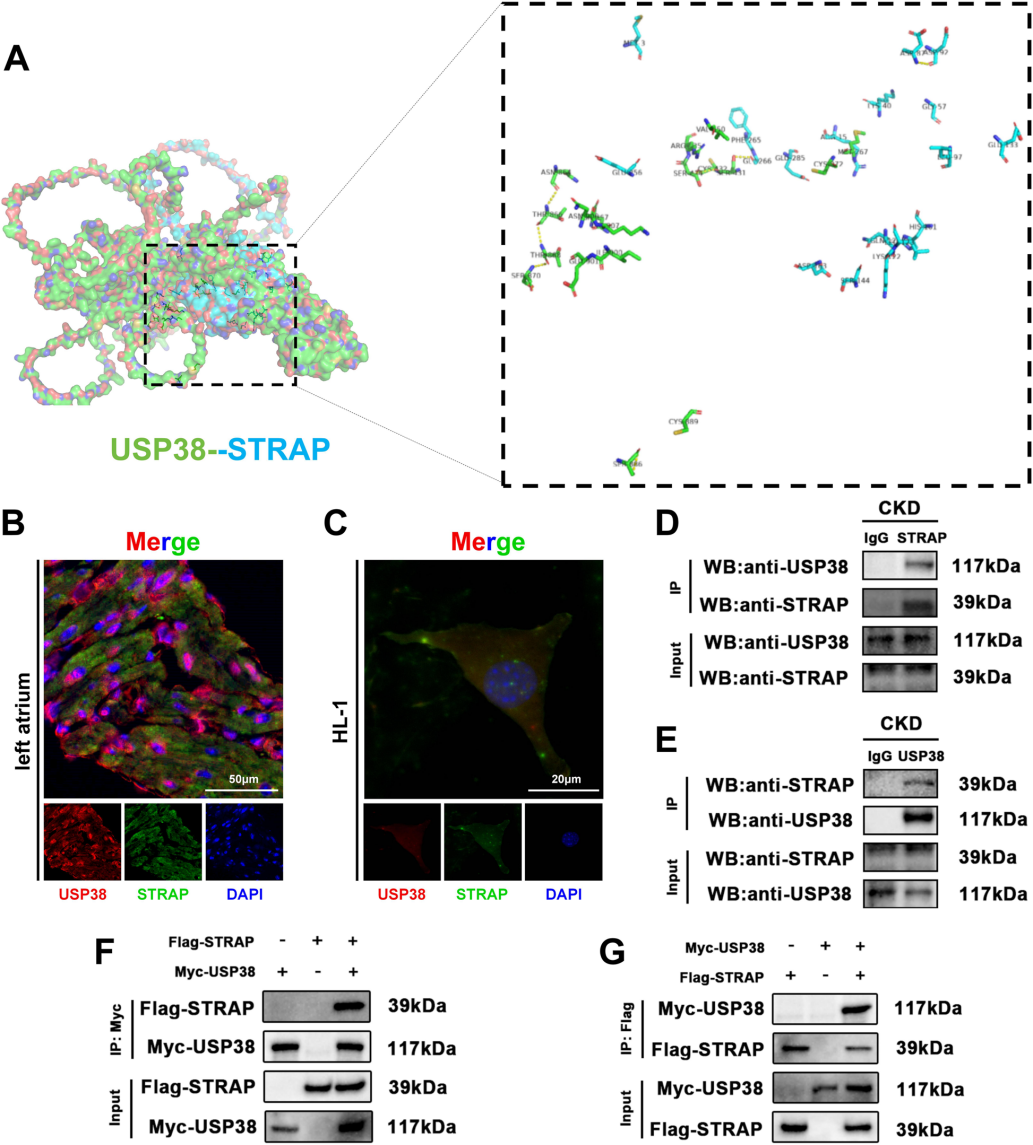
USP38 physically interacts with STRAP to aggravate atrial remodeling. **A** Molecular docking model of USP38-STRAP interaction (PyMOL). **B** Confocal co-localization of USP38 (green) and STRAP (red) in atrial tissue (scale bars: 50 μm). **C** Confocal co-localization of USP38 (green) and STRAP (red) in HL-1 cell (scale bars: 20 μm). **D**-**E** Endogenous co-immunoprecipitation (Co-IP) of USP38 and STRAP in atrial lysates. **F**-**G** Exogenous Co-IP in HEK-293 T cells overexpressing Flag-STRAP and Myc-USP38

**Fig. 8:**
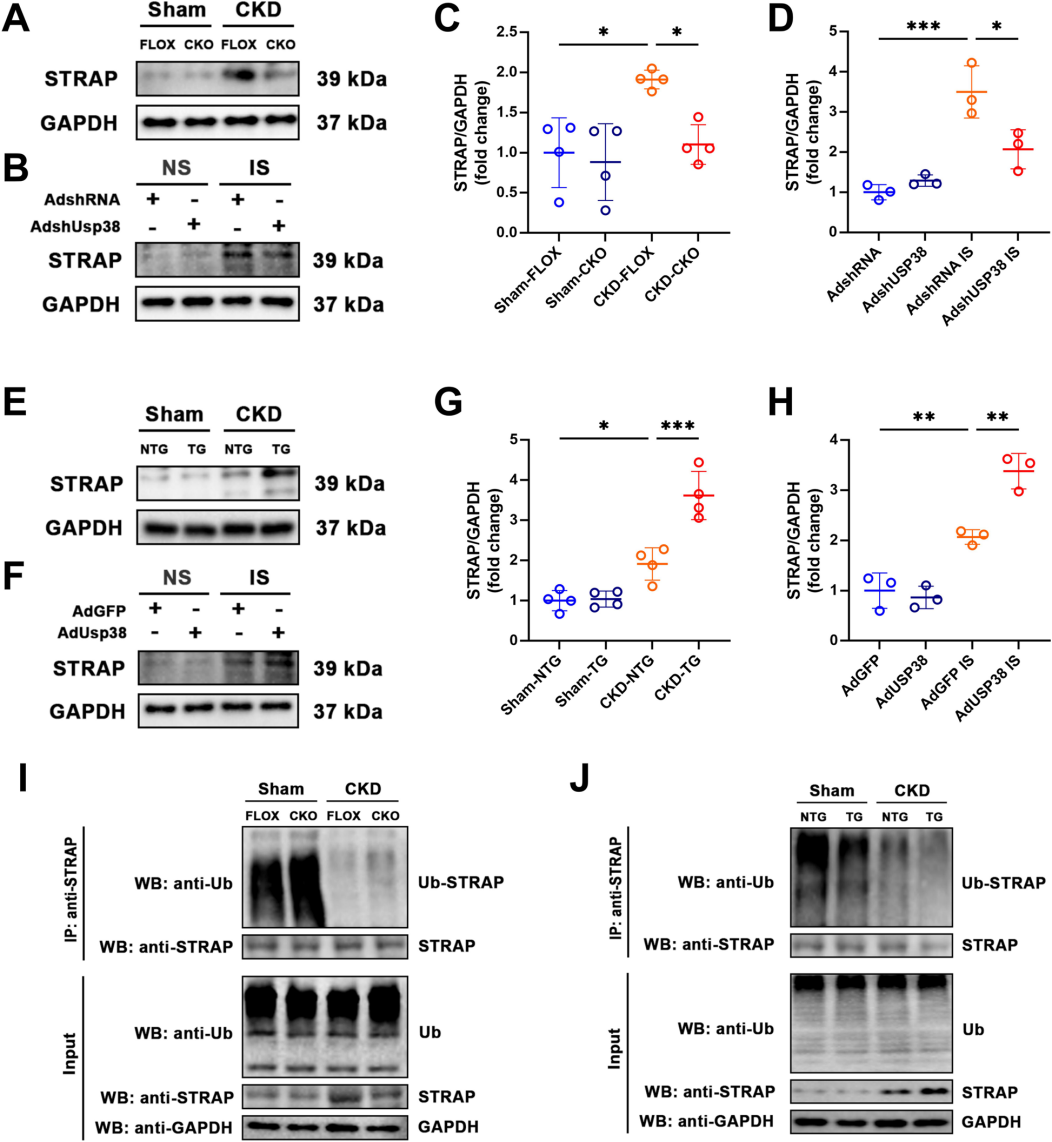
USP38 Stabilizes STRAP levels by deubiquitination. **A**-**D** Representative western blot (WB) images and statistical analysis of STRAP protein levels in left atrial tissues of FLOX and CKO mice at 4 weeks after sham or 5/6 nephrectomy and in HL-1 cells transfected with AdshRNA or AdshUSP38 treated with normal saline (NS) or indoxyl sulfate (IS) (*n* = 4). **E**-**H** Representative WB images and statistical analysis of STRAP protein levels in left atrial tissues of NTG and TG mice at 4 weeks after sham or 5/6 nephrectomy and in HL-1 cells transfected with AdGFP or AdUSP38 treated with NS or IS (*n* = 4). **I** Ubiquitination results of STRAP in left atrial tissue from FLOX and CKO mice at 4 weeks after sham or 5/6 nephrectomy. **J** Ubiquitination results of STRAP in left atrial tissue from NTG and TG mice at 4 weeks after sham or 5/6 nephrectomy. (* *p* < 0.05, *** *p* <.001)

STRAP is a known modulator of TGF-β signaling, and its stabilization has been linked to the amplification of fibrotic responses in cardiac and renal pathologies (Zhang et al. 2023). We therefore hypothesized that USP38 aggravates CKD-induced cardiac remodeling by regulating STRAP. Molecular docking analysis predicted a strong interaction between USP38 and STRAP (binding energy: −15.9 kcal/mol; Fig. 7A). In left atrial tissue, immunofluorescence revealed clear co-localization of USP38 and STRAP (Fig. 7B), and similar co-localization was observed in HL-1 cells via confocal microscopy (Fig. 7C). Subsequent co-immunoprecipitation (Co-IP) experiments conducted in both atrial tissue and HEK-293 T cell lysates transfected with the relevant plasmids confirmed the endogenous and exogenous interaction between USP38 and STRAP (Fig. 7D-G). Notably, treatment of HL-1 cells with indoxyl sulfate (IS), a representative uremic toxin, significantly enhanced the interaction between USP38 and STRAP, as evidenced by increased Co-IP intensity (Supplementary Figure S7), suggesting that pathological stimuli strengthen this interaction. Furthermore, USP38-CKO resulted in decreased STRAP expression in both atrial tissue and HL-1 cells (Fig. 8A-D), whereas USP38-TG led to increased STRAP expression in both models (Fig. 8E-H). Finally, ubiquitination assays revealed that USP38 knockout enhanced STRAP ubiquitination, while USP38 overexpression reduced the formation of Ub-STRAP complexes, thereby supporting our hypothesis that USP38 acts as a deubiquitinase that stabilizes STRAP to sustain TGF-β–mediated fibrotic signaling in CKD (Fig. 8I, J).

### STRAP knockdown reverses USP38-mediated arrhythmogenic phenotypes

**Fig. 9:**
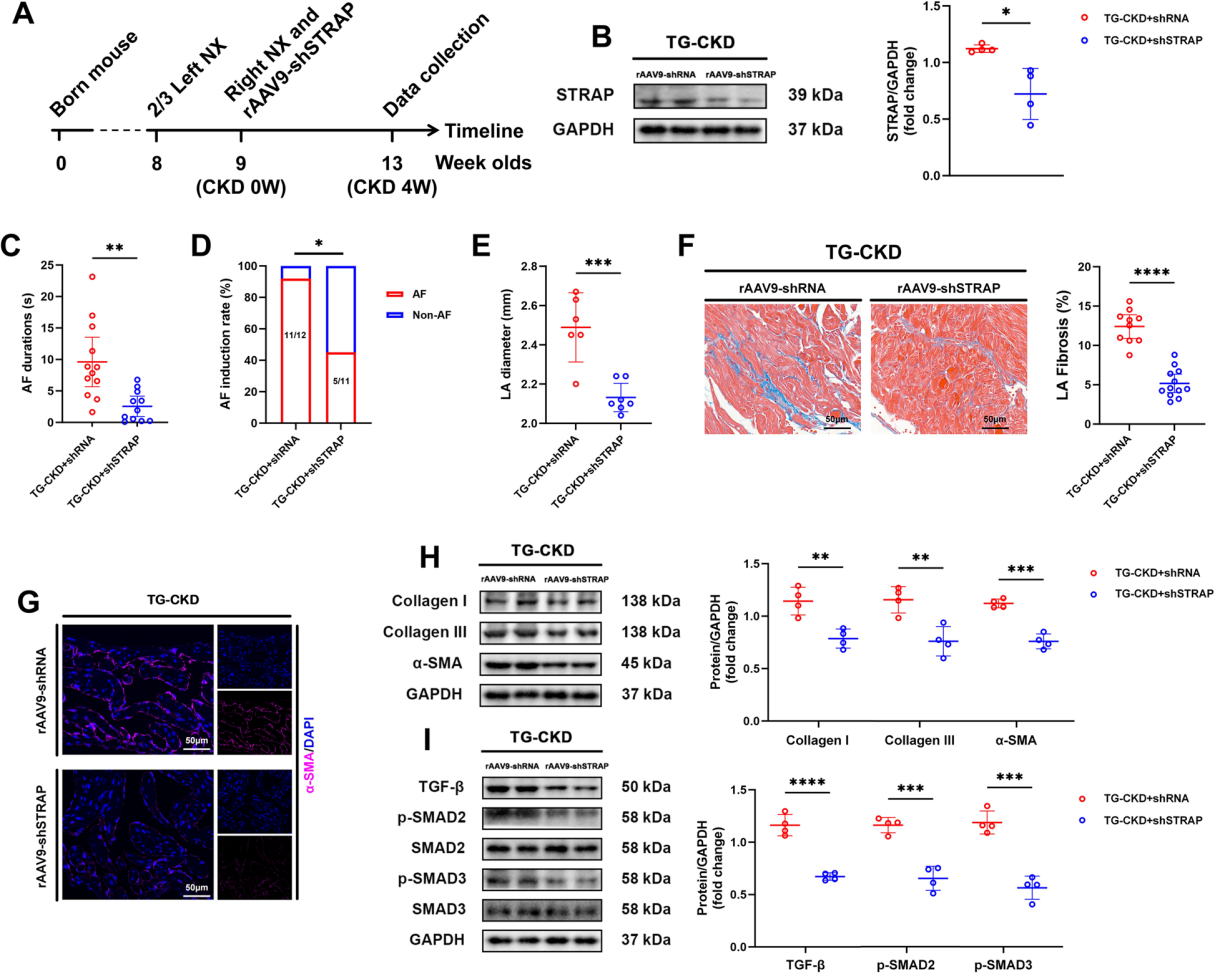
STRAP knockdown reverses USP38-driven atrial remodeling. **A** Experimental timeline for AAV9-shSTRAP delivery. **B** Western blot (WB) validation of STRAP knockdown in TG mice (*n* = 4). **C**, **D** AF induction duration and incidence (*n* = 11–12). **E** LA diameter quantification (*n* = 6–7). **F** Masson’s staining and statistical analysis of the proportion of fibrosis area (*n* = 10–12; scale bars: 50 μm). **G** α-SMA immunofluorescence (scale bars: 50 μm). **H**, **I** WB analysis of fibrosis markers and TGF-β/SMAD pathway components (*n* = 4) (* *p* < 0.05, ** *p* < 0.01, *** *p* <.001, **** *p* <.0001)

To determine whether STRAP mediates the effects of USP38 on AF susceptibility in CKD, we employed AAV9-cTnT-shRNA to knock down STRAP expression in CKD-TG mice (Fig. 9A, B). AAV9-mediated cardiac STRAP silencing in these mice significantly attenuated AF inducibility and reduced LA dilation (Fig. 9C-E). Moreover, the expression of fibrosis biomarkers (collagen I/III, α-SMA) and key components of TGF-β/SMAD signaling (TGF-β, p-SMAD2/3) was markedly suppressed (Fig. 9F-I), thereby confirming that STRAP functions as a critical downstream effector of USP38-driven pathology.

## Discussion

Our study provides the first evidence that the deubiquitinase USP38 plays a central role in mediating CKD-induced atrial structural remodeling. Specifically, we demonstrated that CKD mice exhibit a marked upregulation of USP38 in atrial tissue, and that cardiomyocyte-specific USP38 knockout (USP38-CKO) significantly attenuates atrial fibrosis and AF susceptibility. In contrast, USP38 overexpression further exacerbates these pathological phenotypes. Mechanistically, USP38 stabilizes STRAP via deubiquitination, thereby amplifying TGF-β/SMAD signaling to drive fibrogenesis, while simultaneously disrupting gap junction integrity—ultimately promoting structural abnormalities of the myocardium and increasing AF susceptibility.

According to the World Health Organization, AF is one of the most common clinical arrhythmias, affecting over 30 million people worldwide (Chugh et al. 2014). AF is a major contributor not only to heart failure and ischemic stroke but also to increased all-cause and cardiovascular mortality (Chyou et al. 2025). Notably, driven by rapid population aging, the prevalence of AF has surged by 146% over the past 30 years (Shi et al. 2022), and the associated healthcare costs now pose a significant public health challenge. Concurrently, chronic kidney disease (CKD)—a condition closely linked to aging (Watanabe et al. 2008; Park et al. 2020)—exhibits strong epidemiological and pathophysiological associations with AF (Soliman et al. 2010; Hawkins et al. 2016). Studies confirm that renal dysfunction is an independent risk factor for AF, and conversely, patients with AF are at heightened risk for renal impairment (Böhm et al. 2015). In fact, 15–20% of CKD patients develop AF, a condition associated with increased mortality (Hart et al. 2013; Zimmerman et al. 2012), while 40–50% of AF patients experience progressive renal dysfunction over time (Banerjee et al. 2014; Fauchier et al. 2018).

Recent studies further reveal that CKD and AF interact in a vicious cycle (Bansal et al. 2013; Bansal et al. 2016). On one hand, uremic toxins associated with CKD directly promote AF by inducing myocardial fibrosis and electrical remodeling (Gao et al. 2024). On the other hand, reduced cardiac output and impaired renal perfusion resulting from AF can accelerate CKD progression (Chen et al. 2022). Therefore, elucidating the molecular mechanisms underlying the crosstalk between CKD and AF is of critical clinical importance for developing targeted therapeutic strategies.

Previous research has predominantly focused on systemic CKD-related pathological factors that promote AF (Song et al. 2023; Qiu et al. 2018), whereas the atrial-specific molecular events remain poorly understood. Our previous studies identified USP38 as a key regulator of cardiac remodeling (Pan et al. 2024; Xiao et al. 2024c). Building on these findings, the present study employed a 5/6 Nx-induced CKD mouse model to demonstrate that USP38 is specifically upregulated in atrial tissue. Furthermore, cardiomyocyte-specific knockout of USP38 significantly reduced AF inducibility, while its overexpression exacerbated AF susceptibility. Mechanistically, CKD-induced uremic toxins upregulate USP38 at the transcriptional level, thereby driving cardiac remodeling.

Beyond electrophysiological abnormalities, the extent of atrial fibrosis in CKD patients is strongly correlated with AF susceptibility (Hsu et al. 2023; Hohl et al. 2022). Our study confirms that USP38 acts as a pivotal mediator of CKD-driven atrial structural remodeling: USP38 knockout markedly reduced collagen deposition and attenuated left atrial dilation, whereas its overexpression exacerbated fibrotic progression. At the molecular level, CKD-associated uremic toxins amplify oxidative stress, inflammation, and neurohormonal activation, thereby contributing to cardiovascular fibrosis and oxidative damage (Sun et al. 2012). Moreover, uremic toxins directly induce cardiomyocyte injury, promoting hypertrophic responses and fibrotic changes (Lekawanvijit et al. 2010). Injured cardiomyocytes, in turn, activate paracrine signaling pathways that stimulate fibroblast activation and enhance collagen secretion.

Furthermore, connexins—key mediators of intercellular communication—have been implicated in the proarrhythmic effects associated with renal dysfunction (Sidaway 2014; Sinha et al. 2023; Price et al. 2020), with TGF-β signaling playing a central role (Potter et al. 2021). Notably, our study is the first to demonstrate that USP38 modulates connexin expression, thereby contributing to electrical remodeling in this context.

Moreover, we discovered that USP38 stabilizes STRAP through deubiquitination. As a TGF-β co-receptor, STRAP facilitates SMAD2/3 phosphorylation and accelerates fibrogenesis (Zhang et al. 2023). This is the first study to establish a direct link between a deubiquitinase and STRAP regulation, thereby filling a critical gap in the upstream control of TGF-β signaling. In contrast to other USP family members such as USP4—which enhances TGF-β pathway activity by deubiquitinating TGF-β receptors (Zhu et al. 2019)—USP38 selectively targets STRAP, highlighting the functional heterogeneity within the USP family in regulating fibrotic pathways.

In summary, our study is the first to elucidate the central role of USP38 in the pathogenesis of CKD-associated AF. By integrating in vivo and in vitro experiments, we have systematically delineated how USP38 amplifies TGF-β/SMAD signaling via STRAP stabilization, leading to both atrial structural remodeling. Under CKD conditions, USP38 is upregulated and maintains STRAP stability through targeted deubiquitination, ultimately exacerbating atrial fibrotic substrate, thereby markedly increasing AF susceptibility. Consequently, therapeutic strategies that target USP38 or its downstream effectors—such as small-molecule inhibitors or gene-silencing approaches—may provide novel avenues for the precision treatment of CKD-associated AF, with significant translational potential.

## Limitations

This study has several limitations that warrant careful consideration. First, the dynamic expression patterns of USP38 across various stages of CKD and its time-dependent effects on AF susceptibility remain incompletely characterized, necessitating longitudinal studies to delineate critical therapeutic windows. Second, although our findings in murine models provide valuable mechanistic insights, their translational relevance is limited by interspecies differences in atrial anatomy (e.g., pulmonary vein trigger distribution) and calcium handling, underscoring the need for validation using human primary atrial cardiomyocytes, patient-derived tissues, or organoid models. Third, the potential involvement of non-canonical pathways—such as direct modulation of TGF-β receptors or mitochondrial oxidative stress—in USP38-mediated fibrogenesis remains unexplored, leaving unanswered questions about the full spectrum of USP38’s pathological roles. Fourth, although our STRAP rescue experiments confirmed the feasibility of downstream pathway modulation, the temporal efficacy of interventions targeting USP38 or STRAP—particularly the therapeutic window for reversing established remodeling—remains undefined. Fifth, the use of AAV9 vectors driven by the ANF promoter, while providing cardiomyocyte-enriched expression, may also lead to off-target effects in non-ventricular or non-myocyte cardiac tissues, and future studies employing more selective promoters or cell-type-specific delivery systems are warranted. Sixth, we cannot exclude the possibility that other molecular mediators besides STRAP may contribute to USP38-associated fibrotic or electrophysiological remodeling; unbiased screening approaches such as proteomics or CRISPR-based modifier screens could provide further insight. Seventh, the lack of a USP38-specific inhibitor currently limits the clinical translatability of our findings; future efforts to develop or identify such compounds would be essential for therapeutic application. Finally, given the absence of significant differences in AERP and baseline ECG parameters among groups, we did not further investigate calmodulin-related pathways in electrical remodeling. Future studies should explore these mechanisms. Collectively, these limitations underscore the need for multi-omics temporal profiling, human translational models, and refined gene delivery tools to deepen mechanistic understanding and inform targeted therapeutic strategies.

## Conclusion

In summary, our findings demonstrate that USP38 enhances AF susceptibility in CKD by stabilizing STRAP and activating the TGF-β1/SMAD pathway, thereby inducing both atrial structural and electrical remodeling. These insights highlight USP38 as a promising therapeutic target for CKD-associated AF.

## Supplementary Information

Supplementary Material 1.

Supplementary Material 2.

## Data Availability

The data that support the findings of this study are available on request from the corresponding author upon reasonable request.

## References

[CR1] Andelova K, Egan Benova T, SzeiffovaBacova B, Sykora M, Prado NJ, Diez ER, et al. Cardiac connexin-43 hemichannels and pannexin1 channels: provocative antiarrhythmic targets. Int J Mol Sci. 2020;22:260. 33383853 10.3390/ijms22010260 PMC7795512

[CR2] Aronson D, Perlow D, Abadi S, Lessick J. Left atrial functional impairment as a predictor of atrial fibrillation: insights from cardiac CT. Eur Radiol. 2025. 10.1007/s00330-025-11348-z. 10.1007/s00330-025-11348-z PMC12166001 39838087

[CR3] Banerjee A, Fauchier L, Vourc’h P, Andres CR, Taillandier S, Halimi JM, et al. A prospective study of estimated glomerular filtration rate and outcomes in patients with atrial fibrillation: the Loire Valley Atrial Fibrillation Project. CHEST. 2014;145:1370–82. 24356875 10.1378/chest.13-2103

[CR4] Bansal N, Fan D, Hsu C, Ordonez JD, Marcus GM, Go AS. Incident atrial fibrillation and risk of end-stage renal disease in adults with chronic kidney disease. Circulation. 2013;127:569–74. 23275377 10.1161/CIRCULATIONAHA.112.123992 PMC3676734

[CR5] Bansal N, Xie D, Tao K, Chen J, Deo R, Horwitz E, et al. Atrial fibrillation and risk of ESRD in adults with CKD. Clin J Am Soc Nephrol. 2016;11:1189–96. 27073197 10.2215/CJN.10921015 PMC4934846

[CR6] Böhm M, Ezekowitz MD, Connolly SJ, Eikelboom JW, Hohnloser SH, Reilly PA, et al. Changes in renal function in patients with atrial fibrillation: an analysis from the RE-LY trial. J Am Coll Cardiol. 2015;65:2481–93. 26065986 10.1016/j.jacc.2015.03.577

[CR7] Chen TH, Chu YC, Ou SM, Tarng DC. Associations of atrial fibrillation with renal function decline in patients with chronic kidney disease. Heart. 2022;108:438–44. 34193464 10.1136/heartjnl-2021-319297

[CR8] Chugh SS, Havmoeller R, Narayanan K, Singh D, Rienstra M, Benjamin EJ, et al. Worldwide epidemiology of atrial fibrillation: a Global Burden of Disease 2010 study. Circulation. 2014;129:837–47. 24345399 10.1161/CIRCULATIONAHA.113.005119 PMC4151302

[CR9] Chyou JY, Tay WT, Tromp J, Ouwerkerk W, Yiu KH, Cleland JGF, et al. Prognostic implications and global perspectives of atrial fibrillation in patients hospitalized for heart failure. JACC Heart Fail. 2025;S2213–1779(24):00872–2. 10.1016/j.jchf.2024.11.009 39918534

[CR10] Fauchier L, Bisson A, Clementy N, Vour’ch P, Angoulvant D, Babuty D, et al. Changes in glomerular filtration rate and outcomes in patients with atrial fibrillation. Am Heart J. 2018;198:39–45. 29653646 10.1016/j.ahj.2017.12.017

[CR11] Gao P, Xie B, Zhou Z, Tse G, Liu T. Elevation of circulating FGF23 in chronic kidney disease promotes atrial fibrosis through the AKT pathway. Eur Heart J. 2024;45 Supplement_1:ehae666.443.

[CR12] Genovesi S, Vincenti A, Rossi E, Pogliani D, Acquistapace I, Stella A, et al. Atrial fibrillation and morbidity and mortality in a cohort of long-term hemodialysis patients. Am J Kidney Dis. 2008;51:255–62. 18215703 10.1053/j.ajkd.2007.10.034

[CR13] Genovesi S, Camm AJ, Covic A, Burlacu A, Meijers B, Franssen C, et al. Treatment strategies of the thromboembolic risk in kidney failure patients with atrial fibrillation. Nephrol Dial Transplant. 2024;39:1248–57. 38816212 10.1093/ndt/gfae121 PMC11288792

[CR14] Hart RG, Eikelboom JW, Brimble KS, McMurtry MS, Ingram AJ. Stroke prevention in atrial fibrillation patients with chronic kidney disease. Can J Cardiol. 2013;29:S71-8. 23790601 10.1016/j.cjca.2013.04.005

[CR15] Hawkins NM, Jhund PS, Pozzi A, O’Meara E, Solomon SD, Granger CB, et al. Severity of renal impairment in patients with heart failure and atrial fibrillation: implications for non-vitamin K antagonist oral anticoagulant dose adjustment. Eur J Heart Fail. 2016;18:1162–71. 27594177 10.1002/ejhf.614

[CR16] Hohl M, Selejan SR, Wintrich J, Lehnert U, Speer T, Schneider C, et al. Renal denervation prevents atrial arrhythmogenic substrate development in CKD. Circ Res. 2022;130:814–28. 35130718 10.1161/CIRCRESAHA.121.320104

[CR17] Hsu YJ, Chang GJ, Lai YJ, Chan YH, Chen WJ, Kuo CT, et al. High-phosphate diet causes atrial remodeling and increases atrial fibrillation vulnerability via STAT3/NF-κB signaling and oxidative stress. Acta Physiol (Oxf). 2023;238:e13964. 36929808 10.1111/apha.13964

[CR18] Jakob D, Klesen A, Darkow E, Kari FA, Beyersdorf F, Kohl P, et al. Heterogeneity and remodeling of ion currents in cultured right atrial fibroblasts from patients with sinus rhythm or atrial fibrillation. Front Physiol. 2021;12:673891. 34149453 10.3389/fphys.2021.673891 PMC8209389

[CR19] Lai YJ, Tsai FC, Chang GJ, Chang SH, Huang CC, Chen WJ, et al. miR-181b targets semaphorin 3A to mediate TGF-β-induced endothelial-mesenchymal transition related to atrial fibrillation. J Clin Invest. 2022;132:e142548. 35775491 10.1172/JCI142548 PMC9246393

[CR20] Lekawanvijit S, Adrahtas A, Kelly DJ, Kompa AR, Wang BH, Krum H. Does indoxyl sulfate, a uraemic toxin, have direct effects on cardiac fibroblasts and myocytes? Eur Heart J. 2010;31:1771–9. 20047993 10.1093/eurheartj/ehp574

[CR21] Li X, Cui X, Zhou S, Xing DL, Piao HR, Zhang QG, et al. The novel ginsenoside AD2 prevents angiotensin II-induced connexin 40 and connexin 43 dysregulation by activating AMP kinase signaling in perfused beating rat atria. Chem Biol Interact. 2021;339:109430. 33676887 10.1016/j.cbi.2021.109430

[CR22] Lin JC, Kuo WW, Baskaran R, Chen MC, Ho TJ, Chen RJ, et al. Enhancement of beta-catenin in cardiomyocytes suppresses survival protein expression but promotes apoptosis and fibrosis. Cardiol J. 2017;24:195–205. 27734460 10.5603/CJ.a2016.0087

[CR23] Pan Y, Xiao Z, Yang H, Kong B, Meng H, Shuai W, et al. USP38 exacerbates pressure overload-induced left ventricular electrical remodeling. Mol Med. 2024;30:97. 38937697 10.1186/s10020-024-00846-3 PMC11210128

[CR24] Park S, Lee S, Kim Y, Lee Y, Kang MW, Han K, et al. Reduced risk for chronic kidney disease after recovery from metabolic syndrome: a nationwide population-based study. Kidney Res Clin Pract. 2020;39:180–91. 32344501 10.23876/j.krcp.20.016 PMC7321670

[CR25] Potter JA, Price GW, Cliff CL, Green CR, Squires PE, Hills CE. Collagen I modifies connexin-43 hemichannel activity via integrin α2β1 binding in TGFβ1-evoked renal tubular epithelial cells. Int J Mol Sci. 2021;22:3644. 33807408 10.3390/ijms22073644 PMC8038016

[CR26] Price GW, Chadjichristos CE, Kavvadas P, Tang SCW, Yiu WH, Green CR, et al. Blocking Connexin-43 mediated hemichannel activity protects against early tubular injury in experimental chronic kidney disease. Cell Commun Signal. 2020;18:79. 32450899 10.1186/s12964-020-00558-1 PMC7249671

[CR27] Qiu H, Ji C, Liu W, Wu Y, Lu Z, Lin Q, et al. Chronic kidney disease increases atrial fibrillation inducibility: involvement of inflammation, atrial fibrosis, and connexins. Front Physiol. 2018;9:1726. 30564139 10.3389/fphys.2018.01726 PMC6288485

[CR28] Saljic A, Grandi E, Dobrev D. TGF-β1-induced endothelial-mesenchymal transition: a potential contributor to fibrotic remodeling in atrial fibrillation? J Clin Invest. 2022;132:e161070. 35775488 10.1172/JCI161070 PMC9246376

[CR29] Shen WC, Chou YH, Shi LS, Chen ZW, Tu HJ, Lin XY, et al. AST-120 improves cardiac dysfunction in acute kidney injury mice via suppression of apoptosis and proinflammatory NF-κB/ICAM-1 signaling. J Inflamm Res. 2021;14:505–18. 33658826 10.2147/JIR.S283378 PMC7917393

[CR30] Shi S, Tang Y, Zhao Q, Yan H, Yu B, Zheng Q, et al. Prevalence and risk of atrial fibrillation in China: a national cross-sectional epidemiological study. Lancet Reg Health West Pac. 2022;23:100439. 10.1016/j.lanwpc.2022.100439 PMC9252928 35800039

[CR31] Sidaway P. Chronic kidney disease: targeting connexin-43 reduces progression of CKD in mice. Nat Rev Nephrol. 2014;10:424. 24914584 10.1038/nrneph.2014.101

[CR32] Sinha F, Schweda F, Maier LS, Wagner S. Impact of impaired kidney function on arrhythmia-promoting cardiac ion channel regulation. Int J Mol Sci. 2023;24:14198. 37762501 10.3390/ijms241814198 PMC10532292

[CR33] Soliman EZ, Prineas RJ, Go AS, Xie D, Lash JP, Rahman M, et al. Chronic kidney disease and prevalent atrial fibrillation: the Chronic Renal Insufficiency Cohort (CRIC). Am Heart J. 2010;159:1102–7. 20569726 10.1016/j.ahj.2010.03.027 PMC2891979

[CR34] Song J, Navarro-Garcia JA, Wu J, Saljic A, Abu-Taha I, Li L, et al. Chronic kidney disease promotes atrial fibrillation via inflammasome pathway activation. J Clin Invest. 2023;133:e167517. 37581942 10.1172/JCI167517 PMC10541185

[CR35] Sun CY, Chang SC, Wu MS. Uremic toxins induce kidney fibrosis by activating intrarenal renin–angiotensin–aldosterone system associated epithelial-to-mesenchymal transition. PLoS One. 2012;7:e34026. 22479508 10.1371/journal.pone.0034026 PMC3316590

[CR36] Tsai LT, Weng TI, Chang TY, Lan KC, Chiang CK, Liu SH. Inhibition of indoxyl sulfate-induced reactive oxygen species-related ferroptosis alleviates renal cell injury in vitro and chronic kidney disease progression in vivo. Antioxidants (Basel). 2023;12:1931. 38001784 10.3390/antiox12111931 PMC10669521

[CR37] Turakhia MP, Blankestijn PJ, Carrero JJ, Clase CM, Deo R, Herzog CA, et al. Chronic kidney disease and arrhythmias: conclusions from a kidney disease: improving global outcomes (KDIGO) controversies conference. Eur Heart J. 2018;39:2314–25. 29522134 10.1093/eurheartj/ehy060 PMC6012907

[CR38] Wang Y, Gu YH, Ren KW, Xie X, Wang SH, Zhu XX, et al. Administration of USP7 inhibitor p22077 alleviates Angiotensin II (Ang II)-induced atrial fibrillation in mice. Hypertens Res. 2024;47:1309–22. 38374239 10.1038/s41440-024-01581-2

[CR39] Watanabe H, Tanabe N, Watanabe T, Darbar D, Roden DM, Sasaki S, et al. Metabolic syndrome and risk of development of atrial fibrillation: the Niigata preventive medicine study. Circulation. 2008;117:1255–60. 18285562 10.1161/CIRCULATIONAHA.107.744466 PMC2637133

[CR40] Xiao Z, Dai C, Yu T, Zhu J, Pan Y, Shuai W, et al. Ubiquitin specific protease 38 aggravates pathological cardiac remodeling by stabilizing phospho-TBK1. Int J Biol Sci. 2024c;20:1815–32. 38481817 10.7150/ijbs.85562 PMC10929191

[CR41] Xiao Z, Pan Y, Kong B, Meng H, Shuai W, Huang H. Ubiquitin-specific protease 38 promotes inflammatory atrial fibrillation induced by pressure overload. Europace. 2024a;26:euad366. 10.1093/europace/euad366 PMC10823351 38288617

[CR42] Xiao Z, Yang H, Pan Y, Meng H, Qu Z, Kong B, et al. Ubiquitin-specific protease 38 promotes atrial fibrillation in diabetic mice by stabilizing iron regulatory protein 2. Free Radic Biol Med. 2024b;224:88–102. 39173894 10.1016/j.freeradbiomed.2024.08.021

[CR43] Xie SY, Liu SQ, Zhang T, Shi WK, Xing Y, Fang WX, et al. USP28 serves as a key suppressor of mitochondrial morphofunctional defects and cardiac dysfunction in the diabetic heart. Circulation. 2024;149:684–706. 37994595 10.1161/CIRCULATIONAHA.123.065603

[CR44] Zhang Z, Wu W, Jiao H, Chen Y, Ji X, Cao J, et al. Squalene epoxidase promotes hepatocellular carcinoma development by activating STRAP transcription and TGF-β/SMAD signalling. Br J Pharmacol. 2023;180:1562–81. 36581319 10.1111/bph.16024

[CR45] Zhu J, Luo Z, Pan Y, Zheng W, Li W, Zhang Z, et al. H19/miR-148a/USP4 axis facilitates liver fibrosis by enhancing TGF-β signaling in both hepatic stellate cells and hepatocytes. J Cell Physil. 2019;234:9698–710. 10.1002/jcp.27656 30362572

[CR46] Zimmerman D, Sood MM, Rigatto C, Holden RM, Hiremath S, Clase CM. Systematic review and meta-analysis of incidence, prevalence and outcomes of atrial fibrillation in patients on dialysis. Nephrol Dial Transplant. 2012;27:3816–22. 23114904 10.1093/ndt/gfs416

