# Supplementary material for: Ubiquitin-specific protease 38 modulates atrial fibrillation susceptibility in chronic kidney disease via STRAP stabilization and activation of TGF-β/SMAD signaling
Source: Mol Med. 2025 Jun 13;31:238. doi: 10.1186/s10020-025-01296-1 (PMC12164109; doi:10.1186/s10020-025-01296-1)
Supplement: Supplementary file 1 — Supplementary Material 1. [file 10020_2025_1296_MOESM1_ESM.docx]

**Supplementary material**

**Supplementary methods**

**1 Construction of Cardiac-Specific USP38 Knockout (USP38-CKO) Mice**

The USP38-CKO mice were constructed by Cyagen Biosciences Inc. The brief procedure is as follows: The sgRNA, donor vector containing loxP sites, and Cas9 mRNA that were synthesized in vitro were co-injected into mouse fertilized eggs, which were subsequently transplanted into pseudopregnant female mice. Genotypes were identified using the following primers:

USP38 forward primer: 5’-ATGATCGGAGGTTTCCTTGTGTTG-3’

USP38 reverse primer: 5’-TCTGATGTCTGAGTATCAACGAAGA-3’.

The USP38-CKO genotype was maintained by crossing USP38flox/flox mice with α-MHC-Cre tool mice. In the experiments, USP38-CKO and USP38flox/flox mice were ultimately selected.

**2 Construction of Cardiac-Specific USP38 Transgenic (USP38-TG) Mice**

The sgRNA targeting the mouse ROSA26 gene, the vector containing the "Kozak-mouse USP38 CDS" sequence, and Cas9 mRNA were injected into mouse fertilized eggs to generate conditional gene knock-in progeny. The mouse genotypes were identified using the following primers:

USP38 forward primer F4: 5’-ATCTGCTTCCTGTTCGTTCCGAC-3’

Reverse primer R4: 5’-CTTTATTAGCCAGAAGTCAG ATGC-3’.

The mice were crossed with α-MHC-Cre tool mice to maintain the USP38 overexpression genotype.

**3 Detection of Serum Indicators, Organ Weights, and Tissue Analysis**

Four weeks after 5/6 nephrectomy (5/6Nx), mice were anesthetized with pentobarbital (100 mg/kg) via intraperitoneal injection and sacrificed. Body weight was promptly measured, and blood was promptly collected for the detection of serum urea nitrogen and creatinine. The left atrial tissue was fixed with 4% paraformaldehyde, embedded in paraffin, sectioned to a thickness of 3 μm, and subjected to Masson staining for the detection of collagen deposition. The proportion of fibrotic area (fibrotic tissue area / normal myocardial tissue area) was calculated using Image J software.

**4 Echocardiography**

Cardiac systolic and diastolic functions were evaluated using transthoracic echocardiography (Vinno Technology, China). The left ventricular fractional shortening (LVFS) and left ventricular ejection fraction (LVEF) were measured by left ventricular short-axis M-mode; diastolic function was evaluated by the four-chamber view, and the E peak (early filling) and A peak (atrial filling) of the mitral valve blood flow spectrum were measured in the pulse Doppler mode, and the E/A ratio was calculated. The echocardiographic detection and data analysis were conducted by blinded technicians.

**5 Electrocardiogram Analysis**

Mice were anesthetized by inhalation of 1.5 - 2% isoflurane, and electrodes were implanted subcutaneously to record the simulated surface lead electrocardiogram (II lead), and the P wave duration, PR interval, QRS duration, RR interval, and corrected QT interval (corrected using the Bazett formula) were measured. Signal processing and analysis were performed using LabChart 8 software (AD Instruments).

**6 In Vivo Electrophysiological Studies**

After anesthesia with 1% pentobarbital sodium, the chest was opened to expose the heart. For the recording of left ventricular monophasic action potential (MAP), platinum electrodes and stimulation protocols were used: The MAP electrode was placed on the anterior free wall of the left ventricle to record the epicardial MAP, and a pair of platinum stimulation electrodes were placed at the base of the right ventricle. The electrical stimulation protocol referred to previous literature. The heart was stimulated with a fixed pacing cycle length (PCL), and atrial fibrillation (AF) was induced by short bursts of rapid electrical stimulation (5 V, 50 Hz, 2 ms pulses, lasting for 2 seconds); if AF persisted for more than 2 seconds, it was determined as successful induction of atrial fibrillation.

**7 Immunofluorescence Staining**

The expression of α-SMA, Cx40, and Cx43 in atrial tissues was detected by immunofluorescence: After fixation of frozen heart sections, they were incubated with α-SMA primary antibody (AF1032, Affinity, China), Cx40 primary antibody (A7231, Abclonal, China), and Cx43 primary antibody (A23120, Abclonal, China), followed by the addition of secondary antibodies. The nuclei were labeled with DAPI staining solution (G1012-100ML, Servicebio, China), and the fluorescence images were captured by a fluorescence microscope (Nikon Eclipse C1, Japan).

**8 Cell Culture**

HL‐1 cells, a murine atrial myocyte line, were maintained in Claycomb medium supplemented with 10% fetal bovine serum (10099, Gibco, Australia), 100 µM epinephrine, and 4 mM L‐glutamine at 37 °C in a humidified atmosphere containing 5% CO2. Cells were transduced with adenoviral constructs—namely, a control short hairpin RNA (AdshRNA), a USP38‐targeting short hairpin RNA (AdshUSP38), an adenovirus expressing green fluorescent protein (AdGFP), and an adenovirus driving USP38 overexpression (AdUSP38)—with the efficiency of knockdown or overexpression subsequently verified by Western blot analysis. To recapitulate a hypertrophic state in vitro, the cells were then treated with 100 µM indoxyl sulfate for 48 hours.

**9 Co-Immunoprecipitation (Co-IP)**

HEK-293T cells were co-transfected with the designated plasmids, and co-immunoprecipitation (Co-IP) experiments were conducted. Then, the cells were lysed with IP lysis buffer (G2038-100ML, Servicebio, China), and PMSF (G2008-1ML) and protease inhibitors (G2006-250UL) were added. The cell lysate was ice-incubated for 20 minutes, and the supernatant containing total protein was collected after centrifugation and incubated with anti-USP38, anti-STRAP, or anti-IgG antibodies at 4°C overnight. Subsequently, magnetic beads (L-1004, Biolinkedlin, China) were placed in the supernatant, and the mixture was rotated at room temperature for 3 hours, and boiled in loading buffer for 10 minutes. The immunoprecipitated products were detected by Western blot, and the experiment was repeated three times to evaluate exogenous co-immunoprecipitation.

Mouse atrial tissues were lysed with IP lysis buffer containing PMSF and cocktail. The supernatant was collected after centrifugation, and immunocomplexes were formed by adding anti-USP38 (17767-1-AP, Proteintech, China), anti-STRAP (18277-1-AP, Proteintech, China), or IgG (8726, CTS, USA) antibodies and incubating at 4°C overnight. The complexes were incubated with magnetic beads (L-1004, Biolinkedlin, China) at room temperature, washed, and eluted by boiling. The immunoprecipitated products were detected by Western blot, and the experiment was repeated three times to evaluate endogenous co-immunoprecipitation.

**10 Ubiquitination Assay**

Four weeks after 5/6Nx, mice were anesthetized with pentobarbital (100mg/kg) by intraperitoneal injection and sacrificed, and the lysates of mouse atrial tissues were collected. Anti-STRAP antibody (incubated at 4°C overnight) was added, followed by incubation with magnetic beads for 3 hours, and finally, the ubiquitination level of STRAP was detected by Western blot.

**11 Quantitative Real-Time PCR (qRT-PCR)**

RNA was isolated from the left ventricular tissues using Invitrogen reagents and reverse transcribed into cDNA using the PrimeScript RT kit from TaKaRa. Quantitative real-time PCR was performed using the Applied Biosystems VII7 system (Life Technologies, Carlsbad, USA) to quantify the mRNA levels of the target proteins. The primer sequences used in this study are presented in **Supplementary Table S1**.

**12 Western Blot Analysis**

Left ventricular tissues from each experimental group were randomly selected for Western blot analysis. After determining and normalizing the total protein concentration using the BCA Protein Assay Kit (AS1086, Osborne Bio, China), 40 μg of protein was separated by SDS-polyacrylamide gel electrophoresis and transferred to a nitrocellulose membrane (NC membrane). The NC membrane was incubated overnight at 4°C with the corresponding primary antibodies (**Supplementary Table S2**), followed by incubation with the corresponding secondary antibodies at room temperature for 2 hours. Finally, the signals were collected using a chemiluminescence imaging system. The obtained data were normalized to the internal reference GAPDH for statistical analysis.

**Supplementary Table S1. Primer for qRT-PCR.**

| Primer | Species | Sequence (5' to 3') |
| --- | --- | --- |
| USP38-F | Mouse | TCATCAGGAGCCTAACCACC |
| USP38-R | Mouse | TCAGGAGAGCAATTACCCACG |

**Supplementary Table S2. Primary antibodies for Western blots.**

| primary antibodies | Source organism | producer | Number |
| --- | --- | --- | --- |
| USP38 | Rabbit | Proteintech | 17767-1-AP |
| Cx43 | Rabbit | Abclonal | A23120 |
| Cx40 | Rabbit | Abclonal | A7231 |
| α-SMA | Rabbit | Affinity | AF1032 |
| Collagen I | Rabbit | Affinity | AF7001 |
| Collagen III | Rabbit | Affinity | AF5457 |
| RyR2 | Rabbit | Affinity | AF0015 |
| p-RyR2 | Rabbit | Affinity | AF7475 |
| SERCA2a | Rabbit | Affinity | DF6240 |
| PLB | Rabbit | Affinity | AF7778 |
| p-PLB | Rabbit | CST | #8496 |
| Ubiquitin | Rabbit | Proteintech | 10201-2-AP |
| TGF-β1 | Rabbit | Proteintech | 26155-1-AP |
| SMAD2 | Rabbit | Proteintech | 12570-1-AP |
| p-SMAD2 | Rabbit | Affinity | AF3367 |
| SMAD3 | Rabbit | Proteintech | 66516-1-Ig |
| p-SMAD3 | Rabbit | Affinity | AF3362 |
| GAPDH | Rabbit | Proteintech | 60004-1-Ig |
| STRAP | Rabbit | Proteintech | 18277-1-AP |
| STRAP | Mouse | Proteintech | 66712-1-Ig |
| IgG | Rabbit | CST | 8726 |
| Flag | Rabbit | Abclonal | AE169 |
| Myc | Rabbit | Abclonal | AE070 |


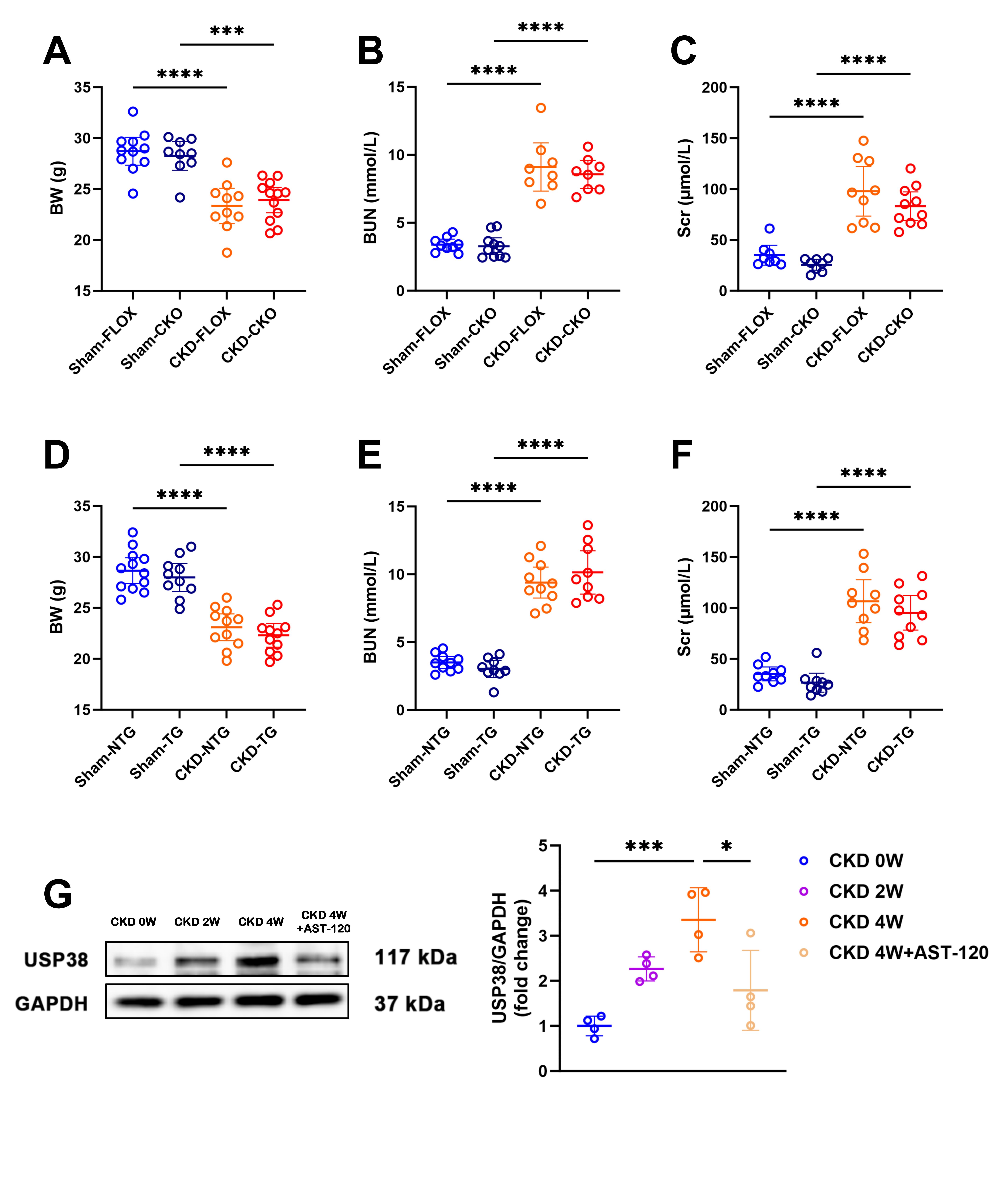


**Supplementary Figure S1. The 5/6Nx procedure significantly exacerbated renal dysfunction in the mice.** A-C. Statistical analysis of body weight, urea nitrogen and serum creatinine in USP38-FLOX and USP38-CKO mice at 4 weeks after sham or 5/6Nx surgery (n =10). D-F. Statistical analysis of body weight, urea nitrogen and serum creatinine in USP38-NTG and USP38-TG mice at 4 weeks after sham or 5/6Nx surgery (n =10-12). G. Representative Western blot (WB) images and density quantification of USP38 protein levels from wild type mice 2 and 4 weeks after 5/6nx and 4 weeks after 5/6nx plus gavage of AST-120 for 4 weeks (n = 4). (* p<0.05, *** p <0.001, **** p <0.0001)


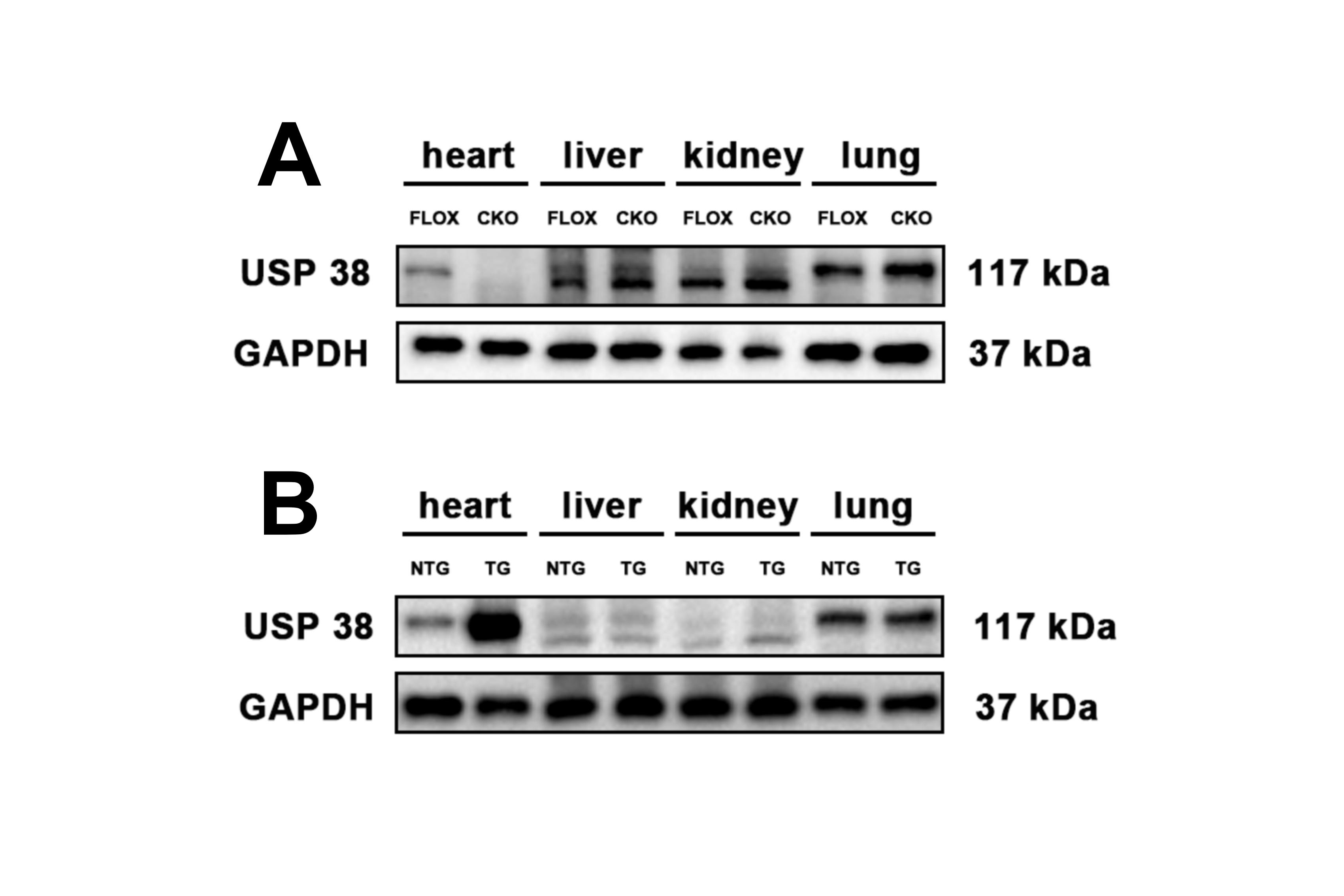


**Supplementary Figure S2. Validation after heart-specific editing of USP38.** A. Representative image of western blotting of USP38 protein level expression in heart, liver, kidney, and lung after cardiac specific knockout of USP38. B. Representative image of western blotting of USP38 protein level expression in heart, liver, kidney, and lung after cardiac specific overexpression of USP38.


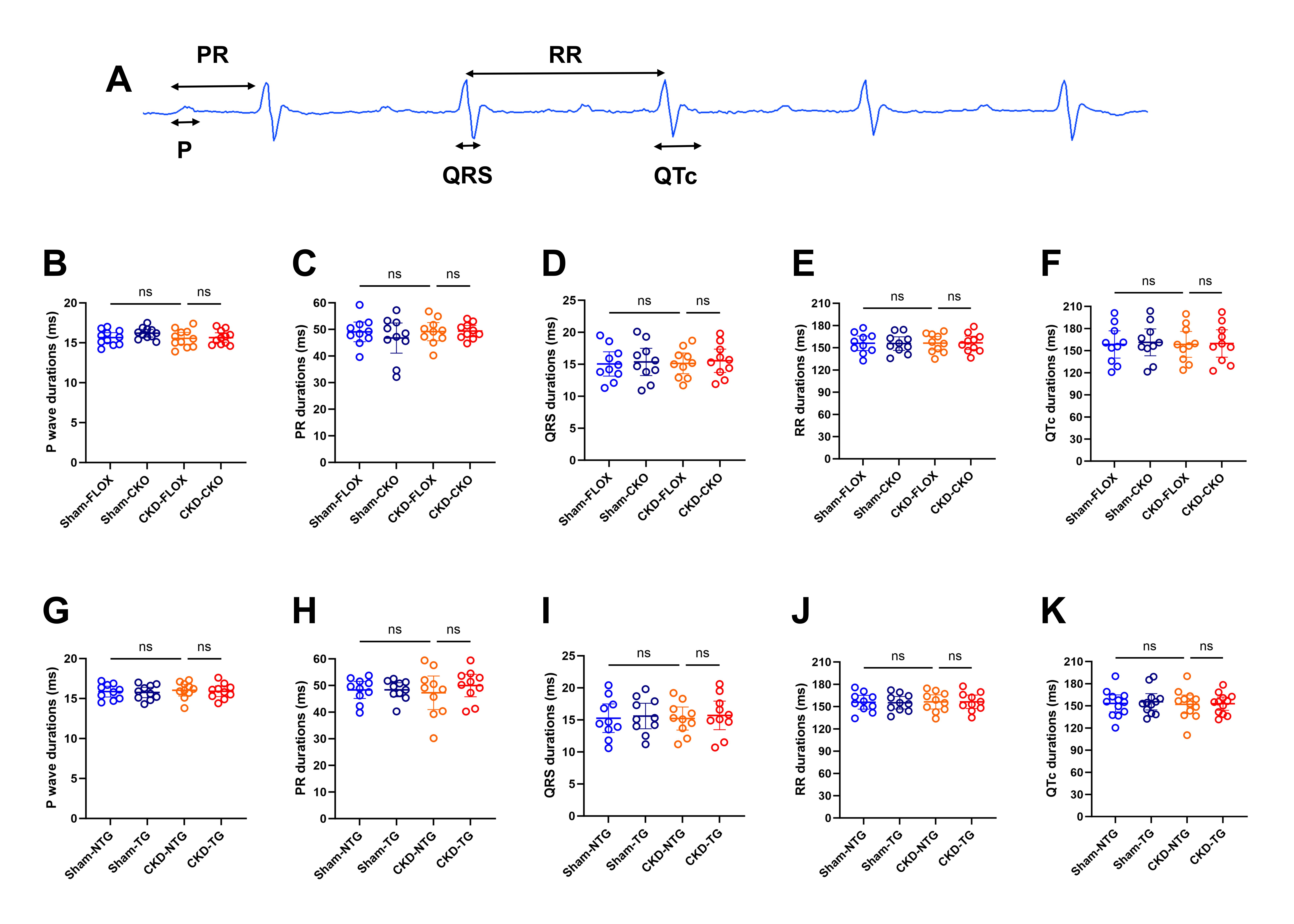


**Supplementary Figure S3. Statistical analysis of body surface electrocardiogram parameters.** A. Representative surface electrocardiogram images. B-F. Statistical analysis of P wave, PR duration, QRS duration, RR duration and QTc duration in cardiogram of cardiac specific USP38 knockout mice (n=10-13). G-K. Statistical analysis of P wave, PR duration, QRS duration, RR duration and QTc duration in cardiogram of mice with cardiac specific USP38 overexpression (n=10-13).

**
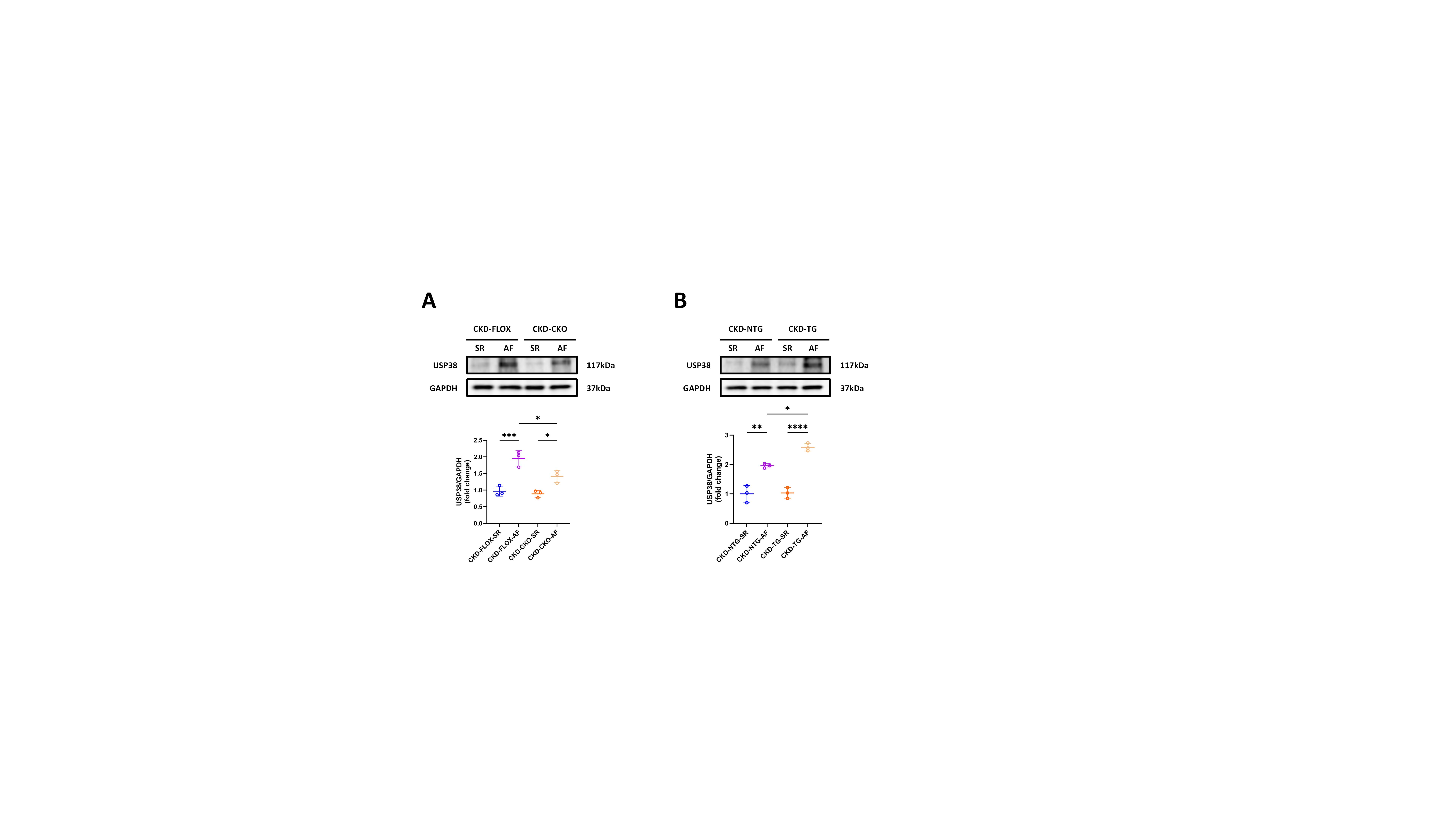
Supplementary Figure S4. USP38 increases the susceptibility to atrial fibrillation in CKD mice.** A. Representative western blotting images of USP38 protein expression in hearts of CKD-FLOX or CKD-CKO mice with AF versus SR (n=3). B. Representative western blotting images of USP38 protein expression in hearts of CKD-NTG or CKD-TG mice with AF versus SR (n=3). (** p <0.01**** p <0.0001)


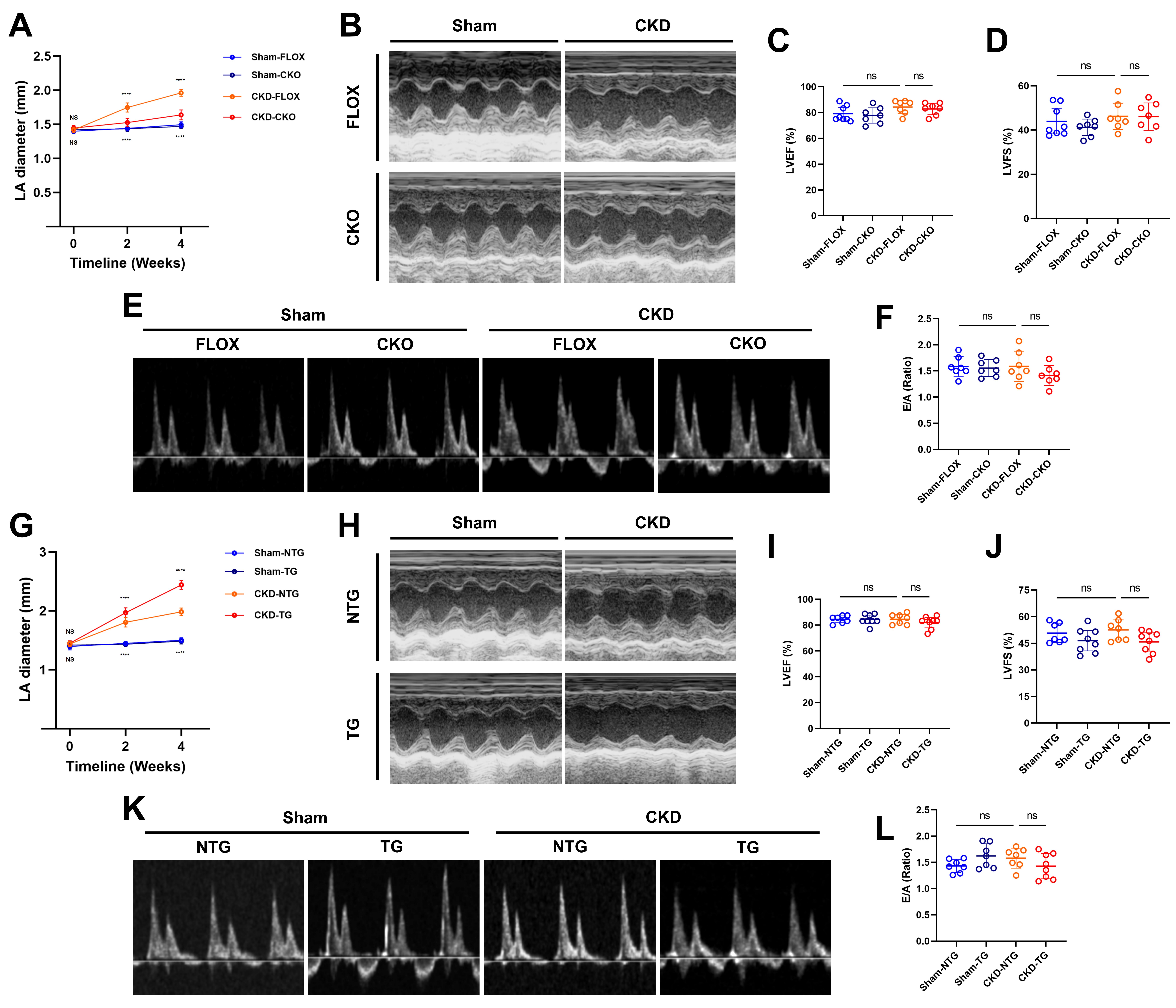


**Supplementary Figure S5. Evaluation of cardiac function in mice.** A. Temporal trends of LA expansion during 4 weeks of CKD regimen in FLOX and CKO mice (n=8). B. Representative short-axis echocardiographic images of FLOX and CKO mice. C, D. Statistical analysis of ventricular systolic function in FLOX and CKO mice (n=6-8). E. Representative echocardiographic spectral Doppler images of FLOX and CKO mice. F. Statistical analysis of ventricular diastolic function in FLOX and CKO mice (n=6-8). G. Temporal trends of LA expansion during 4 weeks of CKD regimen in NTG and TG mice (n=8). H. Representative short-axis echocardiographic images of NTG and TG mice. I, J. Statistical analysis of ventricular systolic function in NTG and TG mice (n=6-8). K. Representative echocardiographic spectral Doppler images of NTG and TG mice. L. Statistical analysis of ventricular diastolic function in NTG and TG mice (n=6-8). (**** p <0.0001)


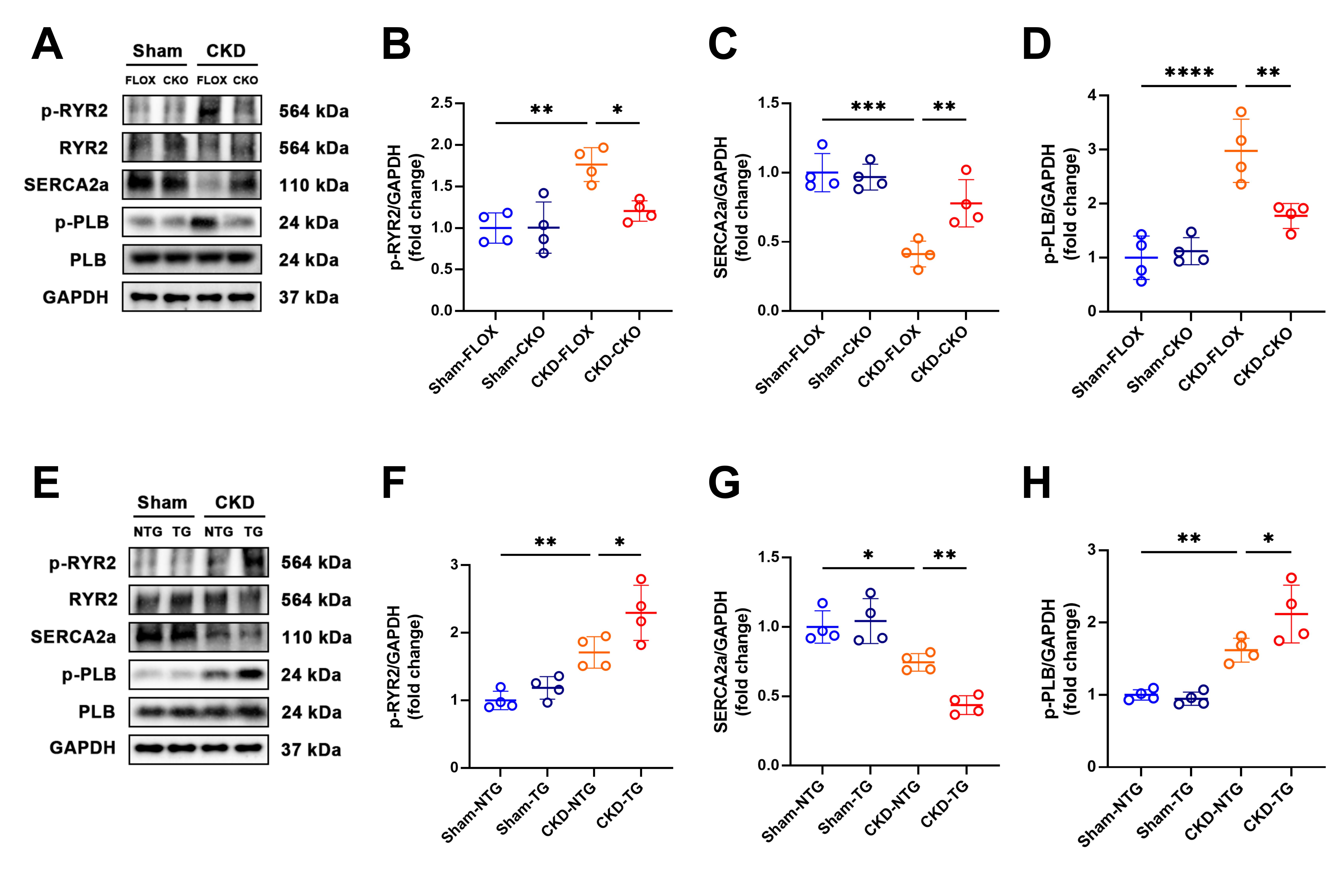
**Supplementary Figure S6. USP38 alters the level of calcium processing protein in atrial tissue.** A-D. Representative western blot images and statistical analysis of SERCA2a, p-RyR2 and p-PLB protein levels in the left atrial tissues of FLOX and CKO mice 4 weeks after sham operation or 5/6Nx operation (n = 4). Representative western blot images and statistical analysis of SERCA2a, p-RyR2 and p-PLB protein levels in the left atrial tissues of NTG and TG mice 4 weeks after sham operation or 5/6Nx operation (n = 4). (* p<0.05, ** p<0.01, *** p <0.001, **** p <0.0001

**
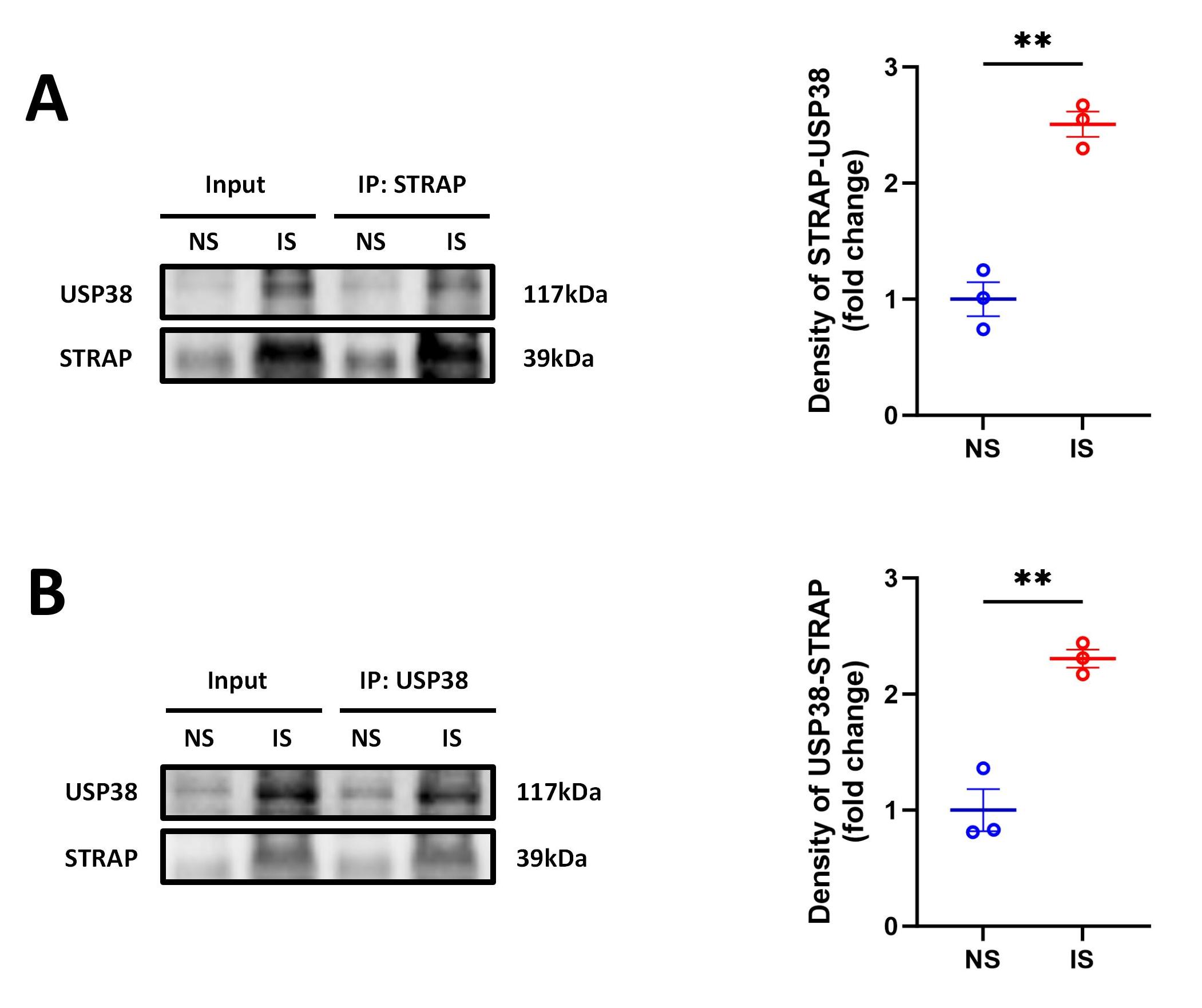
Supplementary Figure S7. USP38 interacts with STRAP in vitro.** A. Lysates from HL-1 cell stimulated with NS or IS. Equal amounts of protein lysates were immunoprecipitated with anti-USP38 antibody and analyzed by western blotting with the indicated antibodies (n = 3). B. Lysates from HL-1 cell stimulated with NS or IS. Equal amounts of protein lysates were immunoprecipitated with anti-STRAP antibody and analyzed by western blotting with the indicated antibodies (n = 3).
