# Supplementary material for: Ubiquitin-specific protease 38 modulates atrial fibrillation susceptibility in chronic kidney disease via STRAP stabilization and activation of TGF-β/SMAD signaling
Source: Mol Med. 2025 Jun 13;31:238. doi: 10.1186/s10020-025-01296-1 (PMC12164109; doi:10.1186/s10020-025-01296-1)
Supplement: Supplementary file 2 — Supplementary Material 2. [file 10020_2025_1296_MOESM2_ESM.docx]

Figure 1C

USP38 GAPDH

| Sham  repeat 1，2，3 | CKD 2w  repeat 1，2，3 | CKD 4w  repeat 1，2，3 |
| --- | --- | --- |


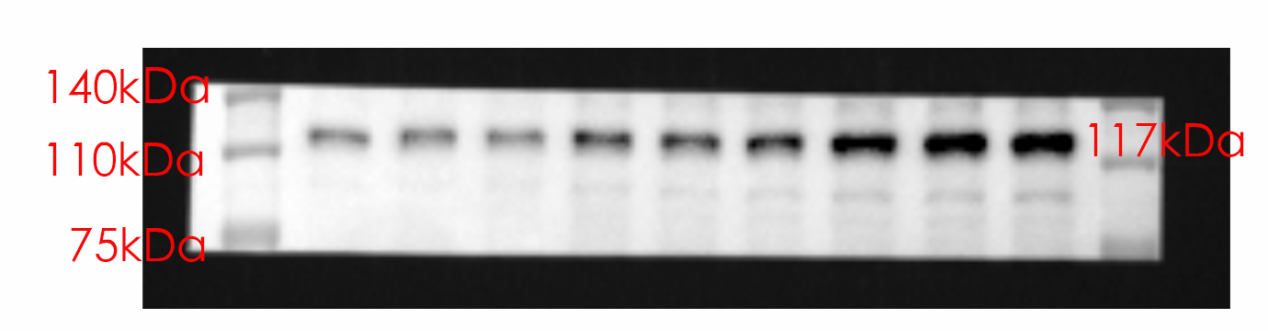


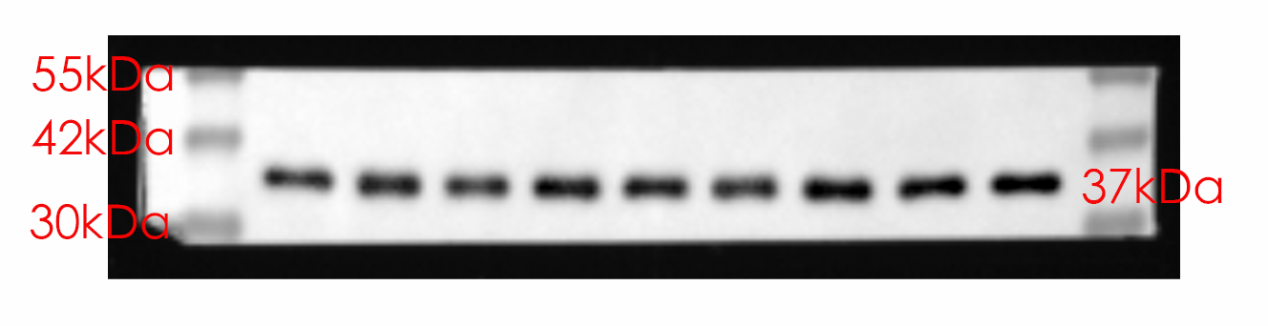


Figure 1D

USP38 GAPDH

| HL-1 NS  repeat 1，2，3 | HL-1 IS 24h  repeat 1，2，3 | HL-1 IS 48h  repeat 1，2，3 |
| --- | --- | --- |


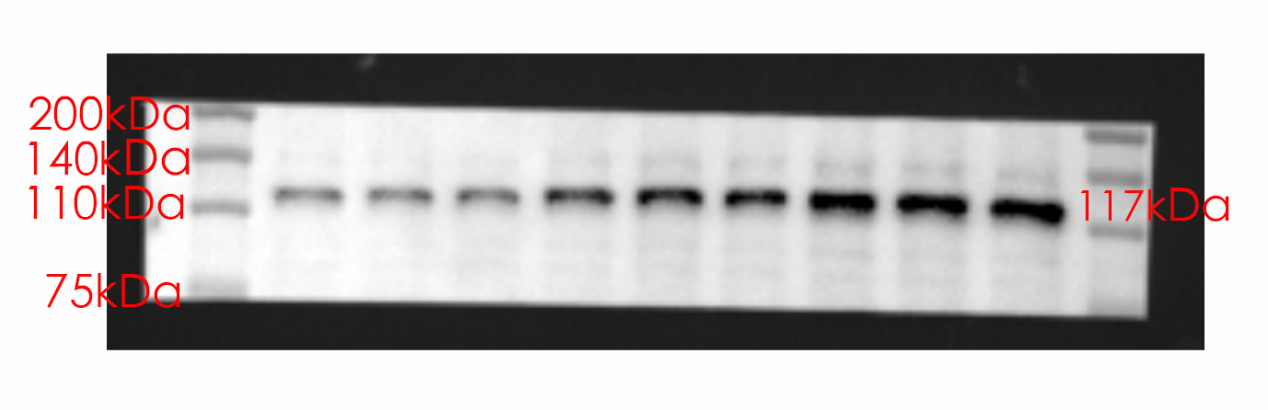


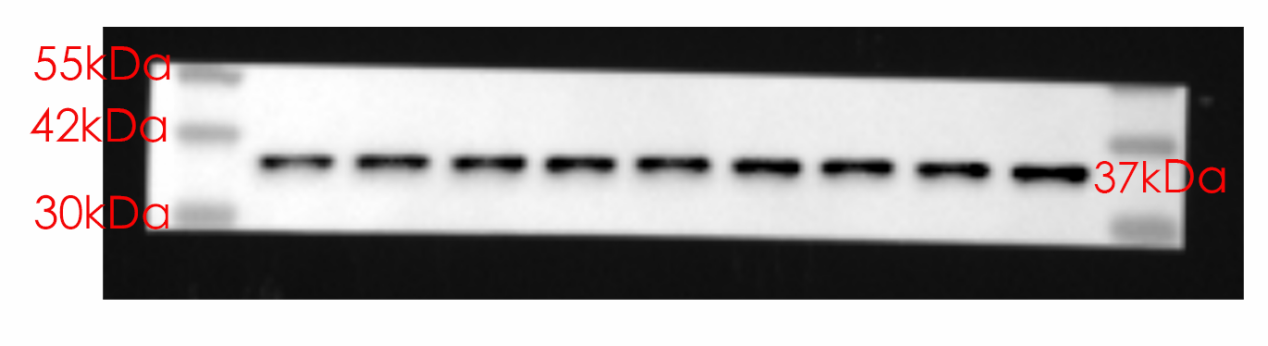


Figure 3F

collagen I GAPDH

| USP38fl/fl Sham | USP38cko Sham | USP38fl/fl CKD | USP38ckol CKD |
| --- | --- | --- | --- |

repeat 1


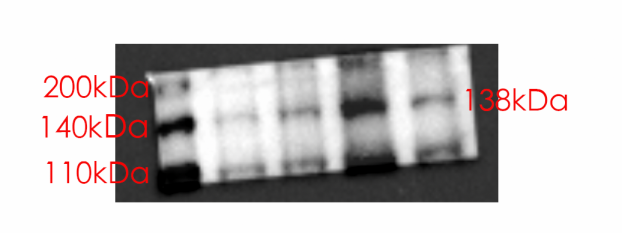

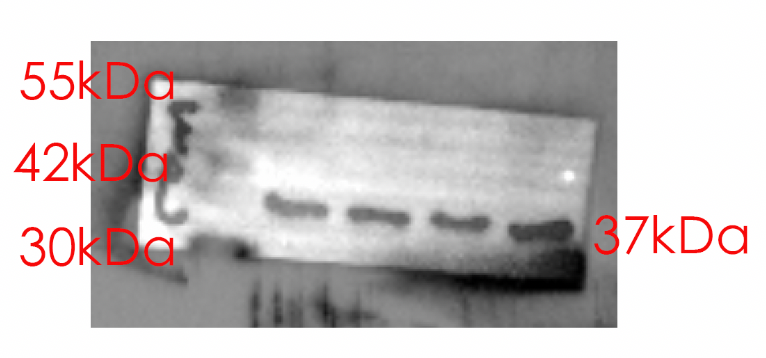


repeat 2


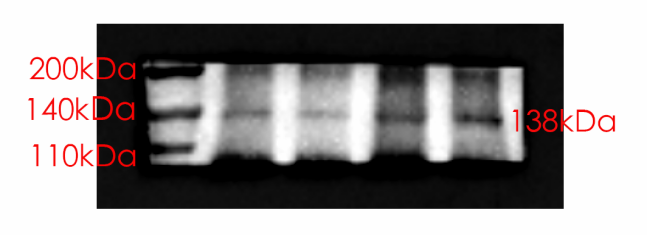

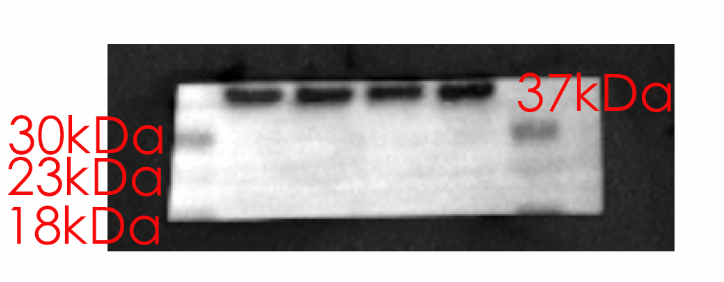


repeat 3


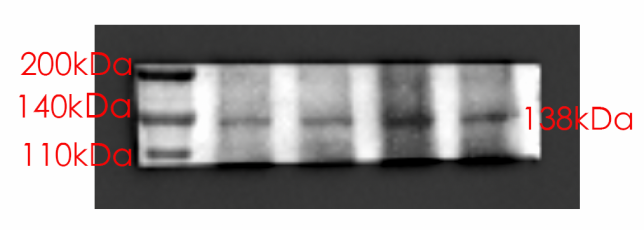

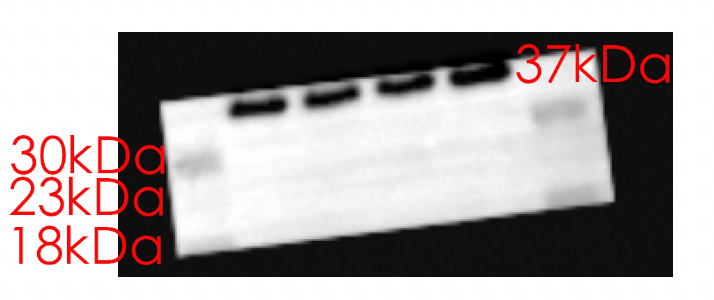


repeat 4


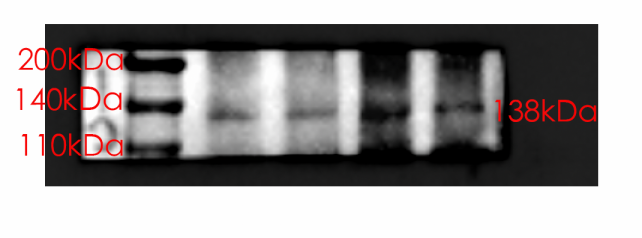

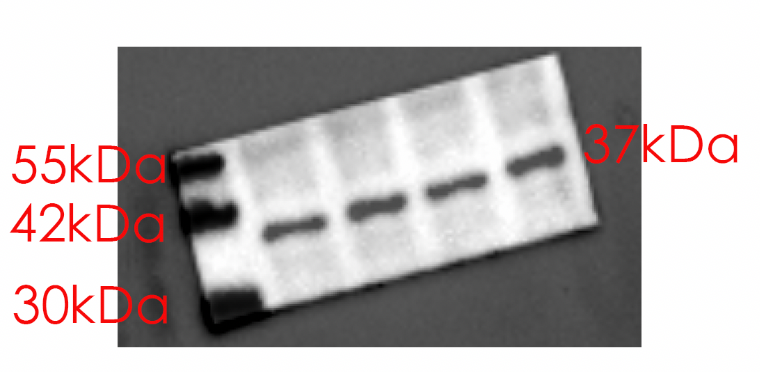


collagen III GAPDH

| USP38fl/fl Sham | USP38cko Sham | USP38fl/fl CKD | USP38ckol CKD |
| --- | --- | --- | --- |

repeat 1


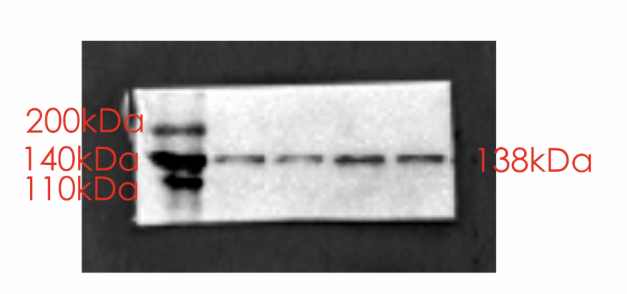

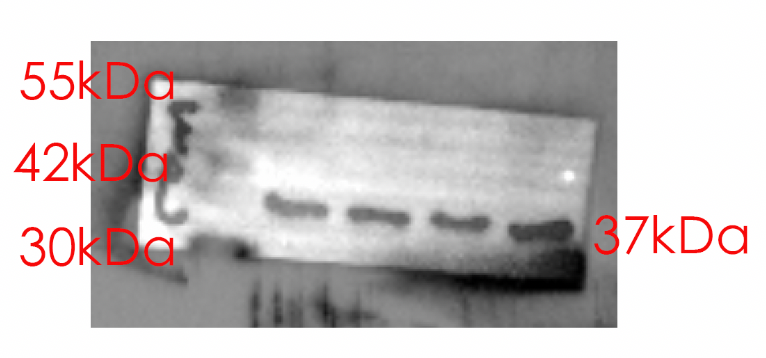


repeat 2


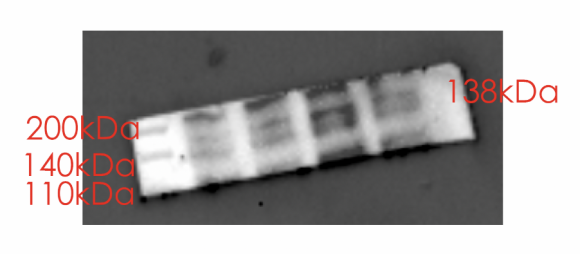

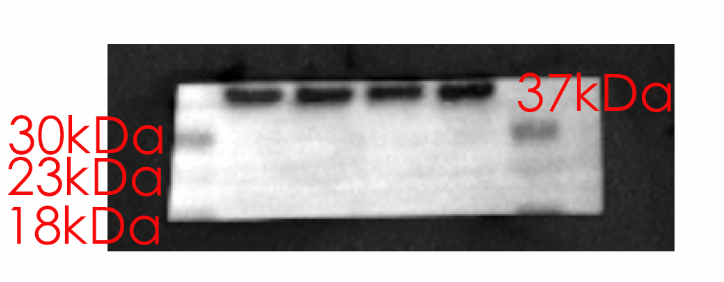


repeat 3


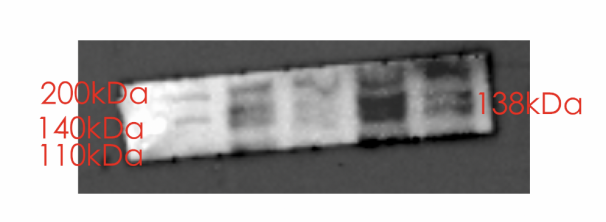

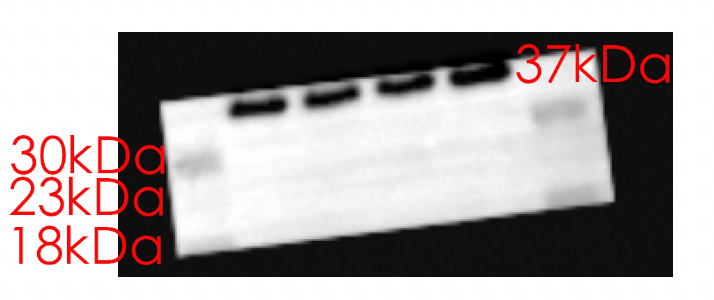


repeat 4


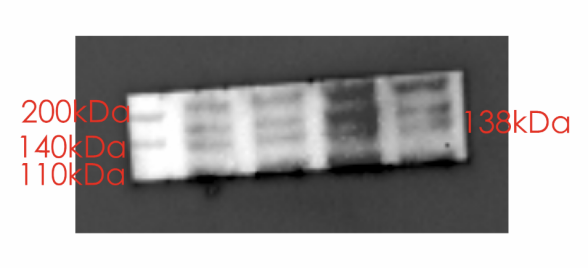

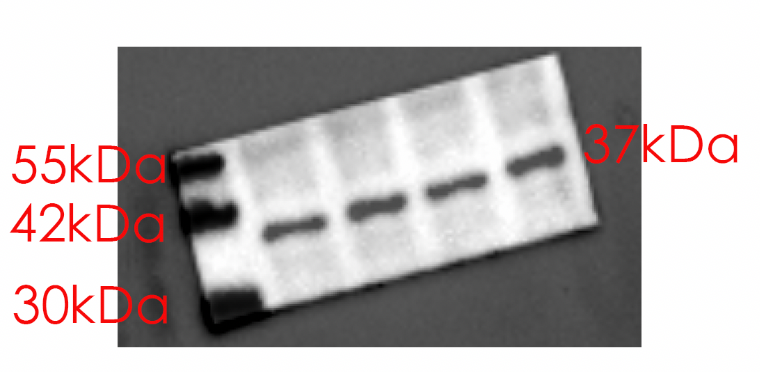


α-SMA GAPDH

| USP38fl/fl Sham | USP38cko Sham | USP38fl/fl CKD | USP38ckol CKD |
| --- | --- | --- | --- |

repeat 1


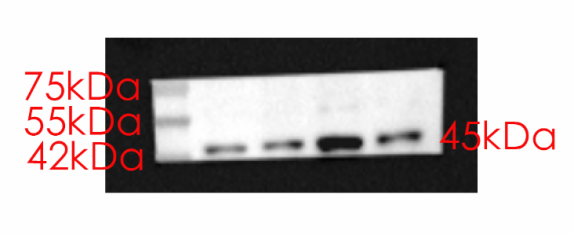

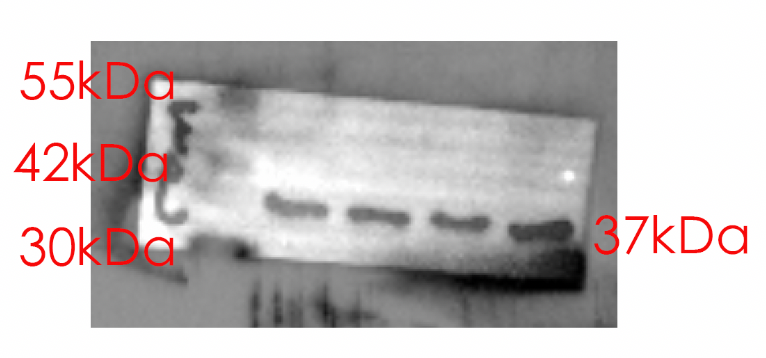


repeat 2


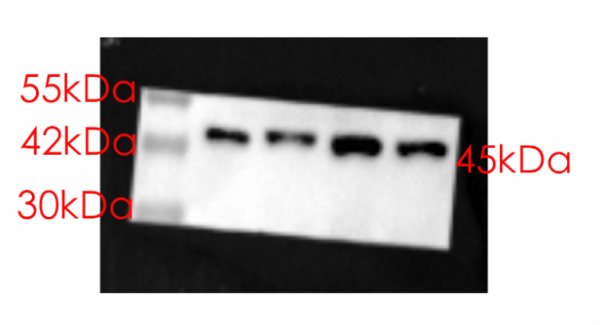

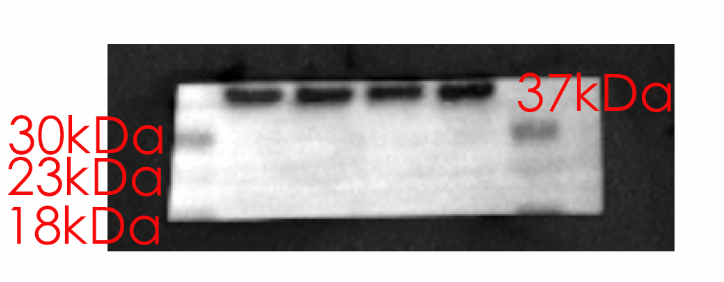


repeat 3


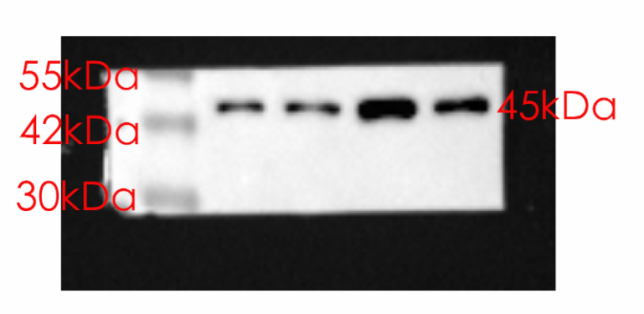

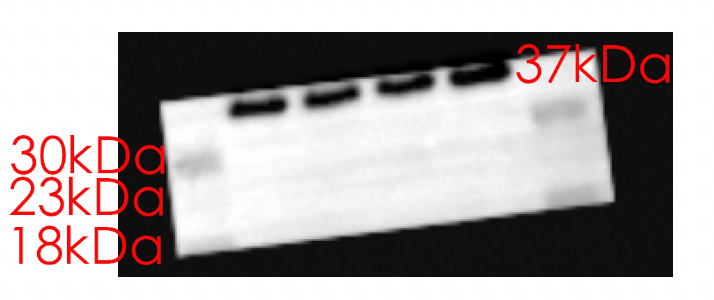


repeat 4


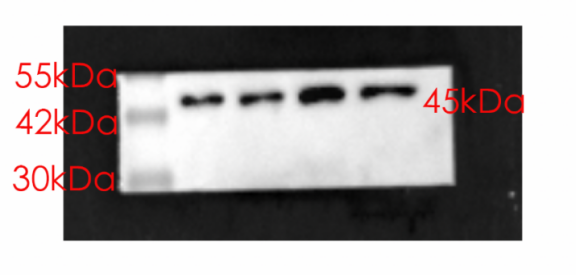

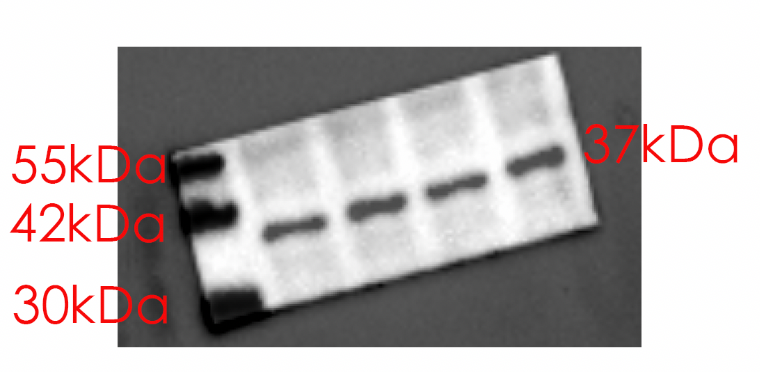


Figure 3G

collagen I GAPDH

| AdshRNA Vehicle | AdshUsp38 Vehicle | AdshRNA IS | AdshUsp38 IS |
| --- | --- | --- | --- |

repeat 1


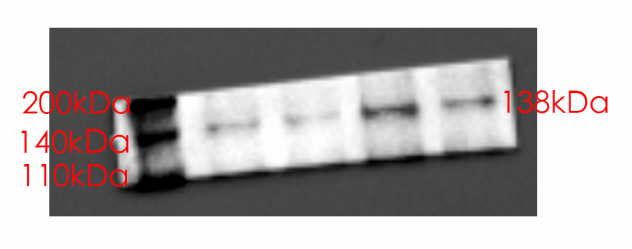

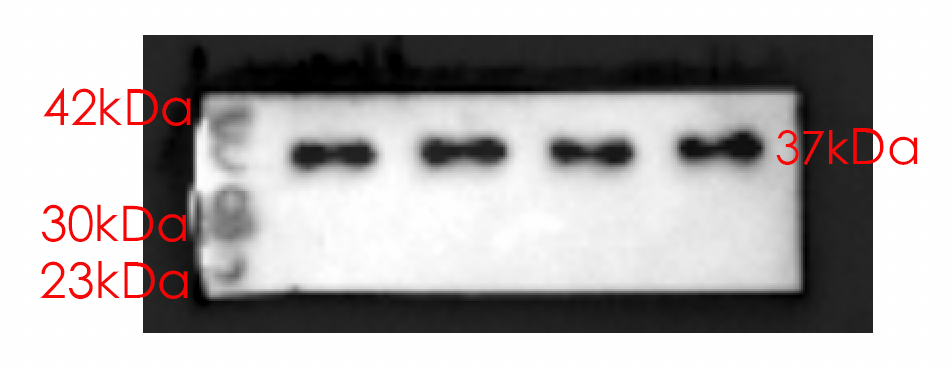


repeat 2


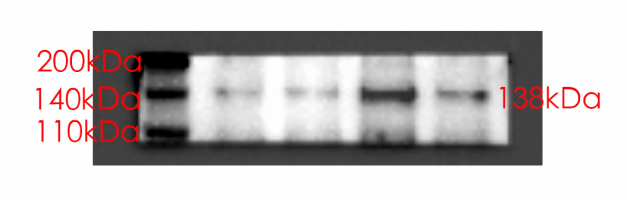

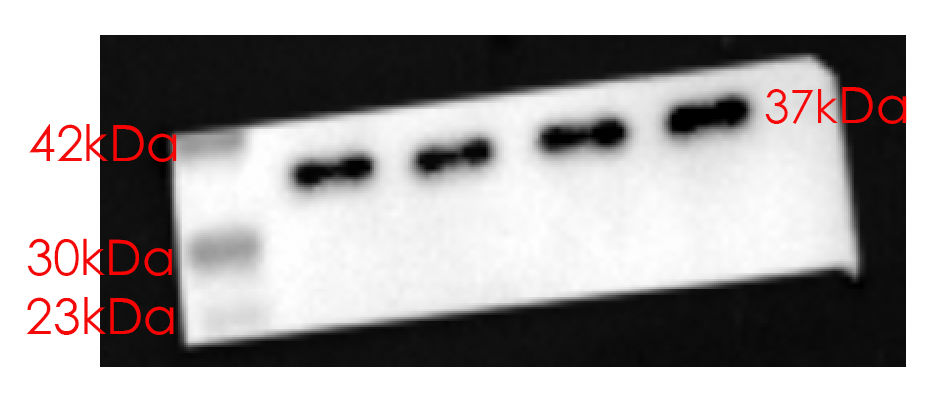


repeat 3


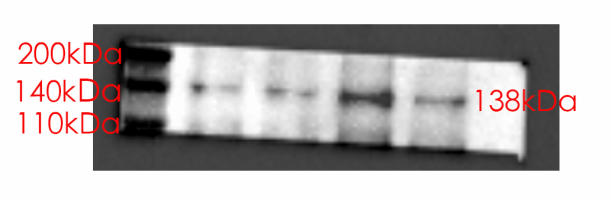

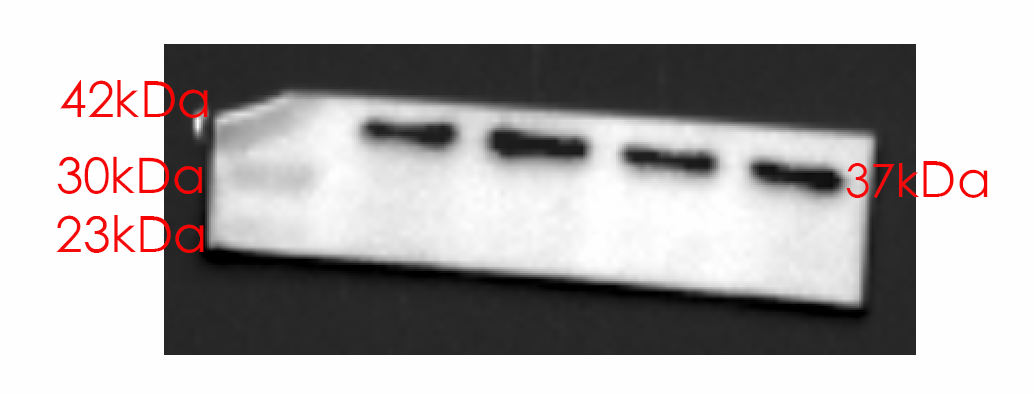


collagen III GAPDH

| AdshRNA Vehicle | AdshUsp38 Vehicle | AdshRNA IS | AdshUsp38 IS |
| --- | --- | --- | --- |

repeat 1


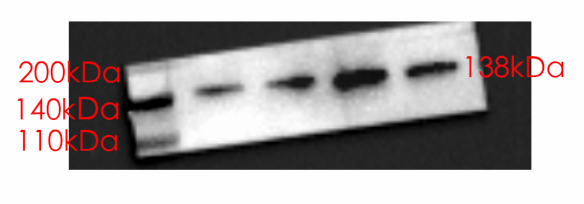

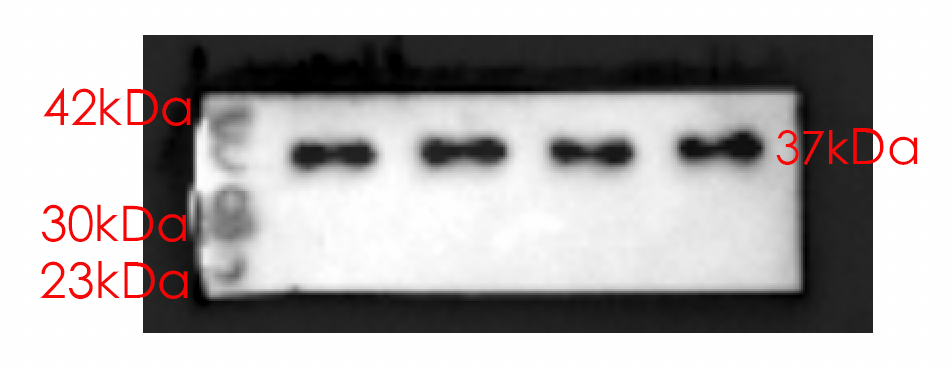


repeat 2


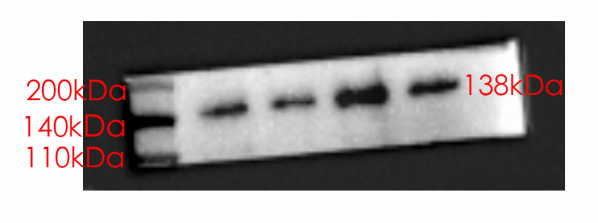

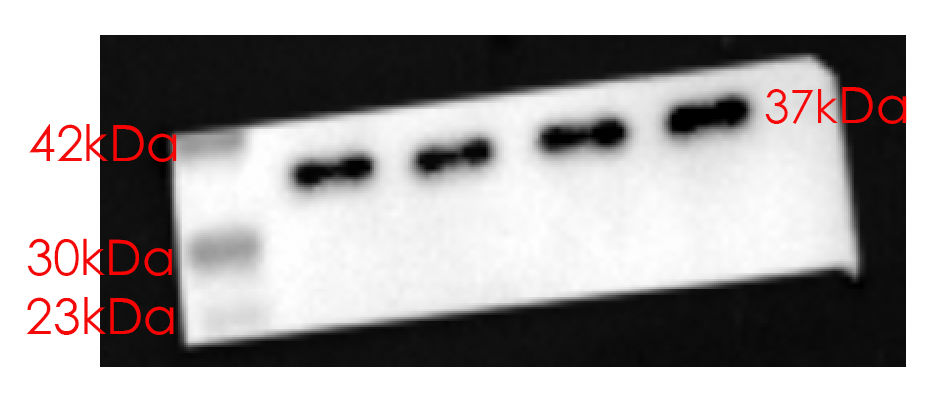


repeat 3


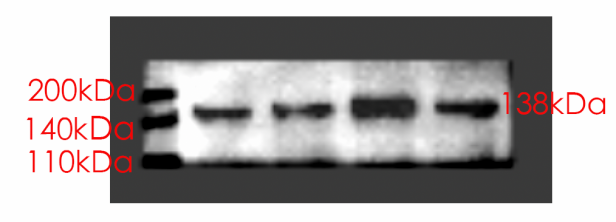

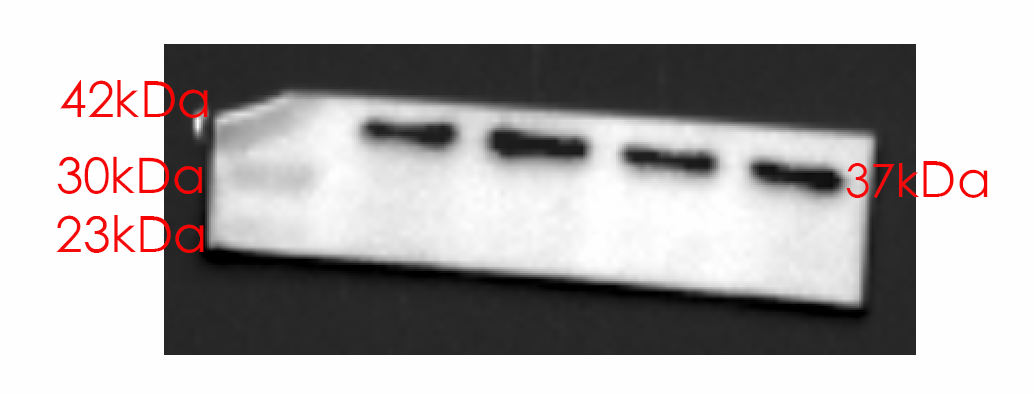


α-SMA GAPDH

| AdshRNA Vehicle | AdshUsp38 Vehicle | AdshRNA IS | AdshUsp38 IS |
| --- | --- | --- | --- |

repeat 1


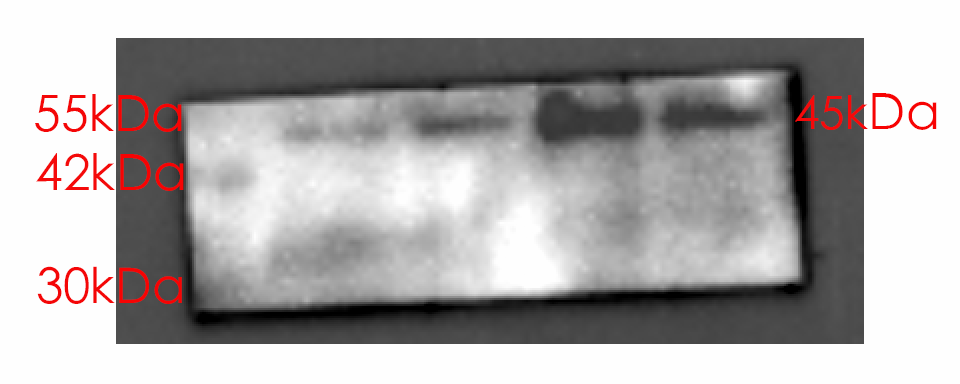

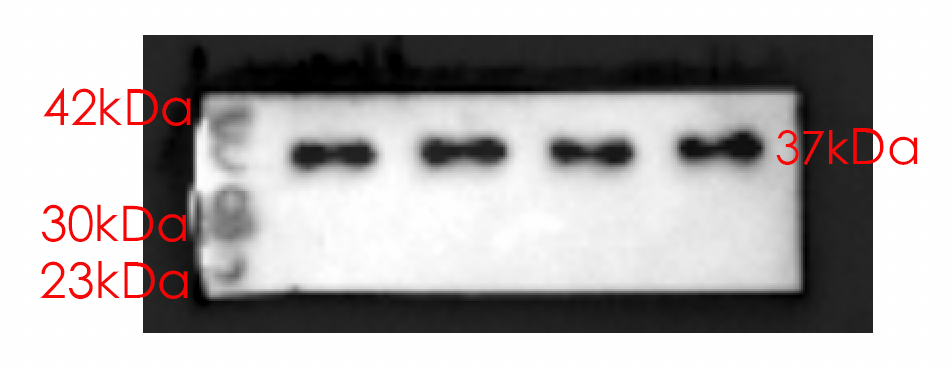


repeat 2


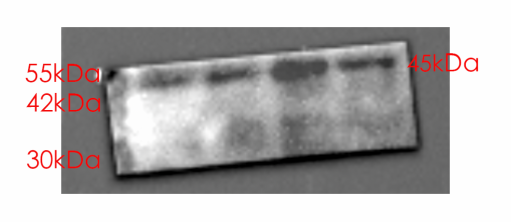

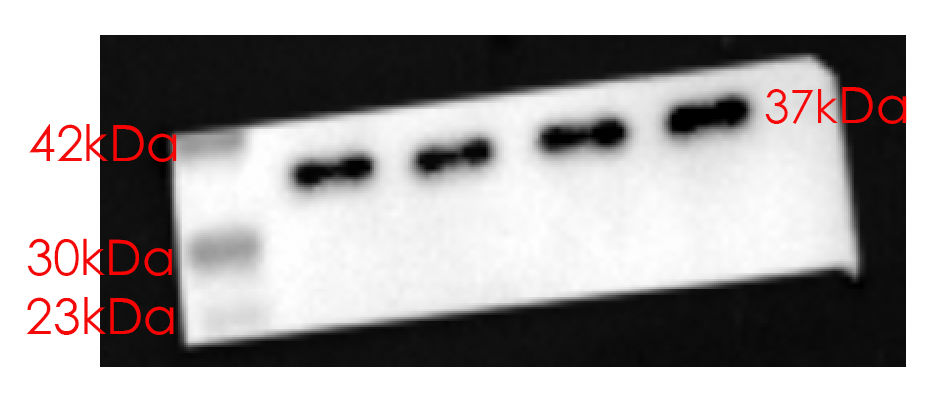


repeat 3


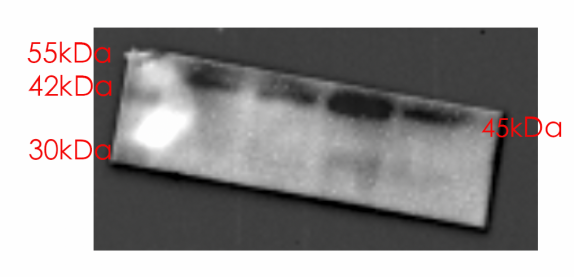

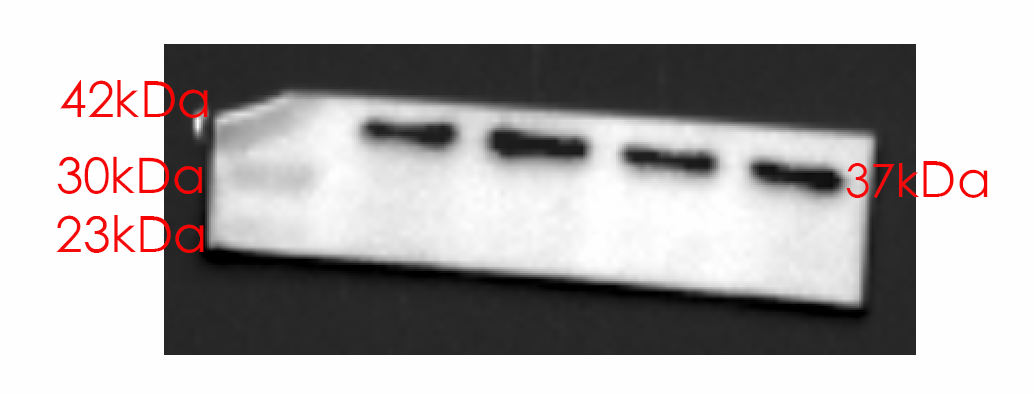


Figure 4F

collagen I GAPDH

| NTG Sham | USP38-TG Sham | NTG CKD | USP38-TG CKD |
| --- | --- | --- | --- |

repeat 1


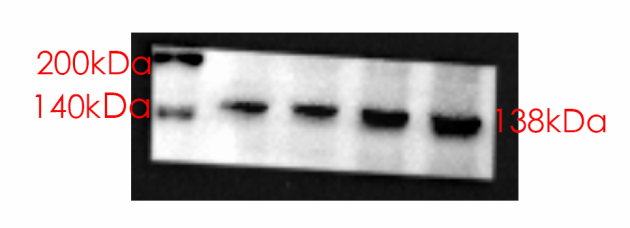

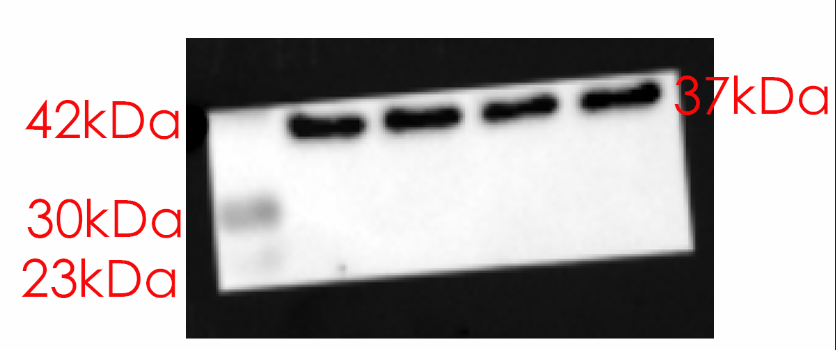


repeat 2


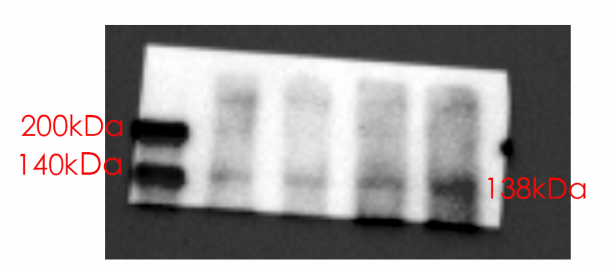

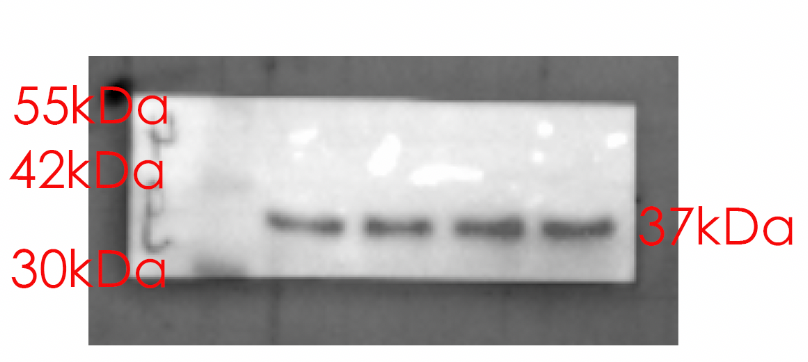


repeat 3


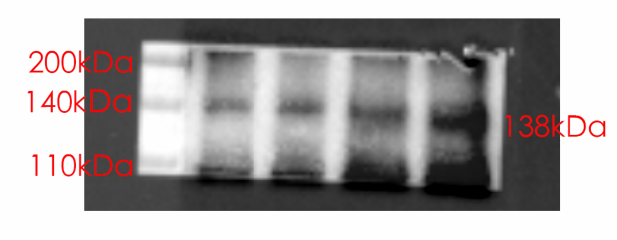

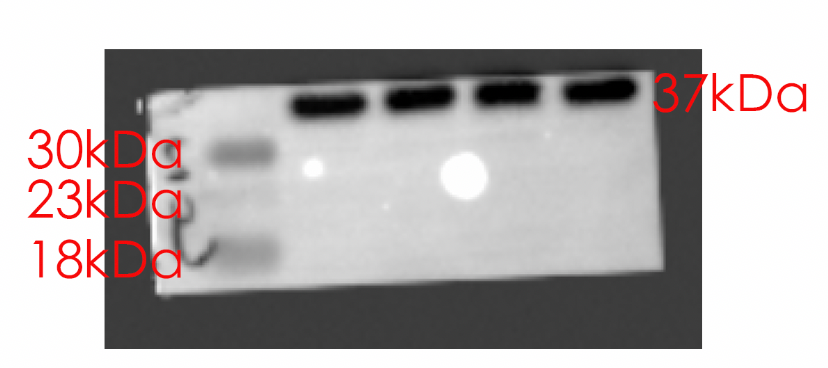


repeat 4


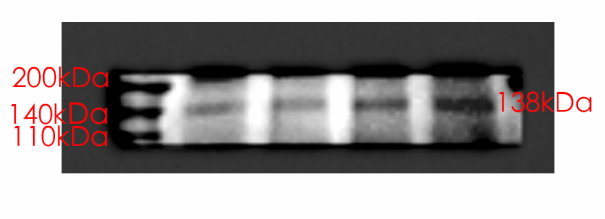

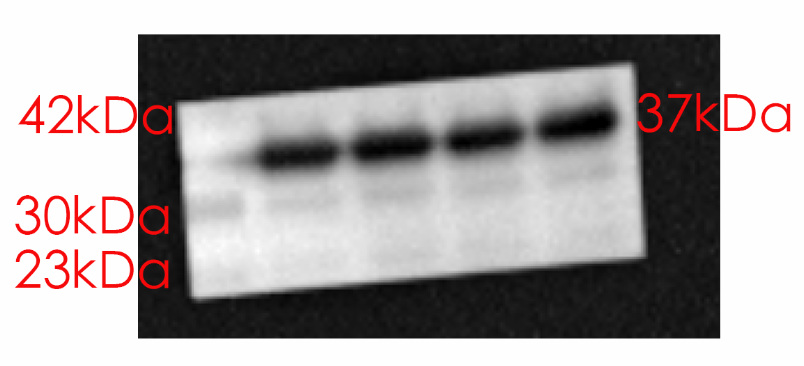


collagen III GAPDH

| NTG Sham | USP38-TG Sham | NTG CKD | USP38-TG CKD |
| --- | --- | --- | --- |

repeat 1


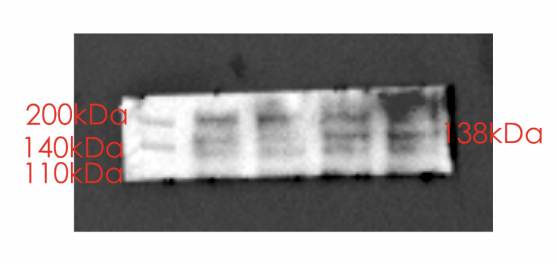

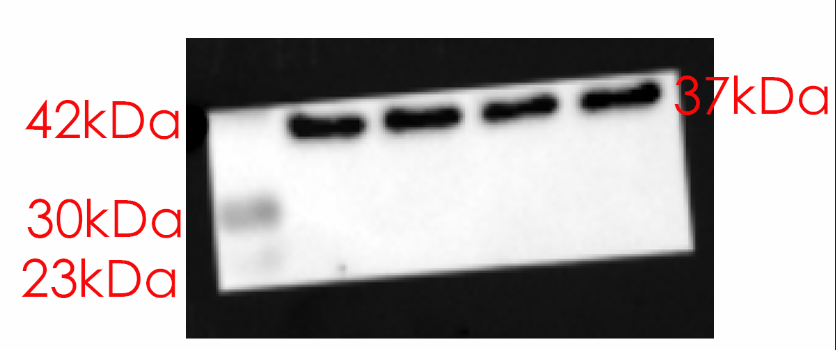


repeat 2


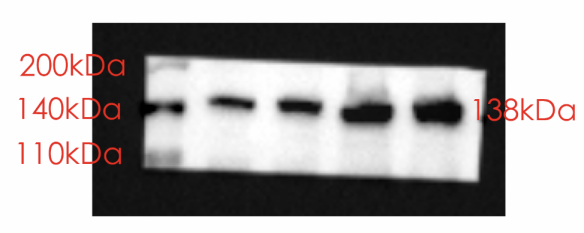

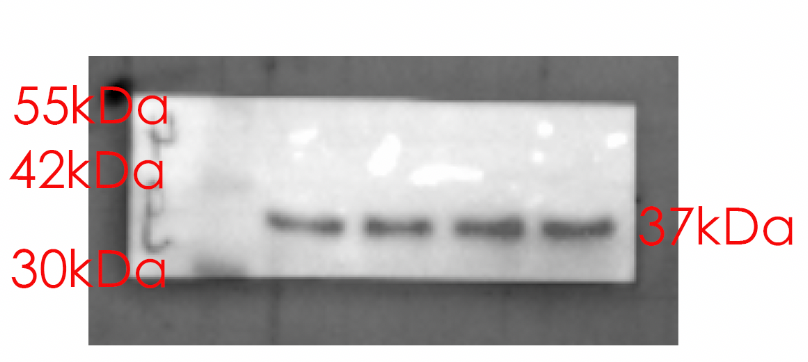


repeat 3


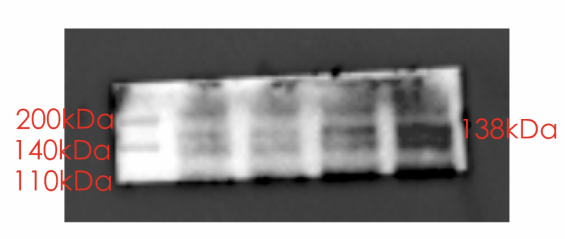

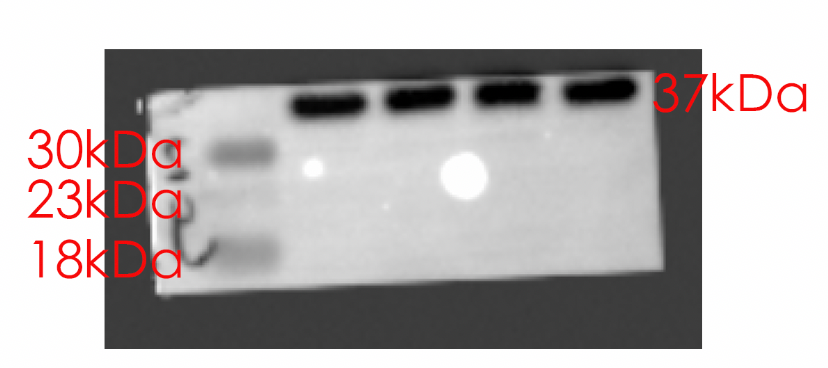


repeat 4


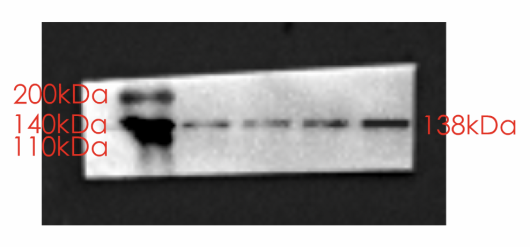

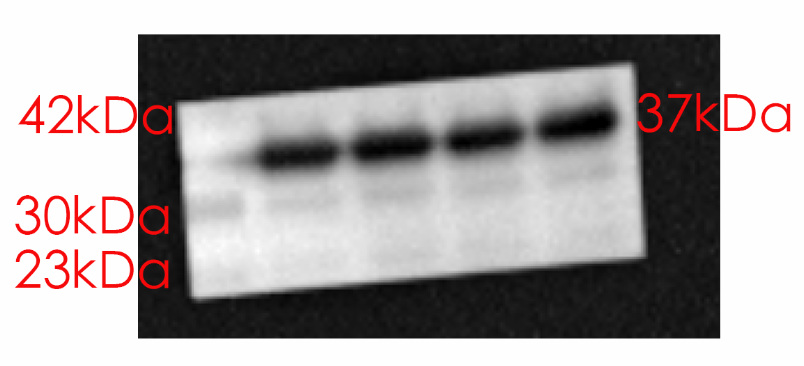


α-SMA GAPDH

| NTG Sham | USP38-TG Sham | NTG CKD | USP38-TG CKD |
| --- | --- | --- | --- |

repeat 1


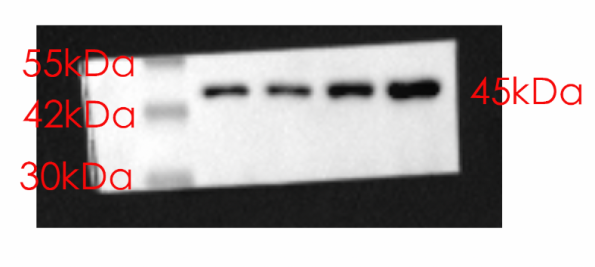

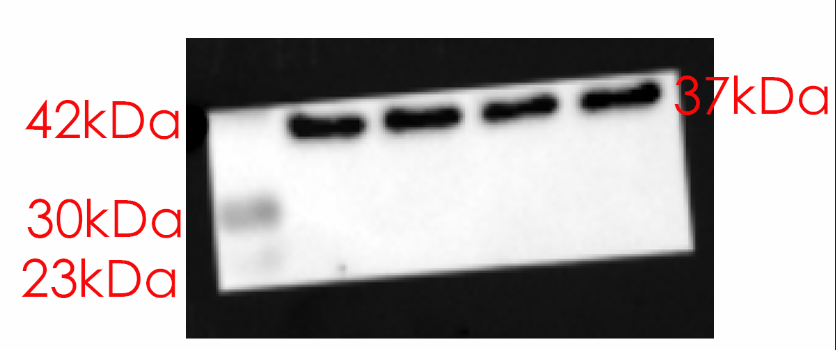


repeat 2


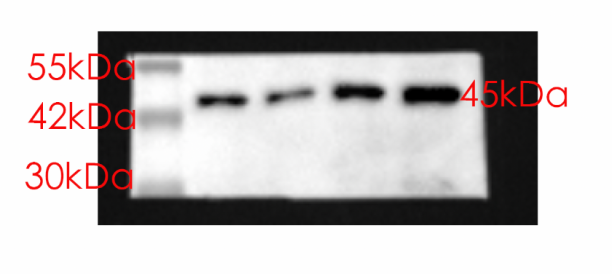

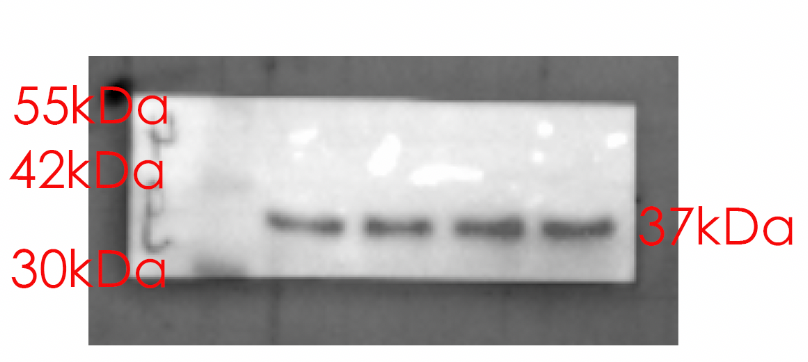


repeat 3


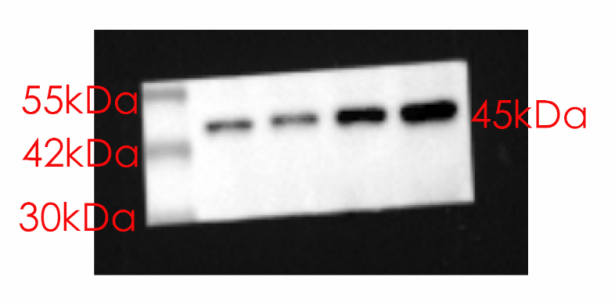

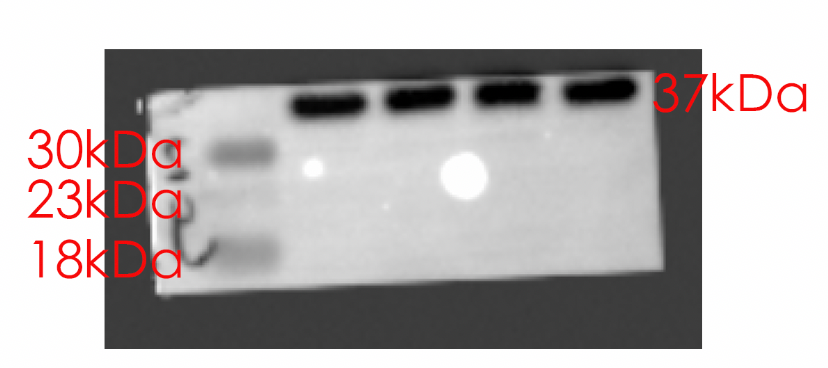


repeat 4


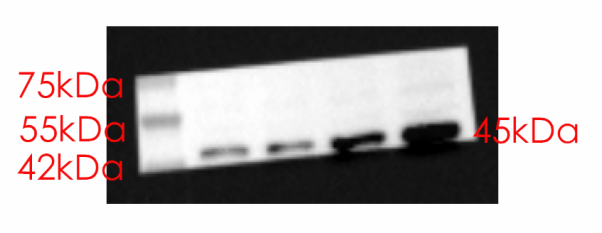

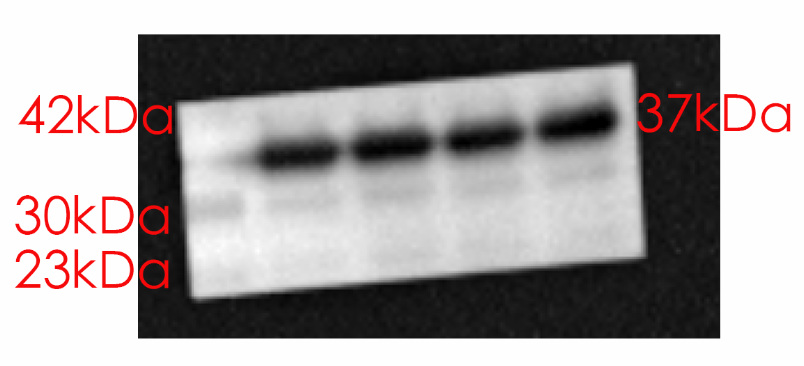


Figure 4G

collagen I GAPDH

| AdGFP Vehicle | AdUsp38 Vehicle | AdGFP IS | AdUsp38 IS |
| --- | --- | --- | --- |

repeat 1


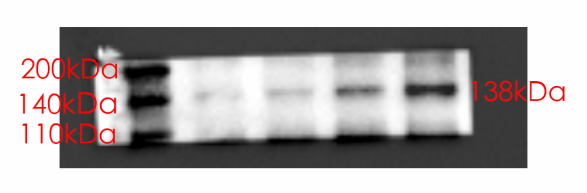

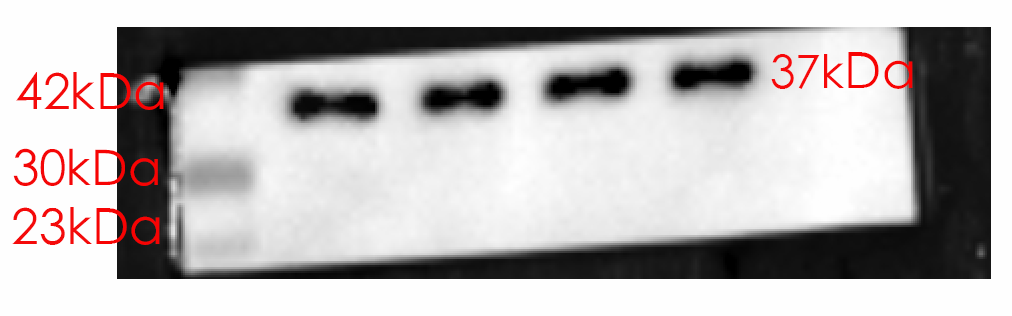


repeat 2


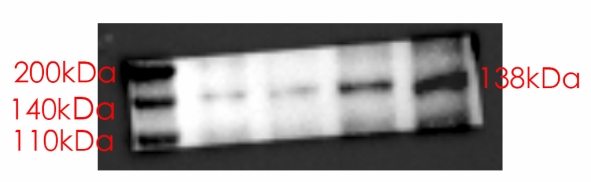

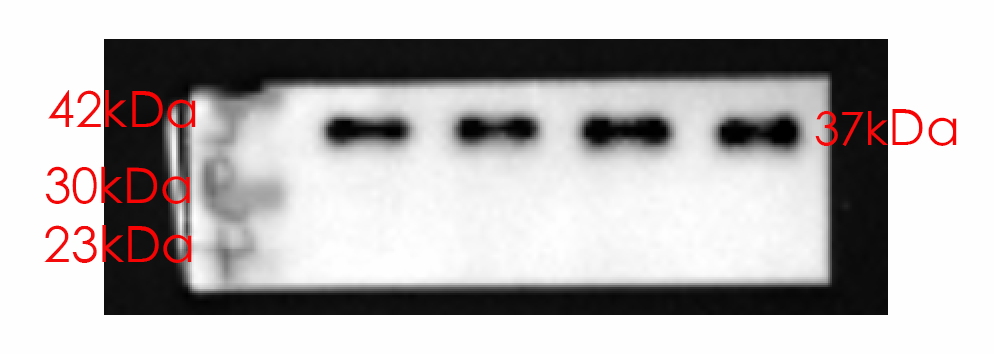


repeat 3


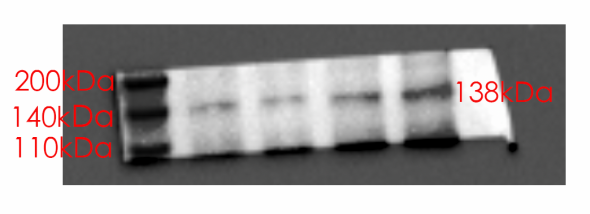

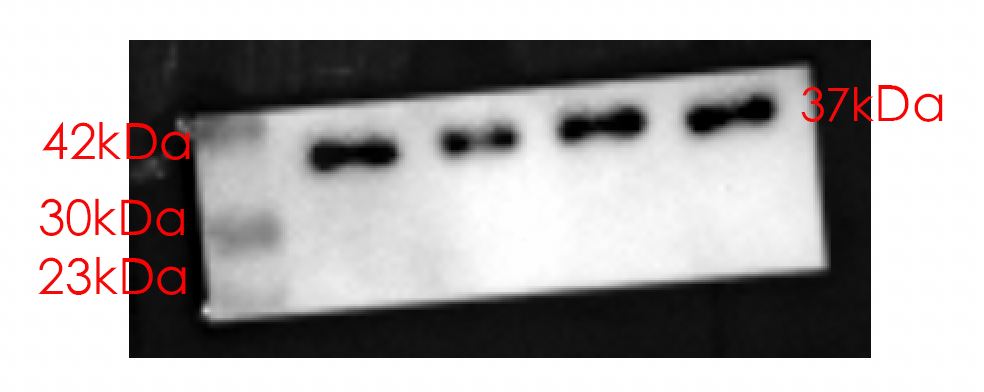


collagen III GAPDH

| AdGFP Vehicle | AdUsp38 Vehicle | AdGFP IS | AdUsp38 IS |
| --- | --- | --- | --- |

repeat 1


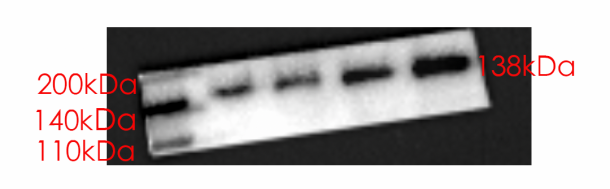

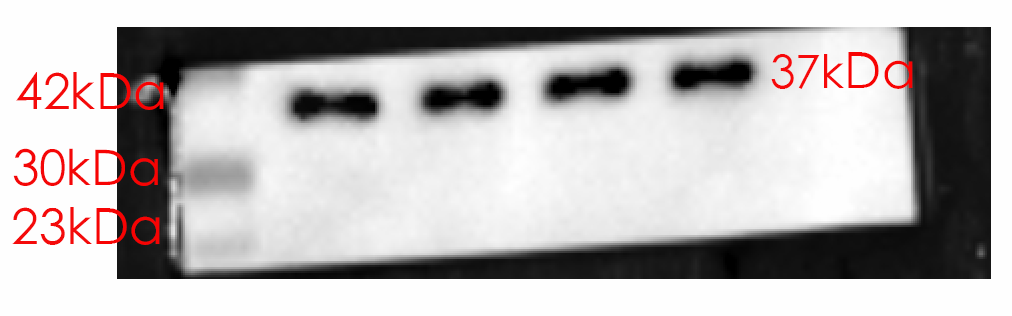


repeat 2


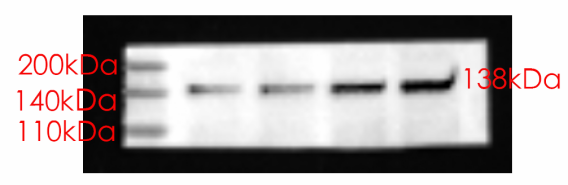

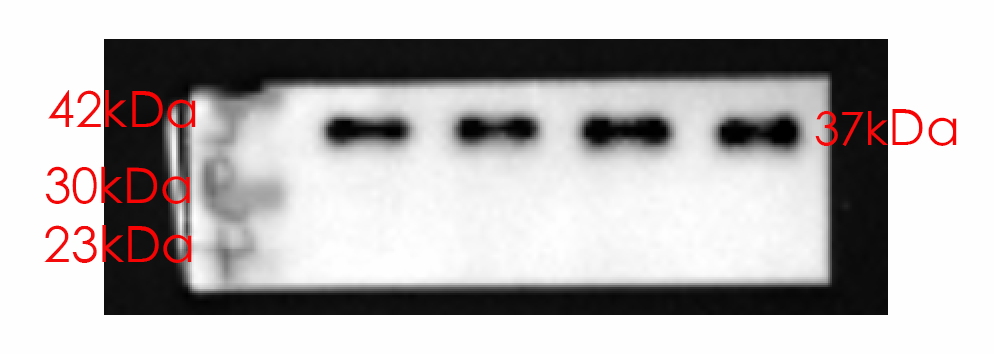


repeat 3


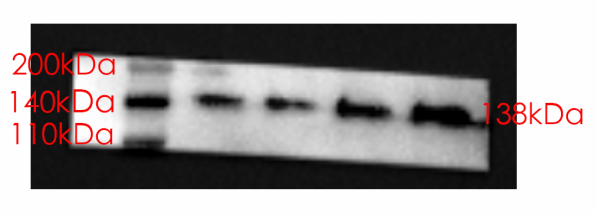

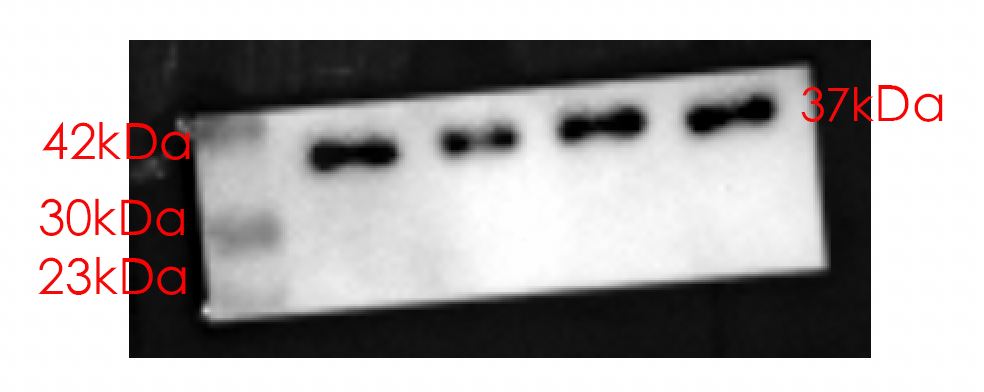


α-SMA GAPDH

| AdGFP Vehicle | AdUsp38 Vehicle | AdGFP IS | AdUsp38 IS |
| --- | --- | --- | --- |

repeat 1


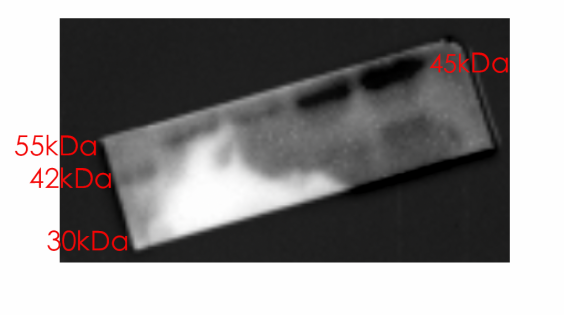

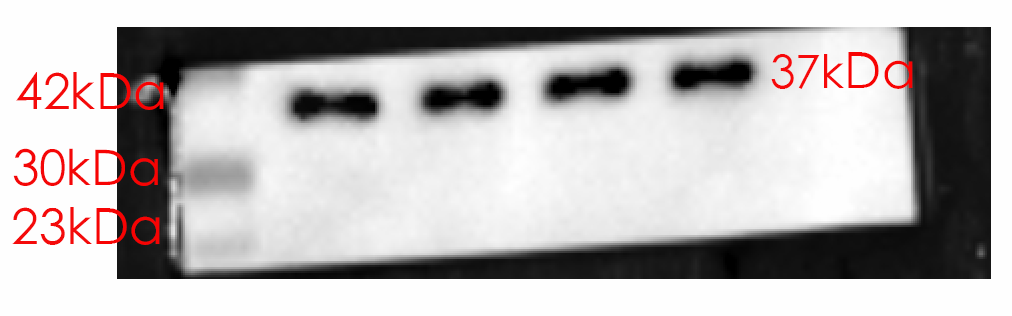


repeat 2


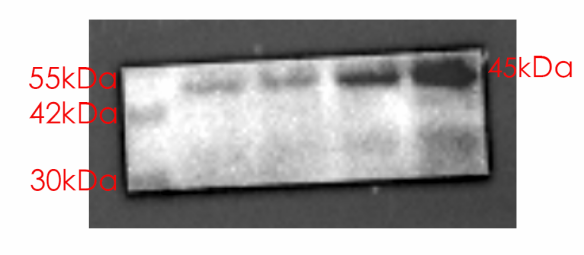

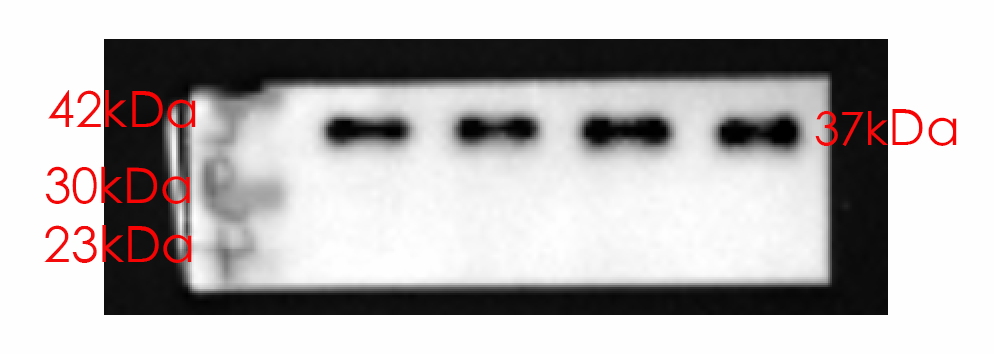


repeat 3


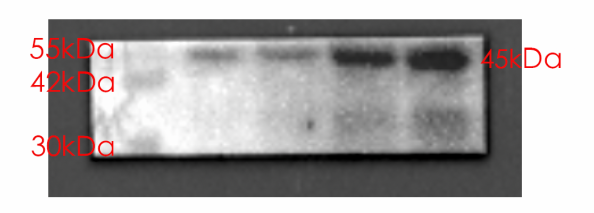

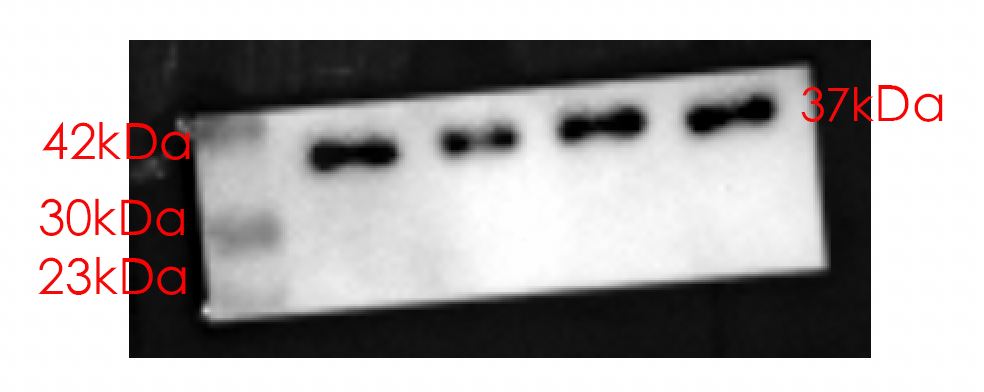


Figure 5B

CX43 GAPDH

| USP38fl/fl Sham | USP38cko Sham | USP38fl/fl CKD | USP38ckol CKD |
| --- | --- | --- | --- |

repeat 1;2


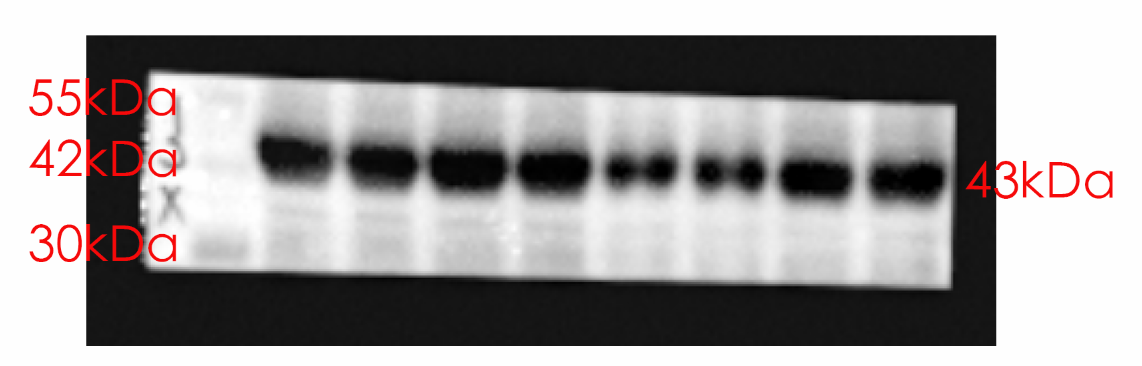


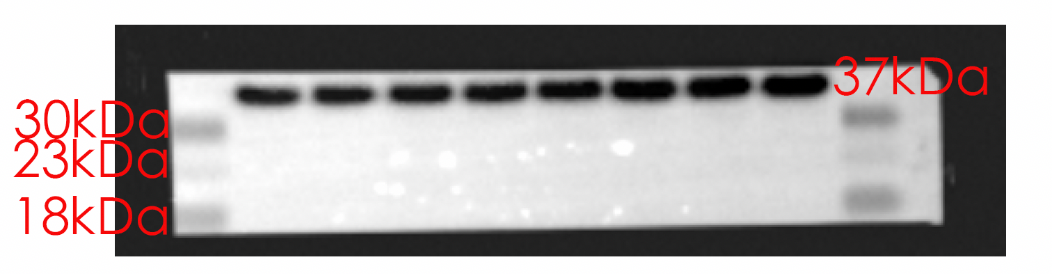


repeat 3


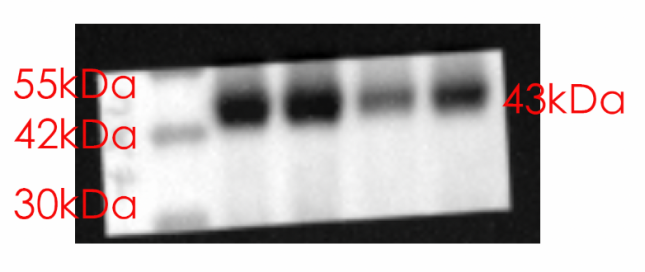

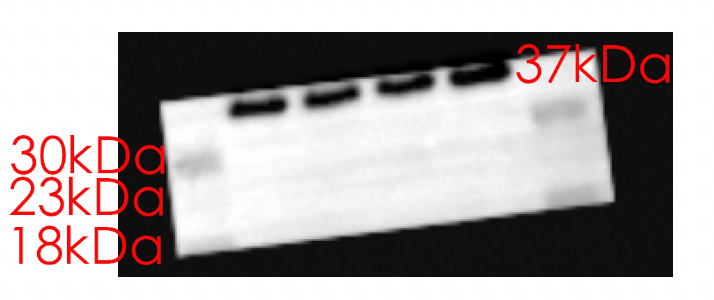


repeat 4


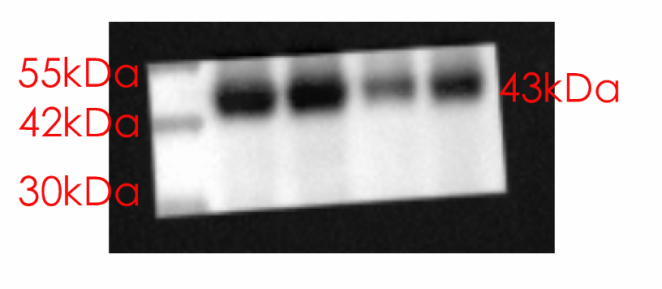

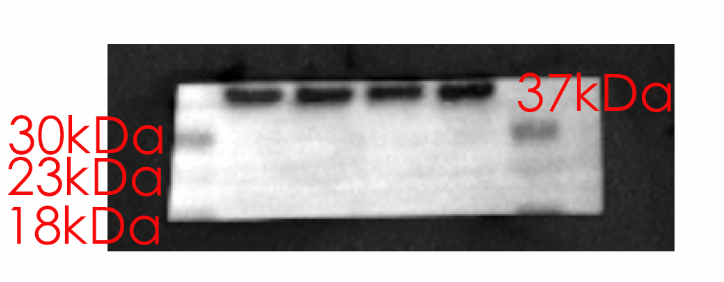


CX40 GAPDH

| USP38fl/fl Sham | USP38cko Sham | USP38fl/fl CKD | USP38ckol CKD |
| --- | --- | --- | --- |

repeat 1


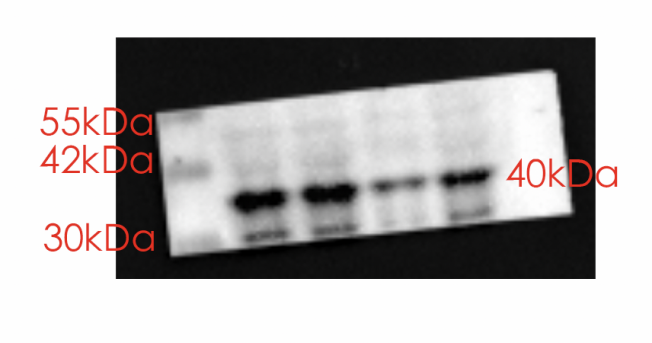

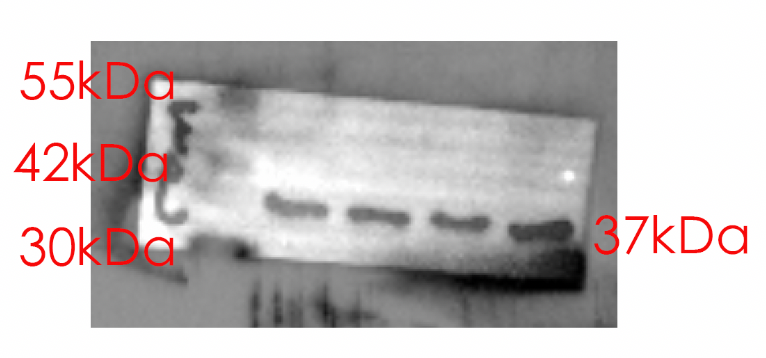


repeat 2


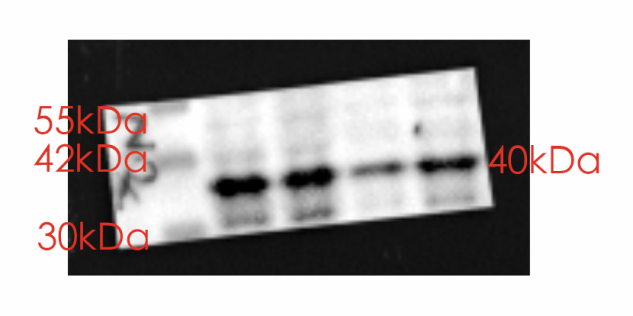

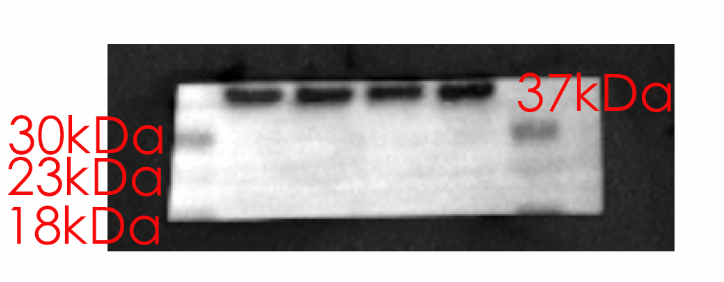


repeat 3


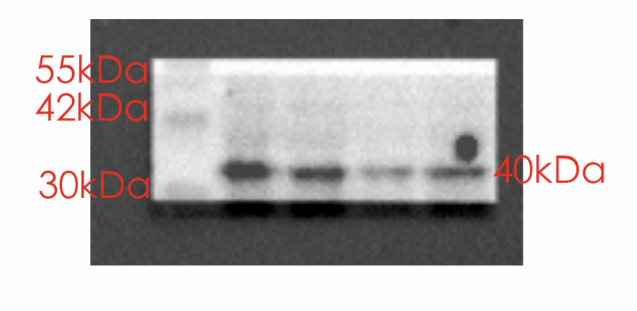

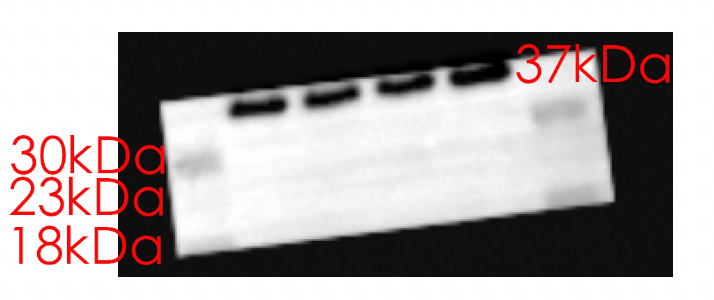


repeat 4

Figure 5D

CX43 GAPDH

| NTG Sham | USP38-TG Sham | NTG CKD | USP38-TG CKD |
| --- | --- | --- | --- |

repeat 1;2

repeat 3

repeat 4

CX40 GAPDH

| NTG Sham | USP38-TG Sham | NTG CKD | USP38-TG CKD |
| --- | --- | --- | --- |

repeat 1

repeat 2

repeat 3

repeat 4

Figure 6A

SMAD2 GAPDH

| USP38fl/fl Sham | USP38cko Sham | USP38fl/fl CKD | USP38ckol CKD |
| --- | --- | --- | --- |

repeat 1;2

repeat 3

repeat 4

p-SMAD2 GAPDH

| USP38fl/fl Sham | USP38cko Sham | USP38fl/fl CKD | USP38ckol CKD |
| --- | --- | --- | --- |

repeat 1

repeat 2

repeat 3

repeat 4

SMAD3 GAPDH

| USP38fl/fl Sham | USP38cko Sham | USP38fl/fl CKD | USP38ckol CKD |
| --- | --- | --- | --- |

repeat 1;2

repeat 3

repeat 4

p-SMAD3 GAPDH

| USP38fl/fl Sham | USP38cko Sham | USP38fl/fl CKD | USP38ckol CKD |
| --- | --- | --- | --- |

repeat 1

repeat 2

repeat 3

repeat 4

TGF-β GAPDH

| USP38fl/fl Sham | USP38cko Sham | USP38fl/fl CKD | USP38ckol CKD |
| --- | --- | --- | --- |

repeat 1

repeat 2

repeat 3；4

Figure 6B

SMAD2 GAPDH

| NTG Sham | USP38-TG Sham | NTG CKD | USP38-TG CKD |
| --- | --- | --- | --- |

repeat 1;2

repeat 3

repeat 4

p-SMAD2 GAPDH

| NTG Sham | USP38-TG Sham | NTG CKD | USP38-TG CKD |
| --- | --- | --- | --- |

repeat 1

repeat 2

repeat 3

repeat 4

SMAD3 GAPDH

| NTG Sham | USP38-TG Sham | NTG CKD | USP38-TG CKD |
| --- | --- | --- | --- |

repeat 1;2

repeat 3

repeat 4

p-SMAD3 GAPDH

| NTG Sham | USP38-TG Sham | NTG CKD | USP38-TG CKD |
| --- | --- | --- | --- |

repeat 1

repeat 2

repeat 3

repeat 4

TGF-β GAPDH

| NTG Sham | USP38-TG Sham | NTG CKD | USP38-TG CKD |
| --- | --- | --- | --- |

repeat 1

repeat 2;3

repeat 4

Figure 6C

p-SMAD2 GAPDH

| AdshRNA Vehicle | AdshUsp38 Vehicle | AdshRNA IS | AdshUsp38 IS |
| --- | --- | --- | --- |

repeat 1

SMAD2 GAPDH

| AdshRNA Vehicle | AdshUsp38 Vehicle | AdshRNA IS | AdshUsp38 IS |
| --- | --- | --- | --- |

repeat 1

p-SMAD3 GAPDH

| AdshRNA Vehicle | AdshUsp38 Vehicle | AdshRNA IS | AdshUsp38 IS |
| --- | --- | --- | --- |

repeat 1

SMAD3 GAPDH

| AdshRNA Vehicle | AdshUsp38 Vehicle | AdshRNA IS | AdshUsp38 IS |
| --- | --- | --- | --- |

repeat 1

TGF-β GAPDH

| AdshRNA Vehicle | AdshUsp38 Vehicle | AdshRNA IS | AdshUsp38 IS |
| --- | --- | --- | --- |

repeat 1

Figure 6D

p-SMAD2 GAPDH

| AdGFP Vehicle | AdUsp38 Vehicle | AdGFP IS | AdUsp38 IS |
| --- | --- | --- | --- |

repeat 1

SMAD2 GAPDH

| AdGFP Vehicle | AdUsp38 Vehicle | AdGFP IS | AdUsp38 IS |
| --- | --- | --- | --- |

repeat 1

p-SMAD3 GAPDH

| AdGFP Vehicle | AdUsp38 Vehicle | AdGFP IS | AdUsp38 IS |
| --- | --- | --- | --- |

repeat 1

SMAD3 GAPDH

| AdGFP Vehicle | AdUsp38 Vehicle | AdGFP IS | AdUsp38 IS |
| --- | --- | --- | --- |

repeat 1

TGF-β GAPDH

| AdGFP Vehicle | AdUsp38 Vehicle | AdGFP IS | AdUsp38 IS |
| --- | --- | --- | --- |

repeat 1

Figure 7F

Input: Flag-STRAP

Input: MYC-USP38

IP: MYC-USP38

IP: Flag-STRAP

Figure 7F

Input: Flag-STRAP

Input: MYC-USP38

IP: MYC-USP38

IP: Flag-STRAP

Figure 8A

STRAP GAPDH

| USP38fl/fl Sham | USP38cko Sham | USP38fl/fl CKD | USP38ckol CKD |
| --- | --- | --- | --- |

repeat 1;2

repeat 3

repeat 4

Figure 8B

STRAP GAPDH

| AdshRNA Vehicle | AdshUsp38 Vehicle | AdshRNA IS | AdshUsp38 IS |
| --- | --- | --- | --- |

repeat 1

Figure 8E

STRAP GAPDH

| NTG Sham | USP38-TG Sham | NTG CKD | USP38-TG CKD |
| --- | --- | --- | --- |

repeat 1;2

repeat 3

repeat 4

Figure 8F

STRAP GAPDH

| AdGFP Vehicle | AdUsp38 Vehicle | AdGFP IS | AdUsp38 IS |
| --- | --- | --- | --- |

repeat 1

Figure 8I

IP: Ub-STRAP

IP: STRAP

Input: Ub

Input: STRAP

Input: GAPDH

Figure 8J

IP: Ub-STRAP

IP: STRAP

Input: Ub

Input: STRAP

Input: GAPDH

Figure 9B

STRAP GAPDH

| TG CKD  rAAV9-shRNA | TG CKD  rAAV9-shRNA | TG CKD  rAAV9-shSTRAP | TG CKD  rAAV9-shSTRAP |
| --- | --- | --- | --- |

repeat 1，2

repeat 3，4

Figure 9H

collagen I GAPDH

| TG CKD  rAAV9-shRNA | TG CKD  rAAV9-shRNA | TG CKD  rAAV9-shSTRAP | TG CKD  rAAV9-shSTRAP |
| --- | --- | --- | --- |

repeat 1，2

repeat 3，4

collagen III GAPDH

| TG CKD  rAAV9-shRNA | TG CKD  rAAV9-shRNA | TG CKD  rAAV9-shSTRAP | TG CKD  rAAV9-shSTRAP |
| --- | --- | --- | --- |

repeat 1，2

repeat 3，4

α-SMA GAPDH

| TG CKD  rAAV9-shRNA | TG CKD  rAAV9-shRNA | TG CKD  rAAV9-shSTRAP | TG CKD  rAAV9-shSTRAP |
| --- | --- | --- | --- |

repeat 1，2

Figure 9I

p-SMAD3 GAPDH

| TG CKD  rAAV9-shRNA | TG CKD  rAAV9-shRNA | TG CKD  rAAV9-shSTRAP | TG CKD  rAAV9-shSTRAP |
| --- | --- | --- | --- |

repeat 1，2

repeat 3，4

SMAD3 GAPDH

| TG CKD  rAAV9-shRNA | TG CKD  rAAV9-shRNA | TG CKD  rAAV9-shSTRAP | TG CKD  rAAV9-shSTRAP |
| --- | --- | --- | --- |

repeat 1，2

repeat 3，4

p-SMAD2 GAPDH

| TG CKD  rAAV9-shRNA | TG CKD  rAAV9-shRNA | TG CKD  rAAV9-shSTRAP | TG CKD  rAAV9-shSTRAP |
| --- | --- | --- | --- |

repeat 1，2

repeat 3，4

SMAD2 GAPDH

| TG CKD  rAAV9-shRNA | TG CKD  rAAV9-shRNA | TG CKD  rAAV9-shSTRAP | TG CKD  rAAV9-shSTRAP |
| --- | --- | --- | --- |

repeat 1，2

repeat 3，4

TGF-β GAPDH

| TG CKD  rAAV9-shRNA | TG CKD  rAAV9-shRNA | TG CKD  rAAV9-shSTRAP | TG CKD  rAAV9-shSTRAP |
| --- | --- | --- | --- |

repeat 1，2

repeat 3，4

***Supplementary Figure S1G***

| USP38 | | | |
| --- | --- | --- | --- |
| CKD 0W | CKD 2W | CKD 4W | CKD 4W+AST120 |
| repeat 1 | | | |
|  | | | |
| repeat 2 | | | |
|  | | | |
| repeat 3 | | | |
|  | | | |
| repeat 4 | | | |
|  | | | |
| GAPDH | | | |
| repeat 1 | | | |
|  | | | |
| repeat 2 | | | |
|  | | | |
| repeat 3 | | | |
|  | | | |
| repeat 4 | | | |
|  | | | |

***Supplementary Figure S2***

USP38

| Heart | | Liver | | Kidney | | Lung | |
| --- | --- | --- | --- | --- | --- | --- | --- |
| USP38fl/fl | USP38cko | USP38fl/fl | USP38cko | USP38fl/fl | USP38cko | USP38fl/fl | USP38cko |

| Heart | | Liver | | Kidney | | Lung | |
| --- | --- | --- | --- | --- | --- | --- | --- |
| NTG | USP38-TG | NTG | USP38-TG | NTG | USP38-TG | NTG | USP38-TG |

| ***Supplementary Figure S4A***  USP38 | | | |
| --- | --- | --- | --- |
| CKD-FLOX | | CKD-CKO | |
| SR | AF | SR | AF |
| repeat 1 | | | |
|  | | | |
| repeat 2 | | | |
|  | | | |
| repeat 3 | | | |
|  | | | |
| GAPDH | | | |
| repeat 1 | | | |
|  | | | |
| repeat 2 | | | |
|  | | | |
| repeat 3 | | | |
|  | | | |

***Supplementary Figure S4B***

| USP38 | | | |
| --- | --- | --- | --- |
| CKD-NTG | | CKD-TG | |
| SR | AF | SR | AF |
| repeat 1 | | | |
|  | | | |
| repeat 2 | | | |
|  | | | |
| repeat 3 | | | |
|  | | | |
| GAPDH | | | |
| repeat 1 | | | |
|  | | | |
| repeat 2 | | | |
|  | | | |
| repeat 3 | | | |
|  | | | |

***Supplementary Figure S6***

RYR2 GAPDH

| USP38fl/fl Sham | USP38cko Sham | USP38fl/fl CKD | USP38ckol CKD |
| --- | --- | --- | --- |

repeat 1

repeat 2

repeat 3

repeat 4

p-RYR2 GAPDH

| USP38fl/fl Sham | USP38cko Sham | USP38fl/fl CKD | USP38ckol CKD |
| --- | --- | --- | --- |

repeat 1

repeat 2

repeat 3

repeat 4

SERCA2a GAPDH

| USP38fl/fl Sham | USP38cko Sham | USP38fl/fl CKD | USP38ckol CKD |
| --- | --- | --- | --- |

repeat 1

repeat 2

repeat 3

repeat 4

p-PLB GAPDH

| USP38fl/fl Sham | USP38cko Sham | USP38fl/fl CKD | USP38ckol CKD |
| --- | --- | --- | --- |

repeat 1;2

repeat 3

repeat 4

PLB GAPDH

| USP38fl/fl Sham | USP38cko Sham | USP38fl/fl CKD | USP38ckol CKD |
| --- | --- | --- | --- |

repeat 1;2

repeat 3

repeat 4

RYR2 GAPDH

| NTG Sham | USP38-TG Sham | NTG CKD | USP38-TG CKD |
| --- | --- | --- | --- |

repeat 1

repeat 2

repeat 3

repeat 4

p-RYR2 GAPDH

| NTG Sham | USP38-TG Sham | NTG CKD | USP38-TG CKD |
| --- | --- | --- | --- |

repeat 1

repeat 2

repeat 3

repeat 4

SERCA2a GAPDH

| NTG Sham | USP38-TG Sham | NTG CKD | USP38-TG CKD |
| --- | --- | --- | --- |

repeat 1

repeat 2

repeat 3

repeat 4

p-PLB GAPDH

| NTG Sham | USP38-TG Sham | NTG CKD | USP38-TG CKD |
| --- | --- | --- | --- |

repeat 1;2

repeat 3

repeat 4

PLB GAPDH

| NTG Sham | USP38-TG Sham | NTG CKD | USP38-TG CKD |
| --- | --- | --- | --- |

repeat 1;2

repeat 3

repeat 4

| ***Supplementary Figure S7A*** | | | |
| --- | --- | --- | --- |
| Input | | IP: STRAP | |
| NS | IS | NS | IS |
| USP38 |  |  |  |
| repeat 1 | | | |
|  | | | |
| repeat 2 | | | |
|  | | | |
| repeat 3 | | | |
|  | | | |
| STRAP | | | |
| repeat1 | | | |
|  | | | |
| repeat 2 | | | |
|  | | | |
| repeat 3 | | | |
|  | | | |
| ***Supplementary Figure S7B*** | | | |
| Input | | IP: USP38 | |
| NS | IS | NS | IS |
| USP38 |  |  |  |
| repeat 1 | | | |
|  | | | |
| repeat 2 | | | |
|  | | | |
| repeat 3 | | | |
|  | | | |
| STRAP | | | |
| repeat1 | | | |
|  | | | |
| repeat 2 | | | |
|  | | | |
| repeat 3 | | | |
|  | | | |
